# Genome-wide association studies of infant and toddler temperament in European and multi-ancestry populations

**DOI:** 10.1038/s41562-026-02486-5

**Published:** 2026-07-01

**Authors:** Anja Hollowell, Anna Gui, Emilie Wigdor, Morgan J. Morgan, Laurie J. Hannigan, Elizabeth C. Corfield, René Pool, Susanne Bruins, Helga Ask, Christel M. Middeldorp, Beate St Pourcain, Meike Bartels, Dorret I. Boomsma, Catharina A. Hartman, Aoi Noda, Ippei Takahashi, Mami Ishikuro, Taku Obara, Shinichi Kuriyama, Mary S. Mufford, Marilyn T. Lake, Dan J. Stein, Heather J. Zar, Nadia Hoffman, Elise B. Robinson, Anders D. Børglum, Xinhe Zhang, Varun Warrier, Autism Spectrum Disorder Working Group of the Psychiatric Genomics Consortium, Jakob Grove, Isabel Dziobek, Lauren Weiss, Melanie M. de Wit, Mohammed Uddin, Ole A. Andreassen, Richard J. L. Anney, Stephen W. Scherer, Susan S. Kuo, Wonuola A. Akingbuwa, Tomoki Arichi, Mark H. Johnson, Frank Dudbridge, Stephan J. Sanders, Alexandra Havdahl, Angelica Ronald

**Affiliations:** 1 Centre for Brain and Cognitive Development, Department of Psychological Sciences, Birkbeck University of London, London, UK; 2 School of Psychology, Faculty of Health and Medical Sciences, University of Surrey, Guildford, Surrey, UK; 3 Division of Psychiatry, University College London, London, UK; 4 Department of Systems Medicine, University of Rome Tor Vergata, Rome, Italy; 5 Institute of Developmental and Regenerative Medicine, Department of Paediatrics, University of Oxford, Oxford, UK; 6 Research Department, Lovisenberg Diaconal Hospital, Oslo, Norway; 7 PsychGen Centre for Genetic Epidemiology and Mental Health, Norwegian Institute of Public Health, Oslo, Norway; 8 Population Health Sciences, Bristol Medical School, University of Bristol, Bristol, UK; 9 MRC Integrative Epidemiology Unit, Bristol Medical School, University of Bristol, Bristol, UK; 10 Department of Biological Psychology, Vrije Universiteit Amsterdam, Amsterdam, the Netherlands; 11 Amsterdam Public Health Research Institute, Amsterdam, the Netherlands; 12 Department of Child Health and Development, Norwegian Institute of Public Health, Oslo, Norway; 13 PROMENTA Research Centre, Department of Psychology, University of Oslo, Oslo, Norway; 14 Department of Child and Adolescent Psychiatry and Psychology, Amsterdam Reproduction and Development Research Institute, Amsterdam Public Health Research Institute, Amsterdam UMC, Amsterdam, the Netherlands; 15 Arkin Mental Health Care, Amsterdam, the Netherlands; 16 Levvel, Academic Center for Child and Adolescent Psychiatry, Amsterdam, the Netherlands; 17 Child Health Research Centre, University of Queensland, Brisbane, Queensland Australia; 18 Child and Youth Mental Health Service, Children’s Health Queensland Hospital and Health Service, Brisbane, Queensland Australia; 19 Max Planck Institute for Psycholinguistics, Nijmegen, the Netherlands; 20 Donders Institute for Brain, Cognition and Behaviour, Radboud University, Nijmegen, the Netherlands; 21 Department of Complex Trait Genetics, Center for Neurogenomics and Cognitive Research, Amsterdam, Vrije Universiteit, Amsterdam, the Netherlands; 22 Amsterdam Reproduction and Development Research Institute, Amsterdam Public Health Research Institute, Amsterdam UMC, Amsterdam, the Netherlands; 23 University Medical Center Psychopathology and Emotion Regulation, Department of Psychiatry, University Medical Center Groningen, University of Groningen, Groningen, the Netherlands; 24 Division of Molecular Epidemiology, Tohoku Medical Megabank Organization, Tohoku University, Sendai, Japan; 25 Division of Molecular Epidemiology, Environment and Genome Research Center, Graduate School of Medicine, Tohoku University, Sendai, Japan; 26 Division of Human Genetics, National Health Laboratory Service and School of Pathology, Faculty of Health Sciences, University of the Witwatersrand, Johannesburg, South Africa; 27 Department of Psychiatry and Mental Health, Faculty of Health Sciences, University of Cape Town, Rondebosch, South Africa; 28 Department of Paediatrics and Child Health, and SAMRC Unit on Child and Adolescent Health, University of Cape Town, Cape Town, South Africa; 29 SAMRC Unit on Risk & Resilience in Mental Disorders, Department of Psychiatry, University of Cape Town, Cape Town, South Africa; 30 Neuroscience Institute, University of Cape Town, Cape Town, South Africa; 31 Broad Institute of MIT and Harvard, Cambridge, MA USA; 32 Department of Biomedicine—Human Genetics, Aarhus University, Aarhus, Denmark; 33 Lundbeck Foundation Initiative for Integrative Psychiatric Research, iPSYCH, Aarhus, Denmark; 34 Center for Genomics and Personalized Medicine, Aarhus, Denmark; 35 Department of Psychiatry, University of Cambridge, Cambridge, UK; 36 Department of Psychology, University of Cambridge, Cambridge, UK; 37 Research Department of Early Life Imaging, School of Biomedical Engineering and Imaging Sciences, King’s College London, London, UK; 38 MRC Centre for Neurodevelopmental Disorders, King’s College London, London, UK; 39 Division of Public Health & Epidemiology, School of Medical Sciences, University of Leicester, Leicester, UK; 40 Department of Psychiatry and Behavioral Sciences, UCSF Weill Institute for Neurosciences, University of California, San Francisco, San Francisco, CA USA; 41 Bioinformatics Research Centre, Aarhus University, Aarhus, Denmark; 42 Humboldt-Universität zu Berlin, Berlin, Germany; 43 German Center of Mental Health (DZPG), Berlin, Germany; 44 Institute for Human Genetics, Department of Psychiatry and Behavioral Sciences, University of California, San Francisco, San Francisco, CA USA; 45 Department of Clinical, Neuro- and Developmental Psychology, Vrije Universiteit Amsterdam, Amsterdam, the Netherlands; 46 Center for Applied and Translational Genomics, Mohammed Bin Rashid University of Medicine and Health Sciences, Dubai Health, Dubai, UAE; 47 GenomeArc Inc., Mississauga, Ontario Canada; 48 Centre for Precision Psychiatry, University of Oslo, Oslo, Norway; 49 Division of Mental Health and Addiction, Oslo University Hospital, Oslo, Norway; 50 KG Jebsen Centre for Neurodevelopmental Disorders, University of Oslo, Oslo, Norway; 51 Division of Psychological Medicine and Clinical Neurosciences & Centre for Neuropsychiatric Genetics and Genomics, Cardiff University, Cardiff, UK; 52 Centre for Applied Genomics and Genetics and Genomic Biology Program, Hospital for Sick Children, Toronto, Ontario Canada; 53 McLaughlin Centre and Department of Molecular Genetics, University of Toronto, Toronto, Ontario Canada; 54 Center for Genomic Medicine, Massachusetts General Hospital, Boston, MA USA; 55 Department of Psychiatry, Harvard Medical School, Boston, MA USA; 56 Stanley Center for Psychiatric Research, Broad Institute of MIT and Harvard, Cambridge, MA USA

**Keywords:** Behavioural genetics, Human behaviour

## Abstract

Early temperament, such as socio-emotional development and activity level, varies widely, yet its underlying biological associations are not understood. We identified genetic variation associated with infant and toddler temperament using genome-wide association meta-analyses. We studied parent-rated emotionality, activity, shyness and sociability (*n* = 43,963–72,663) in the second and third postnatal years and a cross-age average. Cross-age single nucleotide polymorphism heritabilities for emotionality, activity, shyness and sociability were 6.79% (95% confidence interval (CI), (4.71%, 8.87%)), 9.55% (95% CI, (7.04%, 12.06%)), 15.26% (95% CI, (12.24%, 18.28%)) and 3.42% (95% CI, (1.30%, 5.54%)), respectively. Ten genome-wide significant loci were discovered. Two loci colocalized with expression quantitative trait loci in the adult cortex: *RHEBL1* (posterior probability, 0.93; associated with activity) and *MR1* (posterior probability, 0.99; with emotionality). Genetic correlations were observed between early temperament and later outcomes, such as emotionality and adult neuroticism, activity and attention deficit/hyperactivity disorder (ADHD), sociability and autism, and shyness and adult extraversion. Multi-ancestry (*n* = 56,083–78,894) and European-ancestry analyses gave similar results. Infant and toddler temperament is associated with genetic variation and shows genetic continuity with later outcomes.

## Abstract (web summary)

A genome-wide association study of infant and toddler temperament in up to 78,000 infants found new genetic loci. Infant and toddler temperament showed significant single-nucleotide polymorphism heritability and genetic overlap with later life outcomes.

## Main

Infancy and toddlerhood comprise a fascinating and formative life stage in which children learn a range of fundamental skills including the abilities to move around and explore their surroundings, to express their emotions, and to socialize and build relationships with others. Here we define infancy and toddlerhood as the first three years of postnatal life. During this important period of skill acquisition, the human brain is growing more rapidly than at any other stage of the lifespan^1,2^. In addition, brain development is highly sensitive to environmental influences, and there is enhanced brain plasticity during this period. If certain environments are not present, then meaningful and often lifelong delays and deficits occur in neurodevelopment, behaviour and growth^3–7^.

It has become increasingly clear that for all complex behavioural traits, individual differences are explained by a combination of environmental and genetic factors^8,9^. Given the importance of the first three years for brain growth and skill acquisition, there is a considerable need for research on the role of genetic variation in explaining individual differences in core early behavioural domains including emotionality, activity, sociability and shyness^10^. These behavioural domains are referred to as infant and toddler temperament; they are observed in the first three years after birth and represent early developing personality traits^11–13^. A child’s temperament impacts their life widely; for example, it is likely to affect their learning experiences, the parenting they receive, their home environment, and their risk and resilience to life outcomes^14,15^. These temperament traits, such as socio-emotional well-being and activity level, are relevant to a wide range of sectors including medicine, social care, public health and education. For example, they are mentioned in clinical guidelines (such as NICE in the UK) and educational guidelines (such as the Ofsted Early Years educational framework in England)^16,17^.

Temperament has been defined by at least four prevailing theories^11,12,14^. Thomas and Chess^18^ partitioned temperament into nine dimensions representing the style in which behaviour is expressed—for example, the intensity of emotional reactions or the energy levels with which actions are performed. On the basis of the relative levels of these dimensions, this model categorized children into three types: ‘easy’, ‘difficult’ and ‘slow to warm’^14^. Meanwhile, the Rothbart approach to temperament focused on individual differences in reactivity and self-regulation, which were measured with three scales: negative affect, surgency and effortful control^19,20^. Goldsmith’s approach^21^ defined early temperament primarily in relation to emotions, focusing on the experience and expression of core emotions, such as anger or joy, using five scales^11,22,23^. Lastly, the EAS model from Buss and Plomin defined temperament as early developing personality and included scales of emotionality, activity, shyness and sociability. The EAS model has been included in developmental research for more than 50 years^24–26^ and continues to be used in contemporary research^25,27^, including in many large cohort studies^28–30^. The factor structure of these four scales has been replicated across multiple studies and countries^31–33^. In the EAS model, emotionality represents the threshold and intensity at which a child experiences negative emotions such as fear and anger. Activity involves the parameters of tempo and vigour; toddlers with high activity move around more and prefer being more active. Shyness refers to behaviour around strangers such as being inhibited, feeling distressed or tense and wanting to leave these social interactions. Finally, sociability refers to the tendency to want to interact with others.

Temperament has been shown to be related to later personality, neurodevelopmental conditions and mental health. Specifically, higher emotionality in infants and toddlers is associated with autism, ADHD and psychiatric disorders in childhood^34–37^ and with emotional disorders and psychopathology in adolescence^27,38^. Higher infant and toddler activity levels are associated with more childhood ADHD symptoms and also with diagnosis of ADHD^39–41^. Shyness in infants and toddlers is associated with autistic traits in late childhood and with difficulties with socio-emotional functioning and anxiety and depression symptoms in early adolescence^35,42^. Longitudinal studies have shown that temperament traits in infancy and toddlerhood explain variance in all major personality traits in later childhood^25,43,44^.

In terms of evidence of genetic factors, a recent large study employing twins, siblings and cousins reported heritabilities between 30% and 60% across the four EAS temperament traits of emotionality, activity, shyness and sociability, measured at ages 18 months and 36 months^25^. These results concur with a meta-analysis of infant (birth to two years) twin studies, which reported significant pooled twin heritability for a range of temperament and behavioural domains in infants, including 40% for emotional functions, 38% for basic interpersonal functions and 59% for psychomotor functions^8^. Additionally, polygenic score (PGS) analyses show genetic associations between infant and toddler temperament and neurodevelopmental conditions. For example, toddler emotionality was associated with polygenic liability for schizophrenia, and toddler activity was associated with polygenic liability for ADHD^45^. However, to date, there are very few genome-wide association studies (GWAS) that have sought to identify common genetic variation associated with infant and toddler behaviour^10^. Focusing on studies with an *n* > 5,000, currently there are GWAS of age at onset of walking^46^, preschool language^47,48^ and neurodevelopmental traits in three-year-olds^49^. Infant and toddler temperament has not been studied to date^10^.

We present a genome-wide association study of infant and toddler temperament, measured using parent report in multiple cohorts (total *n* = 43,963–72,663). The four EAS temperament traits of emotionality, activity, shyness and sociability were considered appropriate for use in GWAS given their replicated factor structure, their suitability for specific rating of behavioural traits in infants and toddlers, and the existing large family studies that confirm the significant heritability of these temperament traits. We investigated temperament measured in the second and third postnatal years and a mean across these two years^50^. First, we discovered that common genetic variation was associated with individual differences in infant and toddler temperament (cross-age single nucleotide polymorphism (SNP) heritabilities between 3.42% and 15.26%). Second, we identified ten independent genome-wide significant loci (SNPs), as well as multiple genes, that were significantly associated with infant and toddler temperament. Third, we found that two of the genome-wide significant loci colocalized with expression quantitative trait loci (eQTLs) in *RHEBL1* (posterior probability (PP_4_), 0.93) for activity and in *MR1* (PP_4_ = 0.99) for emotionality, both expressed in the adult human cortex. Finally, our results showed that significant genetic overlap exists between infant and toddler temperament and later outcomes, including between emotionality and adult neuroticism, between activity level and ADHD, between sociability and autism and between shyness and adult extraversion. We included both European-only ancestry analyses and multi-ancestry sample analyses. This study reveals that common genetic variation is significantly associated with individual differences in infant and toddler temperament and further demonstrates that this genetic variation is shared with the genetic pathways influencing later personality, neurodevelopmental conditions and mental health traits.

## Results

### Cohorts and sample sizes

We conducted GWAS of the temperament traits emotionality, activity, shyness and sociability in the second and third years after birth and as a cross-age average across the two years. We conducted GWAS in five European cohort samples and meta-analysed the results for each trait. The cohorts included in this study were the Norwegian Mother, Father and Child cohort study (MoBa) (*n* = 39,234–55,337); the Lifelines population-based birth cohort study from the North of the Netherlands (*n* = 3,435); the Avon Longitudinal Study of Parents and Children (ALSPAC) (*n* = 6,418–6,966) from South-West England; the Netherlands Twin Register (NTR) (*n* = 1,433); and the Twins Early Development Study (TEDS) (*n* = 4,673–5,686) from England and Wales. Cohort sample sizes for each temperament phenotype are provided in Supplementary Table 1.

### Genomic loci associated with infant and toddler temperament

We identified ten genome-wide significant (*P* < 5 × 10^−8^, two-sided) loci across the four cross-age and eight specific-age temperament GWAS analyses. Each significant locus contained one lead variant according to GCTA conditional and joint analysis (COJO)^51^. Table 1 lists these independent SNPs; regional plots for these loci are shown in Supplementary Figs. 1–4. Manhattan plots for emotionality, activity, shyness and sociability for the specific-age and cross-age analyses are shown in Supplementary Figs. 5–7. As per a reviewer suggestion, these loci were compared with the 15 independent loci cumulatively identified by the few existing GWAS of infant behavioural and neurodevelopmental traits^46–49,52^; none of the temperament-associated loci overlapped with any of the loci previously identified by these past GWAS.

**Table 1:** Independent SNPs from each of the genome-wide significant loci in each GWAS

Phenotype	rsID	Chr.	Position	A1	A2	A1 freq.	*β*	*β* s.e.	*P*	COJO *P*	*n*	Nearest gene	No. of prioritized genes
**Cross-age analyses**													
Emotionality	rs10797666	1	181018799	A	C	0.71	0.0383	0.007	4.35 × 10^−8^	4.50 × 10^−8^	50,377	*MR1*	12
	rs72921075	6	99734869	A	G	0.92	0.0664	0.0116	1.07 × 10^−8^	1.05 × 10^−8^	50,389	*FAXC*	12
	rs78155170	15	34104500	T	C	0.03	0.1158	0.021	3.44 × 10^−8^	3.54 × 10^−8^	45,440	*RYR3*	19
Activity	rs1064627	6	30698541	A	G	0.84	−0.0529	0.0096	3.88 × 10^−8^	3.62 × 10^−8^	39,274	*FLOT1*	94
	rs1744758	20	35737816	A	G	0.62	0.0375	0.0065	8.09 × 10^−9^	8.05 × 10^−9^	50,401	*MROH8*	33
Shyness	rs79263836	2	28646937	T	C	0.02	0.1373	0.025	3.86 × 10^−8^	4.01 × 10^−8^	45,617	*RP11-373D23.3*	34
	rs12173930	6	78186434	T	C	0.15	−0.0528	0.0092	1.10 × 10^−8^	9.62 × 10^−9^	45,574	*HTR1B*	9
**Specific-age analyses**													
Activity second year	rs3130985	6	31085356	T	C	0.14	0.056	0.0079	1.55 × 10^−12^	1.38 × 10^−12^	66,891	*PSORS1C1:CDSN*	163
Activity third year	rs1054442	12	49389320	A	C	0.63	0.0366	0.0063	5.21 × 10^−9^	*	55,043	*DDN:RP11-386G11.3*	58
Shyness second year	rs2416049	14	47801869	A	T	0.42	0.0317	0.0057	3.49 × 10^−8^	2.70 × 10^−8^	63,238	*MDGA2*	7

GWAS summary statistics are presented for each of the independent SNPs. Effect sizes (*β*) and their standard errors (*β* s.e.) refer to the effect of allele A1, and *P* values are from the meta-analyses performed in METAL. These are presented alongside the *P* value from the COJO analysis (COJO *P*), the nearest gene as mapped by FUMA on the basis of ANNOVAR annotations for the independent genomic loci, and the number of prioritized genes identified by FUMA positional mapping, eQTLs and chromatin mapping. Genomic positions are given using build GRCh37.

*Only one SNP reached genome-wide significance for activity in the third year, so COJO was not conducted.

FUMA analysis^53^ was used to functionally map each of the COJO independent SNPs. FUMA used positional mapping, associations with eQTLs and chromatin interactions to identify prioritized genes that mapped to the COJO independent SNPs for each trait. For the four cross-age analyses, FUMA identified 43 prioritized genes mapped to emotionality, 127 mapped to activity and 43 mapped to shyness. For the eight age-specific analyses, 163 genes were prioritized for activity in the second year, 7 for shyness in the second year and 58 for activity in the third year. The numbers of prioritized genes for the specific-age and cross-age analyses are shown in the rightmost column of Table 1. The list of prioritized genes per GWAS is given in Supplementary Table 2.

MAGMA^54^ identified genes associated with temperament by testing the association between each phenotype and the combined statistical effects of the SNPs mapped to genes. This approach provided additional power to identify associated genes in DNA regions where the GWAS failed to identify genome-wide significant SNPs. Significant associated genes from MAGMA are listed in Table 2. Two genes, *RBL1* and *TRIM26*, were significantly associated with cross-age activity. *IMMP2L* was significantly associated with cross-age shyness. Prior GWAS have found *IMMP2L* to be associated with schizophrenia^55^, smoking status^56^ and cognitive function^57^. Finally, for cross-age emotionality, no genes were significantly associated after Bonferroni correction. Sociability was not run in MAGMA due to having no genome-wide significant loci.

**Table 2:** Genes significantly associated with infant and toddler temperament

Gene	Chromosome	Start	Stop	*z* score	*P*
**Activity, second year**					
*MSH5*	6	31707725	31732622	6.40	7.66 × 10^−11^
*MSH5-SAPCD1*	6	31707797	31732628	6.40	7.66 × 10^−11^
*DPCR1*	6	30908749	30921998	5.62	9.52 × 10^−9^
*TRIM26*	6	30152232	30181204	5.38	3.69 × 10^−8^
*PBX2*	6	32152512	32157963	5.38	3.81 × 10^−8^
*SFTA2*	6	30899130	30899952	5.33	4.80 × 10^−8^
*GPSM3*	6	32158543	32163300	5.31	5.50 × 10^−8^
*PSORS1C2*	6	31105313	31107127	5.08	1.92 × 10^−7^
*TRIM31*	6	30070674	30080883	5.05	2.17 × 10^−7^
*CLIC1*	6	31698358	31707540	4.98	3.26 × 10^−7^
*LSM2*	6	31765173	31774761	4.95	3.62 × 10^−7^
*FAM150B*	2	279558	288851	4.80	8.12 × 10^−7^
*ACP1*	2	264140	278283	4.73	1.10 × 10^−6^
*HIST1H2AL*	6	27833034	27833606	4.70	1.33 × 10^−6^
**Activity, third year**					
*DDN*	12	49388932	49393092	5.13	1.46 × 10^−7^
*SUFU*	10	104263744	104393292	4.97	3.39 × 10^−7^
*ACTR1A*	10	104238986	104262482	4.78	8.60 × 10^−7^
*ASXL3*	18	31158579	31331156	4.57	2.47 × 10^−6^
**Activity, cross-age**					
*RBL1*	20	35624752	35724398	4.75	9.97 × 10^−7^
*TRIM26*	6	30152232	30181204	4.57	2.46 × 10^−6^
**Shyness, cross-age**					
*IMMP2L*	7	110303110	111202573	4.94	3.98 × 10^−7^

These genes were significantly associated (at a Bonferroni-corrected *P*-value threshold) with infant and toddler temperament according to two-sided tests performed by MAGMA. Bonferroni correction was used to adjust the *P*-value threshold for significance; the *P* values shown are uncorrected, and the corrected thresholds are *P* < 0.05/18,735 = 2.669 × 10^−6^ (activity, second year), *P* < 0.05/18,701 = 2.674 × 10^−6^ (activity, third year), *P* < 0.05/18,701 = 2.674 × 10^−6^ (activity, cross-age) and *P* < 0.05/18,629 = 2.684 × 10^−6^ (shyness, cross-age). The genomic positions of the genes (chromosome and start and stop positions) are given in build GRCh37.

For activity in the second year, MAGMA identified 14 significantly associated genes (Table 2). For activity in the third year, MAGMA identified four significantly associated genes: *DDN*, *SUFU*, *ACTR1A* and *ASXL3*. *SUFU* has been associated with multiple phenotypes, including educational attainment^58^ and waist-to-hip ratio^59^. *DDN* has been associated with educational attainment^56^ and intelligence^60^. Finally, *ASXL3* has been found to be associated with pulmonary function^61^ and body mass index (BMI)^62^]. Most significantly associated genes were age-specific (Table 2 and Supplementary Table 3).

For a full list of phenotypes previously found to be associated with the genes that were associated with temperament in our study, see Supplementary Table 4; these were identified using GWAScatalog, through MAGMA. Supplementary Table 5 lists the overlap between the significant SNPs associated with infant and toddler temperament and significant SNPs found in previous GWAS studies listed in GWAScatalog, identified through FUMA. For the FUMA-identified genes from activity in the second year, 24 genes (Bonferroni-adjusted *P* value for enrichment, *P*_adj._ = 0.041) were upregulated in differentially expressed gene sets in the whole blood tissue type (Supplementary Fig. 8a). This was out of 54 specific tissue types analysed. There was also significant downregulation of the differentially expressed genes in breast mammary tissue among the FUMA-identified genes for activity in the third year (*P*_adj._ = 0.033; Supplementary Fig. 8b). There was significantly enriched expression of the genes associated with cross-age shyness in the hypothalamus (standardized *β* = 0.047; 95% confidence interval (CI), (0.032, 0.062); *P* = 1.55 × 10^−4^) and pituitary gland (std. *β* = 0.052; 95% CI, (0.036, 0.068); *P* = 4.35 × 10^−4^), as identified by MAGMA GTEx tissue expression analysis using a Bonferroni-corrected *P*-value threshold of <9 × 10^−4^ (Supplementary Fig. 9). There was no evidence of significant enrichment of the genes associated with other temperament traits in any specific tissues from MAGMA GTEx tissue expression analysis (Supplementary Table 6). MAGMA gene-property analysis showed an absence of evidence for enrichment of the MAGMA genes in the brain across 11 BrainSpan general developmental stages from prenatal to middle adulthood (Supplementary Table 7).

Genes associated with temperament were significantly enriched for genes found to be associated with autism. Specifically, of the four temperament-associated genes present in the Genomics England PanelApp database (v. 0.36)^63^, 50% were associated with autism, which is over ten times the proportion of autism-associated genes in the panel (734 of a total of 19,950; 3.68%), indicating significant enrichment of autism genes in those associated with temperament (Fisher’s exact test; odds ratio, 26.16; 95% CI, (1.89, 359.60); *P* = 0.008; two-sided). These temperament genes were *ASXL3* and *IMMP2L* (Supplementary Table 8).

### Colocalization with gene expression in the human cortex

Using colocalization analyses in coloc^64^, we tested whether the genome-wide significant loci identified above shared causal variants with eQTLs specifically in the human cortex. We prioritized genes within ±1 Mb of the ten genome-wide significant loci (*P* < 5 × 10^−8^) listed in Table 1 as well as the genes significantly associated with the phenotype in the MAGMA gene-level analysis across all GWAS (Table 2). We then examined whether SNPs associated with the expression of these genes (eQTLs) were also significant in the GWAS, using an independent dataset of post-mortem bulk RNA-seq from 1,433 samples of the human adult cortex^65^.

We identified two eQTLs that colocalized with significant lead SNPs from our GWAS: one for activity in the third year and one for cross-age emotionality. First, we identified significant colocalization between eQTLs for *RHEBL1* on chromosome 12 and the genome-wide significant locus for activity in the third year (PP_4_ = 0.93) (Supplementary Fig. 10). The alternate allele of the COJO lead SNP at this locus, rs1054442 (chr. 12: 49389320:A:C; GRCh37), was associated with decreased activity levels (*β* = −0.036, *β* s.e. = 0.006, *P* = 5.21 × 10^−9^) and increased expression of *RHEBL1* (ref. ^65^) (*β* = 0.314, *P* = 1.60 × 10^−17^). This variant was also a lead SNP in GWAS for both educational attainment (alternate allele *β* = 0.014, *β* s.e. = 0.002, *P* = 8.42 × 10^−16^)^66^ and cognitive performance (alternate allele *β* = 0.021, *β* s.e. = 0.003, *P* = 2.52 × 10^−14^)^67^, with the alternate allele associated with more years of schooling and enhanced cognitive performance (Supplementary Fig. 11). *RHEBL1* encodes the Ras homologue enriched in brain-like protein 1 (hereafter RhebL1), which is a positive regulator of NF-κB-mediated gene transcription^68^. *RHEBL1* is expressed in the brain^68^, and RhebL1 is a modulator of the mTOR signalling pathway^69,70^.

We also observed significant colocalization between eQTLs for *MR1* on chromosome 1 and the genome-wide significant locus for cross-age emotionality (PP_4_ = 0.99) (Supplementary Fig. 12). The alternate allele of the COJO lead SNP at this locus, rs10797666 (chr. 1: 181018799:A:C; GRCh37), is associated with increased expression of *MR1* (ref. ^65^) (*β* = 0.453, *P* = 3.40 × 10^−29^) and decreased emotionality (*β* = −0.038, *β* s.e. = 0.007, *P* = 4.35 × 10^−8^). *MR1* encodes the Major Histocompatibility Complex (MHC) Class I-Related protein 1, a non-classical MHC molecule that presents non-peptide antigens, such as metabolites from microbial organisms, to a specialized subset of T cells called Mucosal-Associated Invariant T cells^71^.

### Common genetic architecture of infant and toddler temperament traits

The point estimates of SNP-based heritability (*h*^2^_SNP_) were consistently the highest for shyness and the lowest for sociability. For cross-age temperament, the *h*^2^_SNP_ estimate was 6.79% for emotionality (s.e. = 1.06%; 95% CI, (4.71%, 8.87%)), 9.55% for activity (s.e. = 1.28%; 95% CI, (7.04%, 12.06%)), 15.26% for shyness (s.e. = 1.54%; 95% CI, (12.24%, 18.28%)) and 3.42% for sociability (s.e. = 1.08%; 95% CI, (1.30%, 5.54%)). This rank-order pattern of *h*^2^_SNP_ point estimates was also seen in the largest cohort, MoBa, for which the highest cross-age *h*^2^_SNP_ estimate was for shyness (*h*^2^_SNP_ = 15.69%; s.e. = 1.67%; 95% CI, (12.42%, 18.96%)), then activity (*h*^2^_SNP_ = 10.34%; s.e. = 1.63%; 95% CI, (7.15%, 13.53%)), then emotionality (*h*^2^_SNP_ = 8.2%; s.e. = 1.4%; 95% CI, (5.46%, 10.94%)) and lowest for sociability (*h*^2^_SNP_ = 3.32%; s.e. = 1.2%; 95% CI, (0.97%, 5.67%)). All *h*^2^_SNP_ estimates for the GWAS meta-analyses and for the GWAS in MoBa are provided in Supplementary Table 9; see also Supplementary Note 1.

In the GWAS meta-analyses, we observed minor inflation of the test statistics (as quantified by the *λ* estimate from linkage disequilibrium (LD) score regression (LDSC)) for shyness in the second-year (*λ*_GC_ = 1.108), third-year (*λ*_GC_ = 1.14) and cross-age (*λ*_GC_ = 1.127) analyses and for activity in the second-year (*λ*_GC_ = 1.111) analyses. However, the attenuation ratio values for these analyses indicated that there was minimal inflation of the test statistics due to factors other than polygenicity (Supplementary Table 9)^72,73^.

Heterogeneity analyses revealed an absence of evidence for genome-wide significant (*P* < 5 × 10^−8^) heterogeneity for any activity or sociability GWAS. Significant heterogeneity was found for one SNP in the cross-age shyness meta-analysis (rs71456572; chr. 12: 93121658; GRCh37; *χ*^2^(1) = 30.34; *P* = 3.63 × 10^−8^; *I*^2^ = 96.7%; 95% CI, (83.44%, 100%)) and one SNP in one of the second-year emotionality meta-analyses (rs33887; chr. 5: 123751831; GRCh37; *χ*^2^(3) = 38.43; *P* = 3.30 × 10^−8^; *I*^2^ = 92.2%; 95% CI, (94.57%, 99.90%)). Neither of these SNPs reached genome-wide significance in their respective meta-analyses (Supplementary Note 2).

Genetic correlations (*r*_g_) between sex-stratified GWAS meta-analyses for all phenotypes were all high and significantly different from zero (*P* < 0.01, two-sided; Supplementary Table 10) except sociability in the second and third years and emotionality in the second year, for which the genetic correlations were high but did not reach significance. As requested by a reviewer, the *β* values for the genome-wide significant SNPs listed in Table 1 were compared for the male-only and female-only analyses, which revealed an absence of evidence for a significant difference between the effect sizes of these SNPs in the sex-stratified results, apart from rs79263836 in the cross-age shyness analyses, for which there was a significant difference (*P* = 0.016, two-sided) (Supplementary Table 11).

### Inter-cohort genetic correlations

We calculated the inter-cohort genetic correlations for each EAS temperament trait between MoBa and other cohorts large enough for bivariate LDSC^74^—namely, ALSPAC and TEDS. The mean inter-cohort genetic correlation was 0.79. All inter-cohort genetic correlations were above 0.5 except the MoBa–ALSPAC genetic correlation for cross-age emotionality, which was low with a large 95% CI (*r*_g_ = 0.377; s.e. = 0.581; *P* = 0.517; 95% CI, (−0.762, 1.516)); however, most genetic correlations between cohorts did not reach significance (*P* < 0.05). Not all genetic correlations could be estimated due to low power in some analyses. Inter-cohort genetic correlations for MoBa, ALSPAC and TEDS are presented in Supplementary Table 12.

### Genetic correlations

**Fig. 1:**
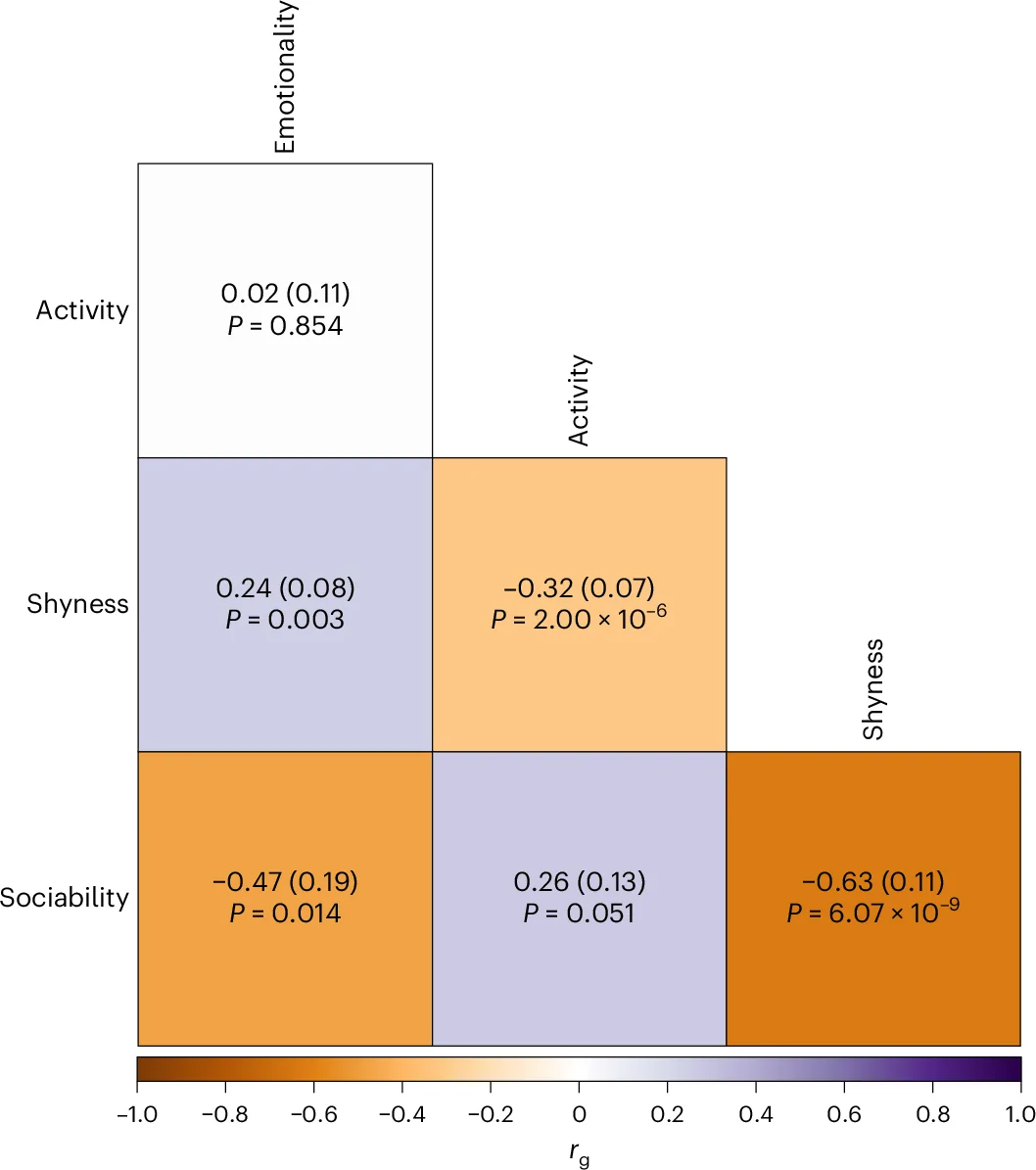
Genetic correlations between each of the four cross-age temperament traits. Genetic correlations between cross-age emotionality (*n* = 50,426), activity (*n* = 50,453), shyness (*n* = 45,656) and sociability (*n* = 43,963) are shown with standard errors in parentheses and uncorrected *P* values (two-tailed) below. LDSC genetic correlations are colour coded on a scale from dark orange (*r*_g_ = −1) to dark purple (*r*_g_ = 1) with lighter orange and purple indicating lower magnitudes of genetic correlation, fading to white at a genetic correlation of 0.

Figure 1 shows the genetic correlations between the four cross-age temperament traits (two-sided). Between-temperament genetic correlations revealed a negative genetic correlation between shyness and sociability (*r*_g_ = −0.625; s.e. = 0.107; *P* = 6.07 × 10^−9^; 95% CI, (−0.835, −0.414)) and negative genetic correlations between emotionality and sociability (*r*_g_ = −0.472; s.e. = 0.191; *P* = 0.014; 95% CI, (−0.847, −0.096)) and between activity and shyness (*r*_g_ = −0.316; s.e. = 0.067; *P* = 2.00 × 10^−6^; 95% CI, (−0.446, −0.186)). There was a positive genetic correlation between emotionality and shyness (*r*_g_ = 0.244; s.e. = 0.082; *P* = 0.003; 95% CI, (0.084, 0.404)). The results were similar for the specific-age, between-temperament genetic correlations (Supplementary Fig. 13 and Supplementary Table 13). Within-temperament genetic correlations between the second and third postnatal years were all high, positive and significant, ranging from *r*_g_ = 0.845 to 0.983 (Supplementary Table 14).

**Fig. 2:**
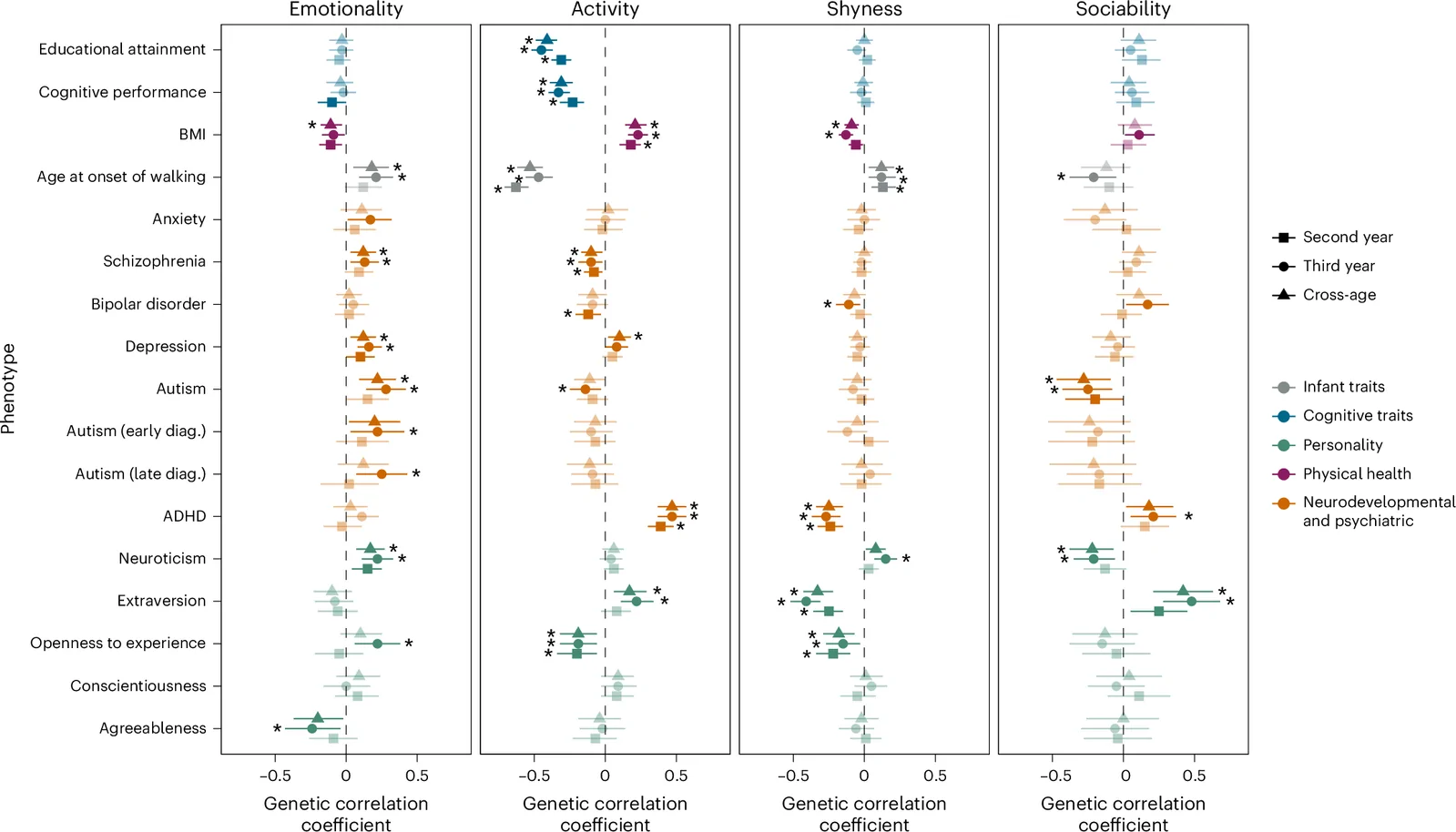
Genetic correlations between temperament traits and other cognitive, personality, physical health, neurodevelopmental and psychiatric phenotypes. Genetic correlations between infant and toddler temperament traits and other phenotypes: educational attainment, cognitive performance, BMI, age at onset of walking, lifetime anxiety disorder (anxiety), depression, autism, autism diagnosed before age 11 and autism diagnosed after age 10 (autism (early diag.) and autism (late diag.), respectively), ADHD, schizophrenia, bipolar disorder (types 1 and 2), and adulthood neuroticism, extroversion, openness to experience, conscientiousness and agreeableness. The points represent LDSC genetic correlation coefficients, and the bars represent the 95% CIs. Solid points and bars represent coefficients for which the uncorrected *P* value is <0.05 (two-sided). Point shapes represent whether the analyses were conducted for cross-age (triangle), second-year (square) or third-year (circle) temperament. The maximum GWAS sample sizes for each of the phenotypes included in these genetic correlation analyses were as follows: for emotionality, cross-age *n* = 50,426, second-year *n* = 69,194, third-year *n* = 55,262; for activity, cross-age *n* = 50,453, second-year *n* = 72,663, third-year *n* = 55,280; for shyness, cross-age *n* = 45,656, second-year *n* = 63,663, third-year *n* = 55,252; for sociability, cross-age *n* = 43,963, second-year *n* = 62,230, third-year *n* = 55,240; for educational attainment, *n* = 765,283; for cognitive performance, *n* = 269,867; for BMI, *n* = 795,640; for age at onset of walking, *n* = 70,560; for anxiety, *n*_cases_ = 25,453, *n*_controls_ = 58,113; for schizophrenia, *n*_cases_ = 67,390, *n*_controls_ = 94,015; for bipolar disorder, *n*_cases_ = 27,196, *n*_controls_ = 43,792; for depression, *n*_cases_ = 412,303, *n*_controls_ = 1,588,397; for autism, *n*_cases_ = 18,382, *n*_controls_ = 27,969; for autism (early diag.), *n*_cases_ = 9,500, *n*_controls_ = 36,667; for autism (late diag.), *n*_cases_ = 9,231, *n*_controls_ = 36,667; for ADHD, *n*_cases_ = 38,691, *n*_controls_ = 186,843; for neuroticism, *n* = 682,688; for extraversion, *n* = 298,772; for openness to experience, *n* = 237,390; for conscientiousness, *n* = 252,253; and for agreeableness, *n* = 252,749. A Benjamini–Hochberg procedure was applied within each group of genetic correlations (that is, separately per temperament trait and age range—cross-age/second-year/third-year); those that remained significant at an FDR of 5% are indicated by asterisks. See Supplementary Table 15 for the full genetic correlation estimates.

Figure 2 shows the genetic correlations between the four EAS temperament traits and 17 physical health, cognitive, neurodevelopmental, psychiatric and personality phenotypes. The results for genetic correlations with cross-age temperament traits are reported and discussed below. All results for the genetic correlations between infant and toddler temperament and other phenotypes, including specific-age results, are shown in Fig. 2 and Supplementary Table 15. Genetic correlations are reported below where the false-discovery-rate-adjusted *P* values (*P*_FDR_) were <0.05 (two-sided; adjusted using the Benjamini–Hochberg procedure).

For cognitive phenotypes, negative genetic correlations were revealed between activity and educational attainment (*r*_g_ = −0.413; s.e. = 0.038; *P* = 1.65 × 10^−27^; 95% CI, (−0.488, −0.339); *P*_FDR_ = 1.40 × 10^−26^) and cognitive performance (*r*_g_ = −0.312; s.e. = 0.041; *P* = 4.81 × 10^−14^; 95% CI, (−0.393, −0.231); *P*_FDR_ = 2.04 × 10^−13^). These indicated a genetic association between lower activity levels in infancy and toddlerhood and higher educational attainment and higher cognitive performance.

For all temperament traits except sociability, there were significant genetic correlations with BMI. These genetic correlations were negative with emotionality (*r*_g_ = −0.106; s.e. = 0.037; *P* = 0.005; 95% CI, (−0.179, −0.032); *P*_FDR_ = 0.020) and shyness (*r*_g_ = −0.093; s.e. = 0.025; *P* = 2 × 10^−4^; 95% CI, (−0.142, −0.044); *P*_FDR_ = 0.001) and positive with activity (*r*_g_ = 0.214; s.e. = 0.038; *P* = 1.15 × 10^−8^; 95% CI, (0.140, 0.287); *P*_FDR_ = 3.92 × 10^−8^).

Age at onset of walking (measured in months) had a negative genetic correlation with activity (*r*_g_ = −0.531; s.e. = 0.048; *P* = 6.41 × 10^−29^; 95% CI, (−0.625, −0.438); *P*_FDR_ = 1.09 × 10^−27^) and positive genetic correlations with shyness (*r*_g_ = 0.117; s.e. = 0.047; *P* = 0.012; 95% CI, (0.026, 0.208); *P*_FDR_ = 0.040) and emotionality (*r*_g_ = 0.176; s.e. = 0.062; *P* = 0.005; 95% CI, (0.054, 0.297); *P*_FDR_ = 0.020).

For psychiatric disorders, we found genetic correlations between infant and toddler temperament and schizophrenia and depression. There was a negative genetic correlation between activity and schizophrenia (*r*_g_ = −0.096; s.e. = 0.040; *P* = 0.016; 95% CI, (−0.173, −0.018); *P*_FDR_ = 0.029) and a positive genetic correlation between emotionality and schizophrenia (*r*_g_ = 0.119; s.e. = 0.048; *P* = 0.013; 95% CI, (0.025, 0.213); *P*_FDR_ = 0.037). For depression, there was a positive genetic correlation with emotionality (*r*_g_ = 0.118; s.e. = 0.045; *P* = 0.009; 95% CI, (0.030, 0.207); *P*_FDR_ = 0.029) and a positive genetic correlation with activity (*r*_g_ = 0.099; s.e. = 0.040; *P* = 0.014; 95% CI, (0.020, 0.178); *P*_FDR_ = 0.029).

In terms of neurodevelopmental conditions, the genetic correlation was negative between sociability and autism (*r*_g_ = −0.281; s.e. = 0.096; *P* = 0.003; 95% CI, (−0.470, −0.093); *P*_FDR_ = 0.024) and positive between emotionality and autism (*r*_g_ = 0.217; s.e. = 0.066; *P* = 0.001; 95% CI, (0.088, 0.346); *P*_FDR_ = 0.010). ADHD had a positive genetic correlation with activity (*r*_g_ = 0.468; s.e. = 0.051; *P* = 8.39 × 10^−20^; 95% CI, (0.367, 0.569); *P*_FDR_ = 4.76 × 10^−19^) and a negative genetic correlation with shyness (*r*_g_ = −0.249; s.e. = 0.049; *P* = 3.13 × 10^−7^; 95% CI, (−0.344, −0.153); *P*_FDR_ = 2.66 × 10^−6^).

We found significant genetic correlations between temperament and personality traits in adulthood, specifically neuroticism, extraversion and openness to experience. Neuroticism had a positive genetic correlation with emotionality (*r*_g_ = 0.169; s.e. = 0.052; *P* = 0.001; 95% CI, (0.067, 0.271); *P*_FDR_ = 0.010) and a negative genetic correlation with sociability (*r*_g_ = −0.223; s.e. = 0.078; *P* = 0.004; 95% CI, (−0.376, −0.070); *P*_FDR_ = 0.024). Extraversion had positive genetic correlations with sociability (*r*_g_ = 0.420; s.e. = 0.107; *P* = 8.97 × 10^−5^; 95% CI, (0.210, 0.630); *P*_FDR_ = 0.002) and activity (*r*_g_ = 0.174; s.e. = 0.059; *P* = 0.003; 95% CI, (0.059, 0.289); *P*_FDR_ = 0.009) and a negative genetic correlation with shyness (*r*_g_ = −0.325; s.e. = 0.053; *P* = 5.90 × 10^−10^; 95% CI, (−0.428, −0.222); *P*_FDR_ = 1.00 × 10^−8^). We found negative genetic correlations between openness to experience and both shyness (*r*_g_ = −0.181; s.e. = 0.058; *P* = 0.002; 95% CI, (−0.293, −0.068); *P*_FDR_ = 0.007) and activity (*r*_g_ = −0.188; s.e. = 0.067; *P* = 0.005; 95% CI, (−0.319, −0.056); *P*_FDR_ = 0.013).

Pairs of phenotypes for which the genetic correlations were significant at an FDR *α* threshold of 0.05 were investigated further using causal mixture modelling (MiXeR)^75,76^. These results can be found in Supplementary Note 3 and Supplementary Table 16. Cross-age shyness and second-year shyness were sufficiently powered to conduct MiXeR analyses. Univariate MiXeR analyses found the polygenicity of cross-age shyness to be 8.5k SNPs (s.e. = 1.5k SNPs) and of second-year shyness to be 6.5k SNPs (s.e. = 0.9k SNPs). Bivariate models revealed substantial polygenic overlap between cross-age shyness and age at onset of walking (94.78%) and ADHD (86.49%) and between second-year shyness and extraversion (93.46%) and ADHD (96.54%). Polygenic overlap between cross-age shyness and BMI was moderate (38.8%). The fraction of SNPs with concordant directions ranged from 36.09% to 55.99% with a median of 40.53%.

### PGS

**Fig. 3:**
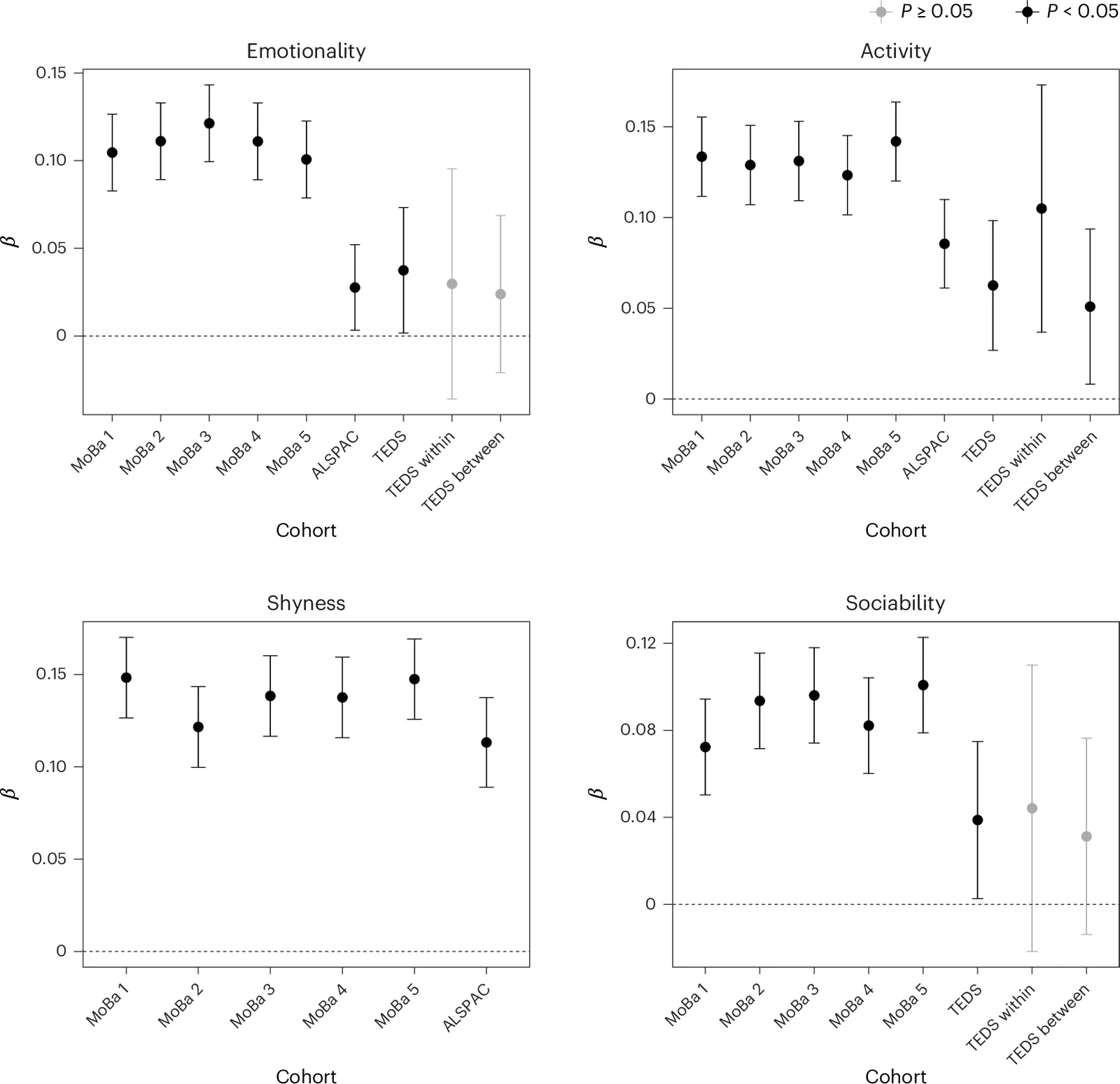
Within-trait prediction of PGS in independent target cohorts using a leave-one-out approach. PGS standardized *β* coefficients of linear regression models testing for associations between the EAS temperament traits and their PGS. The target samples were ALSPAC (for emotionality, *n* = 6,423; for activity, *n* = 6,425; for shyness, *n* = 6,418), TEDS (for emotionality, *n* = 2,989; for activity, *n* = 2,981; for sociability, *n* = 2,928), MoBa 1–5 (for emotionality, samples 1–4, *n* = 7,847, and sample 5, *n* = 7,846; for activity, samples 1–4, *n* = 7,855, and sample 5, *n* = 7,854; for shyness, samples 1 and 2, *n* = 7,847, and samples 3–5, *n* = 7,848; for sociability, samples 1–5, *n* = 7,858) and TEDS within/between (for emotionality, *n*_pairs_ = 1,509; for activity, *n*_pairs_ = 1,498; for sociability, *n*_pairs_ = 1,467; TEDS within- and between-sibling-pair analyses). The points represent standardized *β* coefficients ± 95% CIs (error bars). The points and error bars are black when the uncorrected *P* value (two-tailed) for the *β* coefficient was *P* < 0.05 and grey for *P* ≥ 0.05.

A leave-one-out^77^ PGS analysis was conducted to estimate the predictive ability of the PGS for infant and toddler temperament traits in independent samples. PGS were calculated using meta-analyses of the cohorts, leaving out the sample in which the PGS was computed. The standardized *β* coefficients for each of these PGS models for cross-age temperament are depicted in Fig. 3. In cross-age analyses, the PGS were significantly associated with each temperament trait analysed in ALSPAC (emotionality: *β* = 0.028; 95% CI, (0.003, 0.052); *P* = 0.027; *R*^2^ = 6.1 × 10^−4^; activity: *β* = 0.086; 95% CI, (0.061, 0.11); *P* = 6.33 × 10^−12^; *R*^2^ = 7.2 × 10^−3^; shyness: *β* = 0.113; 95% CI, (0.089, 0.137); *P* < 2 × 10^−16^; *R*^2^ = 0.013) and with each temperament trait analysed in TEDS (emotionality: *β* = 0.037; 95% CI, (0.002, 0.073); *P* = 0.041; *R*^2^ = 1.07 × 10^−3^; activity: *β* = 0.063; 95% CI, (0.027, 0.098); *P* = 6.07 × 10^−4^; *R*^2^ = 3.6 × 10^−3^; sociability: *β* = 0.039; 95% CI, (0.003, 0.075); *P* = 0.035; *R*^2^ = 1.17 × 10^−3^). Considering that the MoBa sample comprised the majority of each meta-analysed sample size, this sample could not be left out in its entirety. As done elsewhere^46^, we applied a fivefold cross-validation PGS analysis in MoBa, which produced a mean variance explained of *R*^2^ = 0.012 (s.e. = 7.8 × 10^−4^) for emotionality, *R*^2^ = 0.017 (s.e. = 8.3 × 10^−4^) for activity, *R*^2^ = 0.019 (s.e. = 1.3 × 10^−3^) for shyness and *R*^2^ = 0.0079 (s.e. = 9.0 × 10^−4^) for sociability. The results for specific ages were similar (Supplementary Figs. 14 and 15 and Supplementary Table 17).

To investigate whether the genetic associations identified by these GWAS analyses might be confounded by indirect genetic effects, we used a within- and between-sibling-pair PGS analysis^78^ in the twin cohort studies, TEDS and NTR. The results presented in Table 3 are for cross-age analyses, using the TEDS cohort; the results for specific-age analyses (which include the NTR cohort as well as TEDS) are presented in Supplementary Table 18 and Supplementary Figs. 14 and 15. PGS weights were computed using a meta-analysis of the cohorts from the main analysis, excluding TEDS. For the three temperament traits, we found no evidence of a significant difference between the within- and between-family standardized regression coefficients from the PGS model (Table 3).

**Table 3:** Within- and between-family PGS *β* estimates and *χ*^2^ tests for significant differences between them

Temperament trait	Within-family PGS *β* coefficient	Between-family PGS *β* coefficient	*χ*^2^
Emotionality	0.03	0.02	*χ*^2^(1, *n* = 3,018) = 0.02, *P* = 0.89, two-tailed
Activity	0.10	0.05	*χ*^2^(1, *n* = 2,996) = 1.73, *P* = 0.19, two-tailed
Sociability	0.04	0.03	*χ*^2^(1, *n* = 2,934) = 0.10, *P* = 0.75, two-tailed

*β* coefficients for the prediction of cross-age temperament traits using within-family and between-family PGS in the TEDS target sample. *χ*^2^ test statistics assess the difference between the within- and between-sibling-pair PGS *β* coefficients (two-tailed). Shyness is not included here because the TEDS sample did not have phenotypic data for both ages.

In other words, we did not observe evidence from these analyses that the genetic signals are biased by indirect genetic associations; nevertheless, these results should be interpreted with caution considering the overall predictive power of the PGS.

### Multi-ancestry GWAS meta-analysis of infant and toddler temperament

**Fig. 4:**
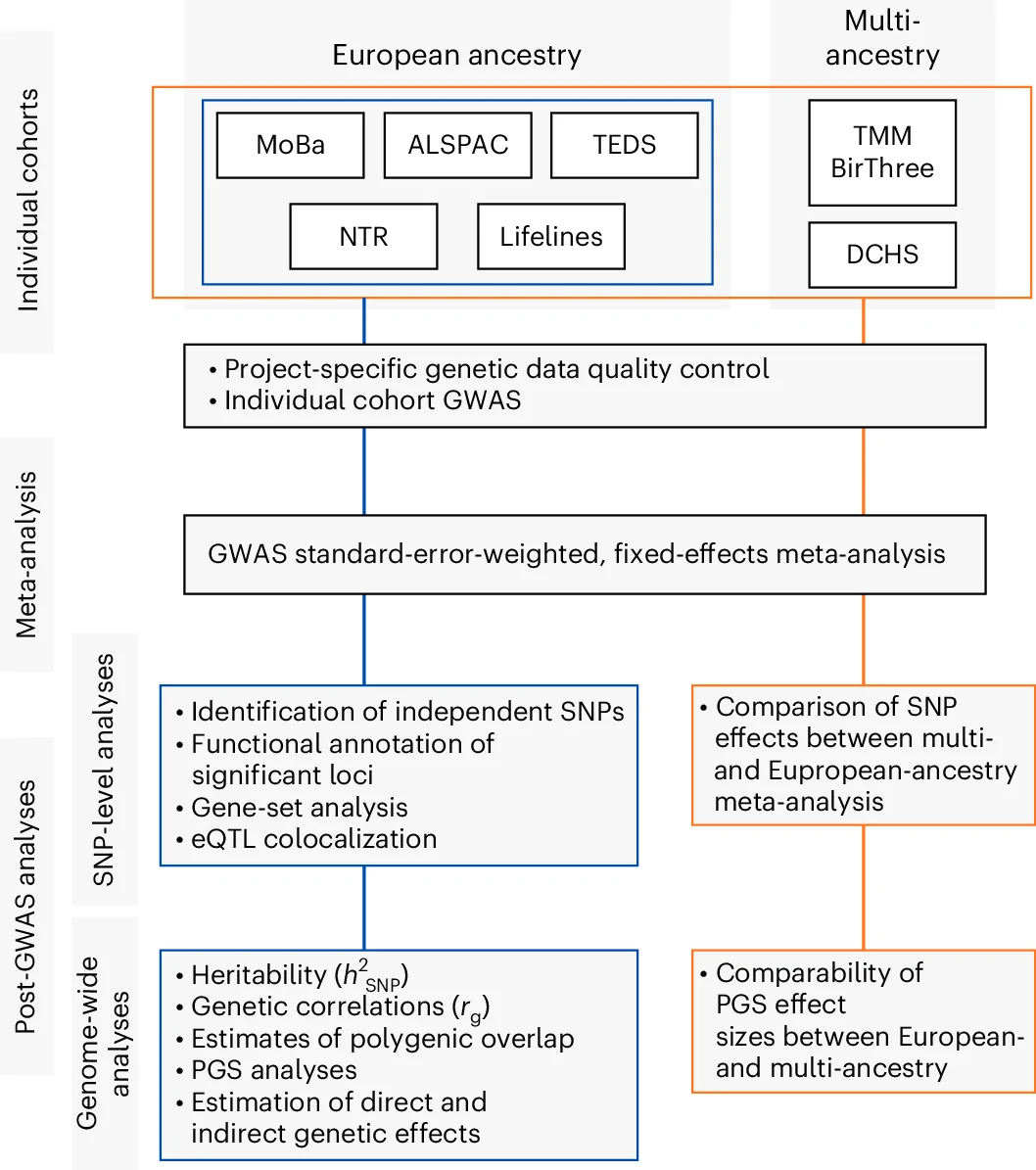
Overview of methods for infant and toddler temperament GWAS and downstream analyses. The blue boxes outline the analyses conducted in the European-ancestry meta-analysis. The orange boxes outline the analyses conducted in the multi-ancestry meta-analysis. The black boxes indicate methods used in individual cohorts and for both the European- and multi-ancestry meta-analyses.

As per the study preregistration, we sought to identify cohorts with diverse ancestries that included infant and toddler temperament measures (Fig. 4). We identified only two cohorts: the Tohoku Medical Megabank (TMM) Birth and Three-Generation Cohort Study (TMM BirThree; *n* = 5,618–5,653) and the Drakenstein Child Health Study (DCHS; *n* = 595–843); the sample sizes for specific ages and temperament traits in these two cohorts can be found in Supplementary Table 19. TMM BirThree is a cohort in Japan with participants of Japanese genetic ancestry, and the DCHS is in South Africa with participants of Black African and mixed ancestry. These cohorts measured temperament using the Child Behaviour Checklist (CBCL)^79^; TMM BirThree measured this in the second year, and the DCHS measured this in both the second and third years.

We meta-analysed these cohorts with the European ancestry cohorts described above using the same methods for meta-analysis: standard-error weighted fixed-effects meta-analysis in METAL^80^, entering the cohorts in order of sample size (largest first) and filtering out SNPs that did not meet a minimum sample size threshold (Methods). The meta-analysed sample sizes can be found in Supplementary Table 19.

Of the three genome-wide significant loci from the specific-age European meta-analyses, two were genome-wide significant in the multi-ancestry GWAS meta-analyses; however, one of these (rs3130985, activity, second-year) was not present in the individual summary statistics for the DCHS or TMM, so we concluded that one of these SNPs (rs1054442, activity, third year) is considered genome-wide significant in these multi-ancestry analyses (Table 4). The heterogeneity analyses revealed an absence of evidence for genome-wide significant heterogeneity (*P* < 5 × 10^−8^) for these three SNPs that reached genome-wide significance in the meta-analyses (Supplementary Table 20). Manhattan plots for these GWAS analyses are shown in Supplementary Figs. 16 and 17.

**Table 4:** Independent SNPs from each of the genome-wide significant loci in the single-age multi-ancestry GWAS meta-analyses

Temperament	rsID	Chr.	Position	A1	A2	A1 freq.	*β*	*β* s.e.	*P*	*n*	Direction
Activity second year	rs3130985	6	31085356	T	C	0.1387	0.056	0.0079	1.55 × 10^−12^	66,891	++??+−?
Activity third year	rs1054442	12	49389320	A	C	0.6325	0.0367	0.0062	3.55 × 10^−9^	55,794	++++
Shyness second year	rs2416049	14	47801869	A	T	0.4368	0.0298	0.0056	9.83 × 10^−8^	69,413	+−++

GWAS summary statistics from the multi-ancestry GWAS meta-analysis are presented for each of the independent SNPs from the European ancestry meta-analysis (Table 1), for comparison of effects.

Effect sizes (*β*) and their standard errors (*β* s.e.) refer to the effect of allele A1, and *P* values are from the multi-ancestry meta-analyses performed in METAL. The independent SNP for activity in the second year (rs3130985) was not present in the TMM or DCHS GWAS, so the summary statistics for this SNP are the same as for the meta-analysis of only European samples. Genomic positions for SNPs are given for genome build GRCh37.

We conducted PGS analyses using a leave-one-out design (as above for European cohorts), using the multi-ancestry GWAS meta-analysis with the target sample excluded as the training data for PGS weights, to predict the temperament phenotypes in ALSPAC and TEDS. We conducted these PGS analyses for temperament in the second year as this age included both of the diverse-ancestry cohorts. The results indicated that the differences in *β* coefficients when using all cohorts versus using only European cohorts in the training data were minor (Supplementary Fig. 18 and Supplementary Table 21).

Due to the low sample size for cross-age analyses in the DCHS and the fact that TMM BirThree did not include measures at both ages, we did not conduct multi-ancestry meta-analyses for cross-age temperament.

## Discussion

Infants and toddlers vary widely in how they behave, express their emotions, socialize and build relationships with others. These universal temperamental traits can broadly impact children’s lives, affecting their education (for example, capacity to sit still), the parenting they receive (for example, capacity to bond with others), their home environment (for example, preference for social company) and their risk and resilience throughout life (for example, capacity for regulating their emotions). The present study reveals that these individual differences in parent-rated infant and toddler temperament are heritable and they share some of their genetic associations with one another and with later life outcomes. Furthermore, we identified ten independent genome-wide significant loci associated with infant and toddler temperament. Interrogation of these loci revealed two eQTLs for *RHEBL1* and *MR1* in the adult human cortex, offering insight into the underlying biological associations of infant and toddler temperament and providing candidates for further functional follow-up. This gene discovery study of infant and toddler temperament reveals the extent and type of genome-wide common genetic variation involved in these formative early behaviours and confirms their biological associations with other infant traits and later neurodevelopmental and clinical outcomes. We present five main conclusions from this study.

First, our results establish that individual differences in infant and toddler temperament are heritable and associated with common genetic variation. All four aspects of temperament showed significant SNP heritability, being highest for shyness (15%), lowest for sociability (3%) and intermediate for activity (10%) and emotionality (7%). We identified ten independent genome-wide significant loci from the GWAS. The PGS derived from our GWAS, in which common genetic variation is summed together (weighted by effect size), showed associations with variance in these temperament traits in independent samples.

Second, our study shows that the associations of genetic variants of infant and toddler temperament with related behaviours persist throughout life. We discovered overlap in common genetic variation between infant and toddler activity and diagnosed ADHD, which aligns with observational studies reporting that infant and toddler activity associates with later ADHD^36,39,40^. We found genetic overlap between infant and toddler sociability and autism, with genetic variation associated with more infant and toddler sociability being associated with less likelihood for autism. This is consistent with the diagnostic criteria, since autism is a condition diagnosed on the basis of behaviours that include differences in socio-communication, typically starting in early life^81^. Furthermore, our results reveal that infant and toddler temperament is associated genetically with adult personality, reflected in the significant positive genetic correlation between infant and toddler emotionality and adult neuroticism, and the negative genetic correlation between infant and toddler shyness and adult extraversion. The latter result is consistent with the phenotypic understanding of extraversion, which includes “an orientation of one’s interests and energies toward the outer world of people and things”^82^ (ergo, antithetical to shyness), as well as with past observational studies^83,84^. The positive genetic correlations of both infant and toddler emotionality and shyness with adult neuroticism are phenotypically consistent, given the features of neuroticism, which include emotional instability and withdrawn behaviours^13,85^.

Third, our study reveals that there are overlapping sources of genetic variation across different types of temperament and other behaviours within infancy and toddlerhood. All genetic correlations between infant and toddler temperament traits were significant (genetic correlations were ±0.24 to ±0.62 for cross-age results) except for those between activity and emotionality and between activity and sociability. This suggests there may be some pleiotropic genetic markers that are associated with multiple infant and toddler temperament traits. All four temperament scales were also significantly genetically correlated with age at onset of walking (for sociability, this was only in the third-year analyses, and for emotionality this was only in the second-year and cross-age analyses). Future research could employ multivariate GWAS genomic structural equation model fitting to understand the multivariate genetic architecture of infant and toddler behaviour in more depth, akin to research conducted for psychiatric disorders^86,87^. Our results enhance our understanding of postnatal human functional brain development by providing empirical evidence on the relative overlap and independence of the genetic pathways underlying different domains of behaviour in the early years^88^.

Fourth, our results reveal two eQTLs underlying infant and toddler temperament. The first eQTL is for activity level. Specifically, the alternate allele of rs1054442 was associated with decreased third-year activity levels and increased expression of *RHEBL1* in the human cortex. This locus has previously been shown to be associated with higher educational attainment^66^ and cognitive performance^67^. It is thus plausible to suggest that infant and toddler activity is genetically associated with both later cognitive performance and educational attainment, as evidenced by the significant negative genetic correlations (Fig. 2 and Supplementary Table 15) and the discovery of this shared eQTL. In terms of the function of this gene, the product of *RHEBL1*, RhebL1, operates as a regulator of NF-κB-mediated transcription^68^ and is involved in the mTOR signalling pathway^69,70^. The mTOR signalling pathway is a signalling hub: it integrates neuronal activity and synaptic inputs to regulate cellular processes that include neuronal structure and thus modulates synaptic plasticity and memory formation^89^. Differences in mTOR signalling have been previously associated with autism, schizophrenia, depression and substance use disorders^89^. In sum, this discovery of an eQTL for *RHEBL1*, which is associated with the activity level of infants and toddlers and is expressed in the brain, delivers biological insight into this early behaviour. It implicates the involvement of the mTOR pathway. Moreover, it reveals that this locus has pleiotropic effects, suggesting that early activity level may be part of the same biological pathway that relates to later cognitive function and educational attainment. Next steps include using functional validation to further understand the mechanisms by which *RHEBL1* is influencing activity level in infants and toddlers.

The second eQTL discovered is for emotionality. We revealed colocalization between the locus for cross-age emotionality and the eQTL for *MR1*. Specifically, the alternate allele for the lead SNP, rs10797666, was associated with increased *MR1* expression in the brain and decreased emotionality. While *MR1* has not yet been linked to neurodevelopmental or behavioural traits, this may be due in part to infant and toddler genetics being under-researched^10^. Given MR1’s role in the immune system^90^, our discovery of an eQTL in *MR1* for emotionality is in line with evidence that the immune system is related to the brain and behaviour^91–94^. Future work with further functional validation is needed to identify mechanisms underlying this eQTL association.

Fifth, we conducted multi-ancestry meta-GWAS as well as European-ancestry ones, providing suggestive evidence of the generalizability of our results. One SNP remained genome-wide significant in the multi-ancestry analyses. The multi-ancestry analyses were limited in scope by the sample sizes, as we were able to identify only two cohorts with relevant data. These multi-ancestry GWAS meta-analyses aimed to replicate the findings from the analyses of European-ancestry participants. Past GWAS have mostly been conducted in European-ancestry samples due to data availability^95^. However, in the future, it is important for more data to become available for diverse-ancestry populations to assess how our findings generalize more widely.

We employed the EAS model of temperament^13^ for both pragmatic and empirical reasons. The model and scales are supported by studies that replicate its factor structure across samples and countries^31–33^. The scale is designed for parent ratings and for the infant and toddler age range. Multiple large, genotyped cohorts have used these scales, thus enabling the analysis here of multiple cohorts together in a single well-powered GWAS. Nevertheless, in terms of our study’s limitations, we note that other models of temperament exist, and thus future research may employ different models of infant and toddler temperament to compare the results. The EAS temperament questionnaire includes quite global traits, while other temperament models include more granularity^37^.

This research employed phenotype data that were collected through parent report. Parents experience their children’s behaviour across time and contexts^19^, and thus parent report holds this advantage over observer-rated temperament, which is typically more time-limited to single or specific occasions and context-specific^96^. In addition, parents are typically the most consistently available raters for most children of this age. However, while parent ratings are a practical solution, they will include some rater bias, which is a limitation. Furthermore, some studies have indicated that parental ratings of some EAS subscales of infant temperament in twin samples include contrast bias^97^ (that is, where variance is inflated in dizygotic (DZ) twins compared with monozygotic twins, due to raters exaggerating differences within DZ twin pairs). In twin studies, contrast effects can upwardly bias heritability estimates. However, the majority (over 80%) of the total GWAS sample included here were not twins, and moreover, while related individuals were included in the GWAS, the analyses did not involve within-sibling comparisons. Any potential contrast effects in the minority of twins in our total sample would therefore have introduced a small amount of error into the measurement, which would have downwardly biased the *h*^2^_SNP_ estimates.

We measured temperament in the second and third postnatal years. We also used the mean phenotype score across these two years, which would be less affected by transient factors. It has recently been demonstrated that taking a mean phenotype across ages improves discoverability in GWAS^50^. Furthermore, it has been shown that childhood behavioural phenotypes captured from multiple ages yield higher heritability estimates than at single ages^98^. Our results concurred with these studies in that the genetic correlations across the second and third years were very high and the point estimates for the SNP heritabilities for the mean cross-age scores were higher than the second- or third-year measures when studied separately.

Considering there are currently few GWAS of behavioural traits in this age range^10^, there is not much data in existing bioinformatic databases on previous gene associations in this age group, which limited the post-GWAS analyses. As the field of infant and toddler genetics grows, it is likely that more mechanistic links will be possible through these gene-based analyses in this age range.

The SNP heritability estimates for sociability were significant but were lower than expected, given previous much higher twin heritability estimates^8,25^. In the largest sample on its own, MoBa, sociability showed low SNP heritability, which reveals that the heterogeneity of measures across cohorts was not causing the low SNP heritability. Tests of heterogeneity were also not significant in the sociability GWAS (Supplementary Note 2). It is possible that the heritability of sociability is deflated due to the effect of individual differences in the size of the child’s social sphere, due to factors such as the number of siblings. If measurement error was high for the sociability scale, the portion of reliable variance would be lower, and the SNP heritability estimate would be underestimated. Nevertheless, despite the modest SNP heritabilities, sociability showed positive genetic overlap with adult extraversion and significant, negative genetic overlap with infant and toddler shyness and with autism, which are consistent with our understanding of these phenotypes.

In this gene discovery research on parent-rated infant and toddler temperament, we identified associations with common genetic variants. We have shown that infant and toddler temperament share common genetic associations with similar behavioural domains later in life. For example, activity level shares genetic variation with later-diagnosed ADHD, sociability is negatively genetically associated with later-diagnosed autism, emotionality shares genetic variation with adult neuroticism and shyness is negatively genetically associated with adult extraversion. Our results reveal that genetic variants associated with infant and toddler traits across motor, activity, social and emotional domains are not independent of one another: there are pleiotropic genetic associations across dimensions of temperament. We identified ten independent genome-wide significant loci underlying infant and toddler temperament, providing targets for future biological follow-up. In particular, we discovered two eQTLs for *RHEBL1* and *MR1* that are expressed in the brain and that colocalize with activity and emotionality, respectively, which offer insight into the underlying biological pathways. An exciting next step will be to investigate how the early environment, which plays a critical role in early childhood, interacts and correlates with the genetic associations underlying infant and toddler temperament. Furthermore, the PGS from our study can be employed across disciplines to offer a fuller understanding of the genetic associations underlying child development. Intervention and support are maximally impactful when they begin early, offering the most hope for optimizing children’s outcomes. Fundamental research on the aetiology of early behaviour such as this can strengthen the design of support systems for children from the earliest years. Finally, our results suggest that infants and toddlers vary in their temperament, and this variation is associated with common genetic differences.

## Methods

This study was preregistered on the Open Science Framework (https://osf.io/2yznd) on 18 January 2024. Deviations from the preregistration are outlined in Supplementary Note 4. Individual cohorts followed a standard operating procedure (SOP) document (https://osf.io/jyk6d/). The overall study design is depicted in Fig. 4.

### Ethics and inclusion

This study conforms to all relevant ethics regulations. This study was approved by the departmental ethics committee for the School of Psychological Sciences at Birkbeck, University of London (reference no. 2021007) on 27 October 2020. Each of the cohorts included in this research received ethical approval from their local ethics committees. Researchers from each of the cohorts included were involved in the study implementation and are included as authors on this paper.

### Cohorts and measures

The sample sizes included in the GWAS for each sample entered into the meta-analyses are detailed in Supplementary Table 1.

### MoBa

MoBa is a population-based pregnancy cohort study conducted by the Norwegian Institute of Public Health^99^. Participants were recruited from all over Norway from 1999 to 2008. The women consented to participation in 41% of the pregnancies. Blood samples were obtained from both parents during pregnancy and from mothers and children (umbilical cord) at birth^100^. The cohort includes approximately 114,500 children, 95,200 mothers and 75,200 fathers. The establishment of MoBa and initial data collection were based on a licence from the Norwegian Data Protection Agency and approval from the Regional Committees for Medical and Health Research Ethics. The MoBa cohort is currently regulated by the Norwegian Health Registry Act. The current study was approved by the Regional Committees for Medical and Health Research Ethics (2016/1702). We used the year of birth and child’s sex from the Medical Birth Registry, a national health registry containing information about all births in Norway from 1967 onwards^101^.

The quality-controlled genotype dataset from MoBa includes 76,577 children of European genetic ancestry and 6,981,748 SNPs that passed quality control^102^. We used phenotypic data from the 18- and 36-month data collection sweeps. After we filtered out individuals with no relevant phenotype data, there were *n* = 55,269–55,337 (27,010–27,042 females and 28,256–28,295 males) in the second-year analyses, *n* = 42,724–42,754 (20,903–20,913 females and 21,821–21,841 males) in the third-year analyses and *n* = 39,234–39,290 (19,218–19,250 females and 20,009–20,040 males) in the cross-age analyses.

### ALSPAC

The ALSPAC (also known as ‘Children of the 90s’) study is a multi-generational observational prospective birth cohort^29,103^. Pregnant women resident in Avon, UK, with expected dates of delivery between 1 April 1991 and 31 December 1992 were invited to take part in the study. The initial number of pregnancies enrolled was 14,541, resulting in 13,988 children included in the study who were alive at 1 year of age. Of these pregnancies, some were from the same mother, meaning that there were 14,203 unique mothers who were initially enrolled in the study. Additional recruitment provided a total of 14,833 unique women enrolled in ALSPAC as of September 2021. Ethical approval for the study was obtained from the ALSPAC Ethics and Law Committee and the Local Research Ethics Committees. Consent for biological samples was collected in accordance with the Human Tissue Act (2004). Informed consent for the use of data collected via questionnaires and clinics was obtained from participants following the recommendations of the ALSPAC Ethics and Law Committee at the time.

After quality control and imputation of genetic data, 8,972 children (4,355 females and 4,572 males) remained in the sample, and 27,384,358 genetic variants were available for analysis. We used phenotypic questionnaire data from the 24- and 38-month data collection sweeps. The ALSPAC cohort was not included in the cross-age sociability analysis because the sociability phenotype was available in only the 38-month data collection sweep. After we filtered out individuals with no relevant phenotype data, there were *n* = 6,955–6,966 (3,382–2,287 females and 3,573–3,579 males) in the second-year analyses, *n* = 6,850–6,855 (3,341–3,343 females and 3,508–3,513 males) in the third-year analyses and *n* = 6,418–6,425 (3,127–3,131 females and 3,291–3,296 males) in the cross-age analyses.

The study website contains details of all the data that are available through a fully searchable data dictionary and variable search tool: http://www.bristol.ac.uk/alspac/researchers/our-data/.

### TEDS

TEDS^104^ is a large community-based study of twins born in England and Wales that aims to investigate the relative genetic and environmental influences on individual differences in developmental, cognitive and emotional factors relevant to typical development. All twins born in England and Wales were invited to participate in TEDS through their parents; the twins were identified through the Office for National Statistics. The first wave of data collection took place when the twins were 18 months old, in which 13,759 families participated. After the first wave, data were collected at regular intervals throughout development and into adulthood. Informed consent was obtained through parents^105^. Analyses of TEDS data were conducted on the King’s College London CREATE HPC^106^.

Genetic data from TEDS twins were genotyped on two different genotype chips: Affymetrix GeneChip v.6.0 (*n* = 3,057 individuals) and Illumina HumanOmniExpressExome (*n* = 7,289 individuals). After quality control and imputation, 7,363,646 SNPs remained. Only one of each monozygotic twin pair was genotyped and is therefore included in these analyses. This analysis used data from the TEDS data collection waves at ages 2 and 3. After we filtered out those with no relevant phenotype data, there were *n* = 5,460–5,527 (2,825–2,861 females and 2,635–2,671 males) in the second-year analyses, *n* = 5,648–5,686 (2,907–2,924 females and 2,741–2,762 males) in the third-year analyses and *n* = 4,673–4,769 (2,422–2,472 females and 2,251–2,297 males) in the cross-age analyses.

The TEDS cohort was not included in the cross-age shyness analysis because the shyness phenotype was available in only the age 3 data collection sweep.

### Lifelines

The Lifelines cohort study is a multidisciplinary prospective population-based cohort study examining in a unique three-generation design the health and health-related behaviours of 167,729 persons living in the North of the Netherlands. It employs a broad range of investigative procedures in assessing the biomedical, socio-demographic, behavioural, physical and psychological factors that contribute to the health and disease of the general population, with a special focus on multi-morbidity and complex genetics

Lifelines recruited individuals aged 25–50 residing in the North of the Netherlands between 2006 and 2013^107^. After their recruitment, in their initial study visit, individuals were asked for their consent for the study team to contact their family members with an invitation to participate. This method of recruitment thus gave rise to the three-generation design that the study employs, with cohort members as young as six months old.

Participants in the Lifelines study were sent a questionnaire every 1.5 years, which included a parent-report questionnaire for the children in Lifelines, in Dutch^107^. Only the activity phenotype was available for Lifelines. Questionnaires about the offspring in the sample were completed retrospectively by parents or caregivers, and questions specified the relevant time period in the child’s life on which to retrospectively report. After genetic data quality control and after we filtered out anyone without the relevant phenotype data, the sample size for Lifelines was *n* = 3,435 (1,780 females and 1,655 males).

The Lifelines cohort was included only in the analyses for the second year because it did not have relevant data for the third year.

### NTR

NTR was established and started recruiting participants in 1986. There are two branches to this study, consisting of twins registered shortly after birth (Young NTR) and those recruited as adolescents or young adults^30^ (Adult NTR). The present study uses data from Young NTR^108^. In Young NTR, recruitment took place shortly after birth during a home visit to parents of newborn twins or multiples^30^. The parents completed questionnaires about their offspring in roughly two-year intervals from birth. For a selection of these twins, DNA samples were collected^109^. The genotyped sample in NTR consisted of *n* = 37,823 subjects. After quality control and the removal of anyone without relevant temperament phenotype data, the GWAS sample size was *n* = 1,433 (722 females and 711 males). We used phenotypic data from the data collection sweep when the twins were approximately two years old. The NTR cohort was included only in the analyses for the second year because it did not have relevant data for the third year.

### TMM BirThree

The TMM study was established in 2012 in Japan^110^ to monitor the health impacts of the Great East Japan Earthquake and the tsunami that followed in 2011. Part of this project is TMM BirThree, a longitudinal birth cohort study that started recruitment in 2013^111^. TMM BirThree is a three-generational study: pregnant women, their partners (the father of the proband), their parents (the grandparents), any siblings of the proband and extended family members were recruited to the study. Participants were recruited between 2013 and 2017 when their delivery was scheduled (with an expected delivery date after 1 February 2014); they were recruited from the Miyagi prefecture, where the Great East Japan Earthquake happened, by their obstetrics clinics and hospitals. A total of 23,406 pregnancies were recruited, with some people participating with multiple subsequent births^111^. Exclusion criteria included non-consent, being unable to complete the study questionnaires and participation in another birth cohort study for that specific pregnancy. Questionnaires pertaining to the child were mailed to the parents; questionnaire data from the data collection sweep when the children were 24 months old were used in the present study.

The final sample included *n* = 5,618–5,653 individuals with relevant phenotypic data for this study (2,750–2,762 females and 2,868–2,891 males). These individuals were all genotyped on the Affymetrix Axiom Japonica v.2 array, which was designed for this specific population^112^.

### DCHS

The DCHS is a population-based cohort study based in Drakenstein, South Africa, which is a peri-urban area outside of Cape Town^113^. Pregnant women were recruited during their second trimester from two primary care clinics in Drakenstein^114^. Enrolled mother–child dyads are being continuously followed up^114^. The study aims to investigate the early determinants of health, including neurodevelopment, in the context of low-income and middle-income countries, which are underrepresented and therefore underserved by research of this kind^115^. Phenotypic data from the DCHS used in the present study were collected during the data collection sweeps at 24 and 42 months.

This cohort included *n* = 595–843 genotyped individuals with relevant phenotype data: *n* = 595–596 (307 females and 288–289 males) for the second-year analyses and *n* = 843 (428 females and 415 males) for the third-year analyses. The genetic data (*n* = 956) were genotyped in three batches: two used the Infinium Global Screening Array (with one batch for cord blood, *n* = 152, and one for blood, *n* = 686), and one used the Infinium PsychArray (using cord blood, *n* = 118).

### Phenotype measurement and coding

We preregistered to study four parent-rated infant and toddler temperament traits: emotionality, activity, shyness and sociability. Three cohorts, NTR, MoBa and ALSPAC (for the third-year data collection), included the EAS measure, and as such these scales were used as included in the cohorts. We note that NTR and ALSPAC used the scales as published, and MoBa’s EAS scales were reduced in items (see the instrument documentation at https://www.fhi.no/en/ch/studies/moba/for-forskere-artikler/questionnaires-from-moba/). For Lifelines, TEDS and ALSPAC (in the second year), we used appropriate items and scales from other parent-report temperament questionnaires that captured the same four EAS temperament constructs. ALSPAC (second-year data collection) used published subscales from other measures that captured the emotionality and activity constructs, and we therefore used these published subscales. Where cohorts did not include emotionality, activity, shyness or sociability subscales (that is, Lifelines, TEDS and the second year of ALSPAC for shyness), items that matched the EAS items were used to create subscales. See Supplementary Note 5 for further details of phenotype measurement in each cohort.

In all cohorts, the phenotypes were measured using Likert-scale questionnaires completed by parents or caregivers; for the details of questionnaire raters, see Supplementary Table 22. The individual’s phenotype score was calculated as a prorated sum score or mean of questionnaire items, scaled about a mean of 0 and standard deviation of 1. A completion rate of 50% was required; otherwise, the phenotype was coded as missing. In the cross-age analyses, the phenotype was calculated as a mean of the phenotype values from the second and third years. For phenotypic distributions of the cross-age phenotypes, see Supplementary Figs. 19–21.

We conducted separate phenotypic analyses to verify the comparability of the different measures for the EAS traits where the EAS temperament survey was not employed by the cohorts. This did not include the phenotype measures from TMM BirThree and the DCHS (the Child Behaviour Checklist questionnaire). Briefly, in a separate infant and toddler sample (*n* = 118 for the second year; *n* = 84 for the third year), we collected parent-report data using all of the temperament measures used by the cohorts employed in this meta-analysis to assess concordance between phenotype measurements in different samples. The methods and results of these phenotypic analyses are provided in Supplementary Note 6.

In light of past research showing that genetic associations are stable across infancy and toddlerhood^25,116^, we have investigated temperament traits here both at specific ages (the second and third postnatal years) and as an average of the two years. Three cohorts had data for both ages: MoBa, ALSPAC and TEDS. For each cohort, the phenotype needed to be measured at both time points to be included in the cross-age analyses, because a mean of the phenotype across ages was used in these analyses. Sociability in ALSPAC and shyness in TEDS were thus not included in the cross-age analyses. The cross-age analyses were not preregistered.

### Rater information

The MoBa and NTR phenotypes were measured using maternal-report questionnaires. ALSPAC used caregiver-report questionnaires; that is, the questionnaires could be completed by any combination of mother, father and/or ‘other’. The Lifelines phenotype was reported retrospectively by either biological parent or ‘other’. There was no evidence of significant differences between the mean reported phenotypes by different raters in the ALSPAC or Lifelines samples (Supplementary Note 7).

### Quality control of genetic data

The main details of the procedures for individual cohort dataset genotyping, imputation and quality control that were conducted by the study teams are outlined in Supplementary Table 23 and are also detailed elsewhere^29,102,105,107,109^. An overview of the genotyping, ancestry filtering and imputation of the datasets as well as the quality-control procedures conducted for the present study can be found in Supplementary Table 23. Each sample underwent post-imputation quality control for the present study according to the guidelines from the Rapid Imputation for Consortias Pipeline (RICOPILI)^117^. Briefly, genetic variants with a low imputation score, minor allele frequency or call rate were removed, as well as variants that had a low *P* value for the Hardy–Weinberg equilibrium exact test. Individuals were removed if they had a low call rate, excess autosomal heterozygosity, an XXY genotype or other aneuploidies or if they fell outside of the relevant ancestry cluster when compared with a reference dataset (the filter thresholds are listed in Supplementary Table 23). Where X-chromosome data were available, reported sex was compared with sex according to X-chromosome heterozygosity, and individuals with mismatches were removed. The genetic relatedness of individuals was compared to the reported family structure, and any mismatches were removed as they were likely to be sample mix-ups.

### GWAS

GCTA^118^ fastGWA^119^ was used to carry out genome-wide association analyses in each individual European-ancestry sample. In all association analyses, the first ten principal components and age (or date of birth as a proxy in MoBa) were included in the model as quantitative covariates, and sex and any relevant technical variables (for example, genotyping batch) were included as discrete covariates. A genetic relatedness matrix was also included to correct for relatedness structure within the sample. See Supplementary Note 8 for a full list of covariates included for each sample.

In each European-ancestry sample, GWAS analyses were completed for the whole sample, and also stratified by sex, for each of the four EAS traits in the second and third years and as a cross-age average.

### GWAS meta-analysis

All summary statistics from individual sample GWAS went through quality control using GWASinspector (v. 1.6.5)^120^, which removed variants that had missing or invalid data in key columns (chromosome, position, *β*, *β* s.e., A1 and A2); were duplicated; were monomorphic, allosomal or mitochondrial; or had a low imputed information score (<0.8) where available. GWASinspector also compared variants to the 1000 Genomes reference panel^121^, removed variants that were not in this reference dataset and aligned variants to the reference strand.

The meta-analysis of summary statistics was conducted using METAL (version release date: 2020-05-05)^80^ using a standard-error-weighted fixed-effects model. Variants were included only if they had combined minor allele frequency >1% and if the rsID had matching positions between cohorts (if not, the sample variant from the sample with the higher *n* was retained). Meta-analyses were conducted for the whole-sample GWAS and for the sex-stratified GWAS. The genetic correlations between each of the pairs of sex-stratified meta-analyses for each phenotype were calculated. The total meta-analysed sample sizes are provided in Supplementary Table 1.

The results from all meta-analyses were filtered by the per-variant sample size. A filter was applied such that variants were required to have, at the least, an *n* totalling either the summed *n* of the cohorts not including MoBa, or 10,000, whichever was lower. The analyses for which the summed sample sizes of the cohorts not including MoBa were less than 10,000 were shyness in the second year, and shyness and sociability in the cross-age analyses.

Heterogeneity in the meta-analyses was assessed using METAL^80^, using *I*^2^ statistics and corresponding *P* values from Cochran’s *Q*-tests for each SNP. *P* values were assessed according to a genome-wide significance threshold of *P* < 5 × 10^−8^.

### Multi-ancestry GWAS meta-analysis

We conducted GWAS meta-analyses including diverse-ancestry cohorts: TMM BirThree, a Japanese cohort study, and the DCHS, a cohort based in South Africa. The GWAS in TMM BirThree were conducted using GCTA fastGWA to account for known relatedness within the sample (see the methods above), and the GWAS in the DCHS was conducted using PLINK 2.0^122^ because there were no related individuals in the sample. These individual GWAS analyses and the meta-analyses including all cohorts used the same methods as those using only European-ancestry samples. The summary data underwent quality control using GWASinspector^120^ as above, and then the summary data were meta-analysed using a fixed-effects standard-error-weighted meta-analysis in METAL^80^. METAL was also used to conduct heterogeneity analyses, as above. SNPs were retained in the meta-analysed summary statistics if they had a minimum sample size of *n* = 10,000.

Details of the quality control conducted for these two diverse-ancestry samples are provided in Supplementary Table 23 as well as elsewhere^115,123^. Quality control of genetic data was conducted in line with the study SOP, as with the European-ancestry samples.

The phenotype in both cohorts was measured using the Child Behaviour Checklist parent-report questionnaire^79^. Prorated sum scores were calculated as above for the European-ancestry cohorts. See Supplementary Note 5 for further details.

Most post-GWAS analyses were not conducted using summary statistics from diverse-ancestry GWAS meta-analyses due to differences in LD structure between different ancestry populations and the need for accurate LD reference panels^95^.

### Conditional analysis of lead variants

For GWAS that identified multiple genome-wide significant signals (*P* < 5 × 10^−8^) in the same locus, conditional analyses were performed using GCTA^118^ COJO^51^. This was done to identify whether there are multiple independent signals in a locus by conditioning on the lead SNP and testing for further signal independent of that SNP at the locus. Stepwise selection was used to identify independent lead variants in a single locus, with MoBa data used to estimate LD between SNPs.

### FUMA

To further investigate the COJO independent SNPs as identified above, we carried out fine-mapping, functional annotation, chromatin-interaction mapping and gene-based analyses in FUMA^53^ (v.1.5.2) and MAGMA^53,54^ (v.1.08). A list of the COJO independent SNPs was input as a predefined set of lead SNPs. To carry out analyses on prioritized genes mapped to SNPs significantly associated with our phenotypes of interest, SNPs were defined to be independent if they had pairwise LD *r*^2^ < 0.6. LD blocks were merged into one locus if they were closer than 250 kb to one another. The 1000 Genomes Phase 3 EUR reference panel was employed by FUMA in these analyses.

Annotation was carried out using ANNOVAR within FUMA (downloaded on 17 July 2017). SNPs were mapped to genes at a maximum distance of 10 kb away using tissue types related to brain, nervous system, immune response, hormones, physical fitness and development for chromatin state mapping and annotation datasets using the following relevant HiC datasets: adult cortex and fetal cortex, adrenal, dorsolateral prefrontal cortex, hippocampus, lung, ovary, psoas, right ventricle, small bowel, spleen, mesenchymal stem cell, mesoderm and neural progenitor cell. We annotated enhancer/promoter regions from Roadmap 111 epigenomes of the following tissues: adrenal, brain, embryonic stem cells, embryonic stem cell derived, fat, column, duodenum, oesophagus, intestine, rectum, stomach, heart, lung, muscle, muscle leg, ovary, skin, spleen, stromal connective, thymus and induced pluripotent stem cells. Within FUMA, the GWAS Catalog^124^ (v.1.0.2, e112 r2024-06-17) was searched to identify prior GWAS for which candidate SNPs in the present GWAS were also significant.

eQTL mapping was performed in FUMA using the following relevant GTEx v.8 tissues: Adipose Subcutaneous, Adipose Visceral Omentum, Brain Amygdala, Brain Anterior cingulate cortex BA24, Brain Caudate basal ganglia, Brain Cerebellar Hemisphere, Brain Cerebellum, Brain Cortex, Brain Frontal Cortex BA9, Brain Hippocampus, Brain Hypothalamus, Brain Nucleus accumbens basal ganglia, Brain Putamen basal ganglia, Brain Spinal cord cervical_c-1, Brain Substantia nigra, Colon Sigmoid, Colon Transverse, Esophagus Gastroesophageal Junction, Esophagus Mucosa, Esophagus Muscolaris, Heart Atrial Appendage, Heart Left Ventricle, Lung, Muscle Skeletal, Nerve Tibial, Ovary, Pituitary, Cells Cultured Fibroblasts, Skin Not Exposed Suprapubic, Skin SunExposed Lower Leg, Small Intestine Terminal Ileum, Spleen, Stomach, Testis and Thyroid.

MAGMA gene-property analyses (one-sided test of a positive association between tissue specificity and genetic association with the temperament trait) were performed using GTEx v.8 (ref. ^125^) and BrainSpan^126^ (consisting of RNAseq data from 524 brain samples from 11 general developmental stages ranging from early prenatal to middle adulthood) tissue types. For MAGMA gene-set analysis, 4,761 curated gene sets and 5,917 Gene Ontology terms from MsigDB v.6.2 (ref. ^127^) provided within FUMA were used. The MHC region was excluded for all analyses except for activity in the second year and activity cross-age analyses, for which there were significant COJO independent SNPs in this region. SNPs were assigned to genes if they were within 1 Mb upstream and downstream^53^.

### LDSC

SNP heritability and bivariate genetic correlations were calculated using LDSC^72,74^, using the 1000 Genomes Phase 3 (ref. ^121^) European genetic ancestry LD score reference panel. During data munging, variants were retained only if they matched a reference panel of high-quality Haplotype Reference Consortium variants. Two-sided tests were used to evaluate the null hypothesis (*r*_g_ = 0).

Bivariate genetic correlations were calculated between each of the four EAS traits and other physical health, cognitive, neurodevelopmental, psychiatric and personality phenotypes. The traits included in these genetic correlation calculations were age at onset of walking^46^, BMI^128^, educational attainment^58^, cognitive performance (“intelligence”)^67^, lifetime anxiety disorder^129^, depression^130^, schizophrenia^131^, bipolar disorder (types 1 and 2, not including self-diagnosis)^132^, autism (pre-publication access to early freeze of upcoming GWAS), autism diagnosed before age 11 and autism diagnosed after age 10 (ref. ^133^), ADHD^134^ and the ‘big five’ personality traits, neuroticism, extraversion, agreeableness, conscientiousness and openness to experience^135^.

A Benjamini–Hochberg procedure was applied to control the FDR at 0.05. Any genetic correlations that reached significance under this criterion were followed up with MiXeR analyses (below; where further criteria were met).

### MiXeR

Univariate causal mixture models were conducted using MiXeR^76^ to determine polygenicity estimates—that is, the number of SNPs that contribute to 90% of the SNP heritability. Bivariate causal mixture models were also fitted using MiXeR^75^ using all phenotype pairs identified by LDSC genetic correlation analyses. This was applied only when both phenotypes in the pair satisfied the criterion of *h*^2^_SNP_ × *n* > 6,000 as specified by ref. ^136^ to ensure there was enough statistical power for the analyses. For all phenotypes in these analyses, we removed the MHC region (chr. 6: 26000000–34000000; GRCh37) due to its complex LD structure, and for the GWAS summary statistics for which a case–control design was employed, we calculated the effective sample size as *n*_eff_ = 4/(1/*n*_cases_ + 1/*n*_controls_), as indicated by the authors. We used the authors’ summary statistics preparation pipeline to prepare all of the files being entered into both univariate and bivariate analyses (https://github.com/precimed/python_convert/).

Akaike information criterion values were used to compare the MiXeR model to a model that contains the minimum amount of polygenic overlap to explain the LDSC genetic correlation (‘minimal model’) for all bivariate MiXeR models. Positive differential Akaike information criterion values indicated that the MiXeR model was supported over the minimal model.

### PGS

The variance explained by the PGS for each trait was calculated using a leave-one-out design. GWAS meta-analyses for each trait were conducted leaving out one sample in turn each time, and summary statistics were used to calculate PGS weights. The left-out sample was then used as a target sample to evaluate the PGS. This was repeated, leaving out each of the smaller samples (ALSPAC, TEDS, NTR and Lifelines), for each of the phenotypes separately. Given that the MoBa sample comprised the largest portion of the overall sample size, it would not be appropriate to leave it out as a target sample. A within-sample cross-validation method was therefore used for MoBa whereby one fifth of the sample was left out as a target sample and the rest of the sample used for a GWAS from which the summary statistics were employed to estimate the SNP weights.

All PGS were calculated using PRS-cs^137,138^, with weights derived per chromosome using the 1000 Genomes Phase 3 European panel^121^ as an LD reference panel. PLINK 2.0 (ref. ^122^) was used for computing PGS in the left-out target sample. The following PRS-cs parameters were used: the number of burn-in iterations was 500; the total number of MCMC iterations was 1,000; parameters a and b in the gamma-gamma prior were 1 and 0.5, respectively; the thinning factor of the Markov chain was 5; the global shrinkage parameter phi was 0.01; and the SNP effect sizes were standardized.

We quantified the proportion of variance explained using the adjusted *R*^2^ of the linear model regressing the PGS on the phenotype (both scaled about a mean of 0 and an s.d. of 1), controlling for ten genetic principal components and the genotyping batch (if applicable to the target sample)

### Within- and between-family PGS analysis

We conducted within- and between-family PGS analyses to understand their relative contributions to each phenotype^78^. This was conducted in the TEDS and NTR samples (NTR only for specific-age analyses; the results are shown in Supplementary Fig. 14 and Supplementary Table 18), which contain DZ twin pairs, using a meta-analysis of the other samples to calculate PGS weights (as described above). We used data for DZ pairs with complete phenotypic data. First, bootstrapped intra-class correlations were calculated to ensure that the method was justified. The intra-class correlation represents the portion of the overall variance in the phenotype that can be accounted for by the within-pair similarity. When it tends to 0, the total variation is mostly attributed to individual-level variation. We used the method from Selzam et al.^78^, with scripts from Demange et al.^139^ used (https://github.com/PerlineDemange/GeneticNurtureNonCog/). For each individual, the between-family PGS was calculated as the mean PGS of that individual and their sibling pair. The within-family PGS was calculated as that individual’s PGS minus their between-family PGS.

We fitted a random-intercept mixed-effects linear model in R^140^, including age, sex, the first ten principal components and a genotyping batch dummy variable as covariates. A chi-squared test was used to compare the within- and between-family regression coefficients.

### Colocalization

We used the coloc method^64^ with the default priors to assess whether GWAS and eQTL signals in the same region are likely to share a single causal variant. Colocalization was performed for each GWAS with at least one genome-wide significant SNP. For each significant locus, we tested all genes within ±1 Mb of the lead SNP, as well as genes prioritized by FUMA. eQTL data were derived from 1,433 post-mortem bulk RNA-seq samples of the adult human cortex^65^. A PP_4_ > 0.8 threshold was applied to identify significant colocalization events, where PP_4_ represents the posterior probability that both the GWAS and eQTL signals share a common causal variant. A PP_4_ value greater than 0.8 indicates strong evidence for colocalization, suggesting that the signals from both datasets are likely to be driven by the same underlying variant. Colocalization analyses were not performed for hits in the human leukocyte antigen (HLA) region on chromosome 6 due to the high LD complexity in this region, which complicates the identification of independent causal variants.

### Reporting summary

Further information on research design is available in the Nature Portfolio Reporting Summary linked to this article.

## Supplementary information

Supplementary Information Supplementary Figs. 1–27, Notes 1–8 and Table 24.

Reporting Summary

Supplementary Tables Supplementary Tables 1–23, 25 and 26.

## Data Availability

The summary statistics of the genome-wide association study of infant temperament traits are available via Figshare at https://doi.org/10.6084/m9.figshare.30041575 (ref. ^141^). Data from MoBa and the Medical Birth Registry of Norway used in this study are managed by the National Health Register Holders in Norway (Norwegian Institute of Public Health) and can be made available to researchers, with approval from the Regional Committees for Medical and Health Research Ethics, compliance with the EU General Data Protection Regulation and approval from the data owners. The consent given by the participants is not open to the storage of data on an individual level in repositories or journals. Researchers who want access to datasets for replication should apply through https://helsedata.no/. Access to datasets requires approval from the Regional Committee for Medical and Health Research Ethics in Norway and an agreement with MoBa. Data from NTR are available upon request by researchers. Information is available at https://ntr-data-request.psy.vu.nl. Lifelines data may be obtained from a third party and are not publicly available. Researchers can apply to use the Lifelines data used in this study. More information about how to request Lifelines data and the conditions of use can be found on their website at https://www.lifelines-biobank.com/researchers/working-with-us. The informed consent obtained from ALSPAC participants does not allow the data to be made available through any third-party-maintained public repository. Supporting data are available from ALSPAC on request under the approved proposal number, B3360. Full instructions for applying for data access can be found here: http://www.bristol.ac.uk/alspac/researchers/access/. The ALSPAC study website contains details of all the available data (http://www.bristol.ac.uk/alspac/researchers/our-data/). The TEDS data are accessible to researchers following the procedure outlined in their data access policy (https://www.teds.ac.uk/researchers/teds-data-access-policy/). The data that support the findings of this study are available from the Tohoku Medical Megabank project, but restrictions apply to the availability of these data, which were used under licence for the current study and so are not publicly available. The data are, however, available upon request and with the permission of the Tohoku Medical Megabank project (dist@megabank.tohoku.ac.jp). The DCHS is committed to the principle of data sharing. De-identified data will be made available to requesting researchers as appropriate. Requests for collaborations to undertake data analysis are welcome. More information can be found on their website (http://www.paediatrics.uct.ac.za/scah/dclhs). eQTL results for the ROSMAP, Mayo TCX, Mayo CER and cortical meta-analysis from Sieberts et al.^65^ are available through the AMP-AD Knowledge Portal at https://www.synapse.org/#!Synapse:syn17015233.
