# Supplementary material for: Genome-wide association studies of infant and toddler temperament in European and multi-ancestry populations
Source: Nat Hum Behav. 2026 Jul 1;10(9):1821–40. doi: 10.1038/s41562-026-02486-5 (PMC13590410; doi:10.1038/s41562-026-02486-5)
Supplement: Supplementary file 1 [file 41562_2026_2486_MOESM1_ESM.pdf]

nature human behaviour

Supplementary information https://doi.org/10.1038/s41562-026-02486-5

Genome-wide association studies of infant and toddler temperament in European and multi-ancestry populations

In the format provided by the authors and unedited

Supplementary materials for 'Genome-wide association studies of infant and toddler temperament reveal genetic links with later life outcomes'

Supplementary Figures 2
1. Regional plots for the independent genome-wide significant loci associated with cross-age Emotionality 2
2. Regional plots for the independent genome-wide significant loci associated with cross-age Activity 3
3. Regional plots for the independent genome-wide significant loci associated with cross-age Shyness 4
4. Regional plots for the independent genome-wide significant loci associated with specific age temperament traits 5
5. Manhattan and QQ plots for GWAS meta-analyses of cross-age infant temperament traits 6
6. Manhattan and QQ plots for GWAS meta-analyses of temperament traits in the second year 7
7. Manhattan and QQ plots for GWAS meta-analyses of temperament traits in the third year 8
8. Enrichment of up- and down-regulated DEGs for Activity in the second year and third year 9
9. MAGMA tissue expression analysis for cross-age Shyness 11
10. eQTL plot for colocalisation of rs1054442 in GWAS of activity in the third year and *RHEBL1* 12
11. Overlap between significant lead SNP for activity in the third year and lead SNPs for educational attainment and cognitive performance 13
12. eQTL plot for colocalisation of rs10797666 in GWAS of cross-age emotionality and *MR1* 14
13. Inter-temperament genetic correlations for the second year and third year in infancy 15
14. Polygenic score analysis for second-year temperament 16
15. Polygenic score analysis for third-year temperament 17
16. Manhattan and QQ plots for multi-ancestry GWAS meta-analyses of temperament in the second year 18
17. Manhattan and QQ plots for multi-ancestry GWAS meta-analyses of temperament in the third year 19
18. Multi-ancestry polygenic score analyses 20
19. Phenotypic distribution of the cross-age temperament phenotypes in MoBa 21
20. Phenotypic distribution of the cross-age temperament phenotypes in ALSPAC 22
21. Phenotypic distribution of the cross-age temperament phenotypes in TEDS 23

Supplementary Notes 24
1. Heritability trend increase 24
2. Heterogeneity analyses 26
3. Univariate and bivariate causal mixture modelling 29
4. Preregistration deviations 1
5. Phenotype measures in each cohort 1
6. Agreement between scales across samples 2
7. Mean differences in reported phenotype according to rater in Lifelines and ALSPAC 4
8. Covariates used in each sample GWAS 5

Supplementary Figures

1. Regional plots for the independent genome-wide significant loci associated with cross-age Emotionality

rs10797666 (Chr1: 181018799)

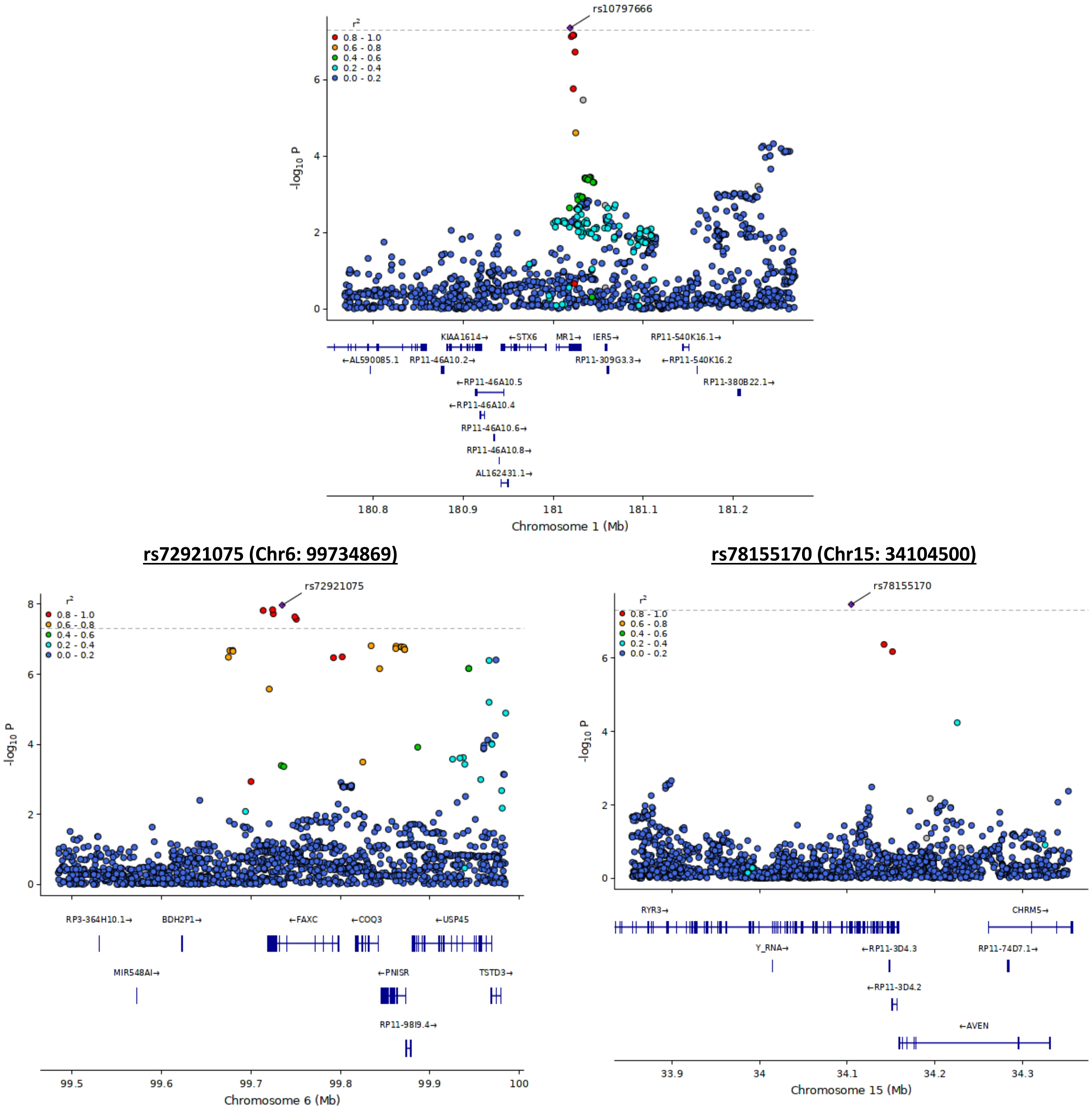

Supplementary Fig. 1 | Regional plots for the three lead independent SNPs associated with cross-age Emotionality. Association p-values are given on the y-axis ($-\log_{10}$[p-value]), chromosomal position is given on the x-axis. Each lead SNP is indicated by a purple diamond, and other SNPs are colour-coded according to their LD with the lead SNP, using the 1000 Genomes[1] European reference panel. Gene positions within the locus are shown along the bottom of each plot.

2. Regional plots for the independent genome-wide significant loci associated with cross-age Activity

rs1064627 (Chr6:30698541)

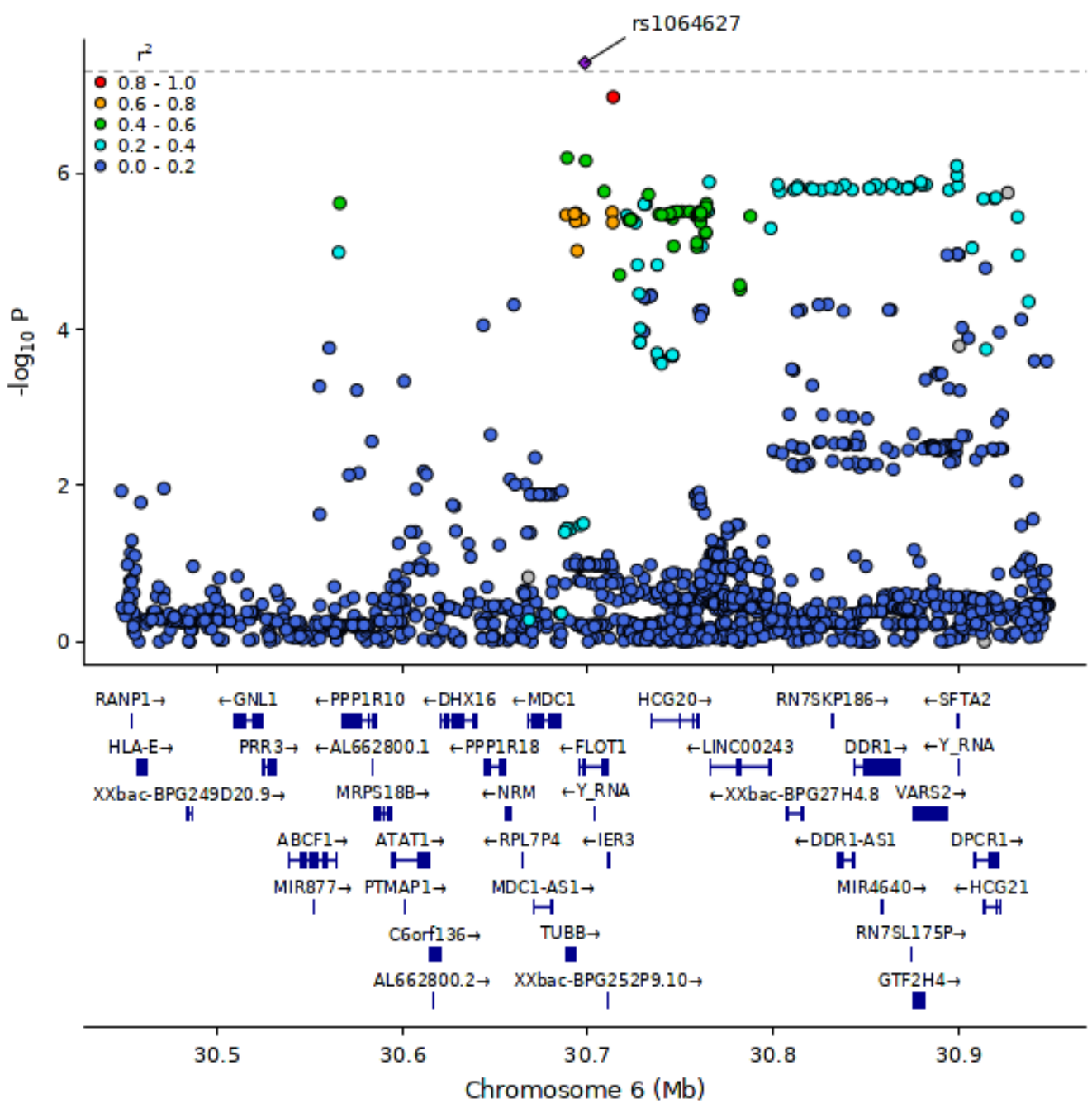

rs1744758 (Chr20:35737816)

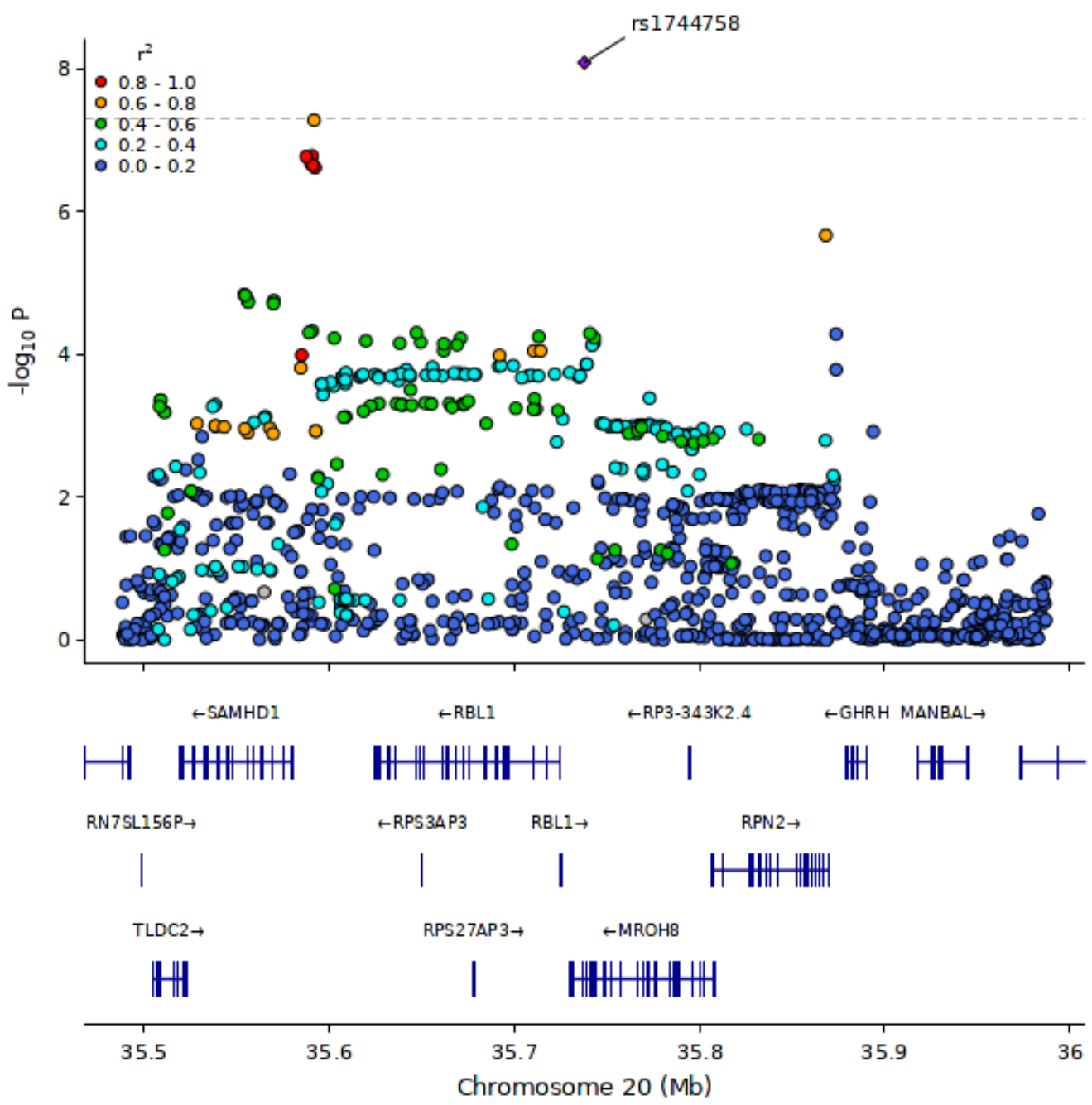

Supplementary Fig. 2 | Regional plots for the two lead independent SNPs associated with cross-age Activity. Association p-values are given on the y-axis (-log$_{10}$[p-value]), chromosomal position is given on the x-axis. Each lead SNP is indicated by a purple diamond, and other SNPs are colour-coded according to their LD with the lead SNP, using the 1000 Genomes[1] European reference panel. Gene positions within the locus are shown along the bottom of each plot.

3. Regional plots for the independent genome-wide significant loci associated with cross-age Shyness

rs79263836 (Chr2:28646937)

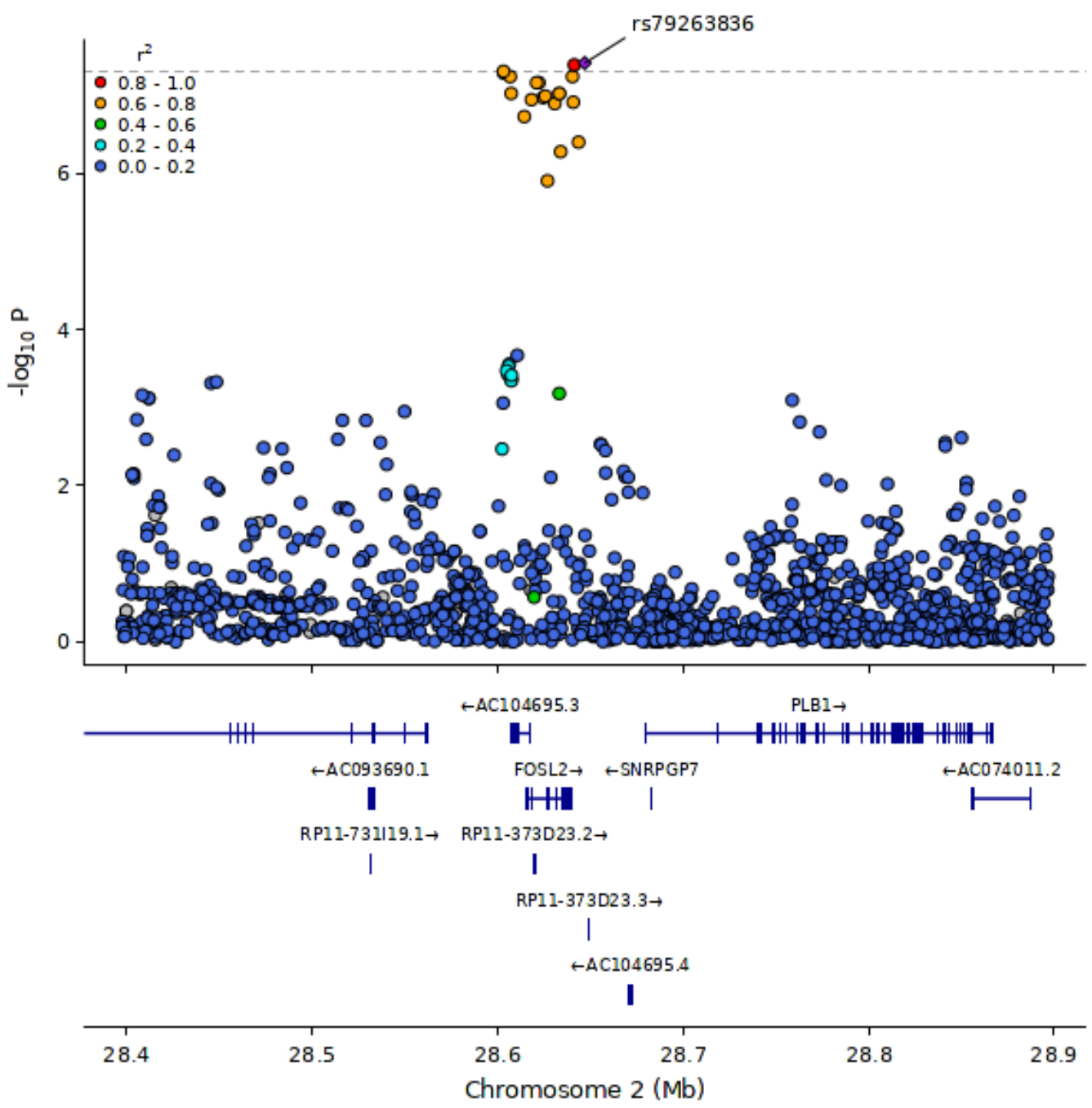

rs12173930 (Chr6:78186434)

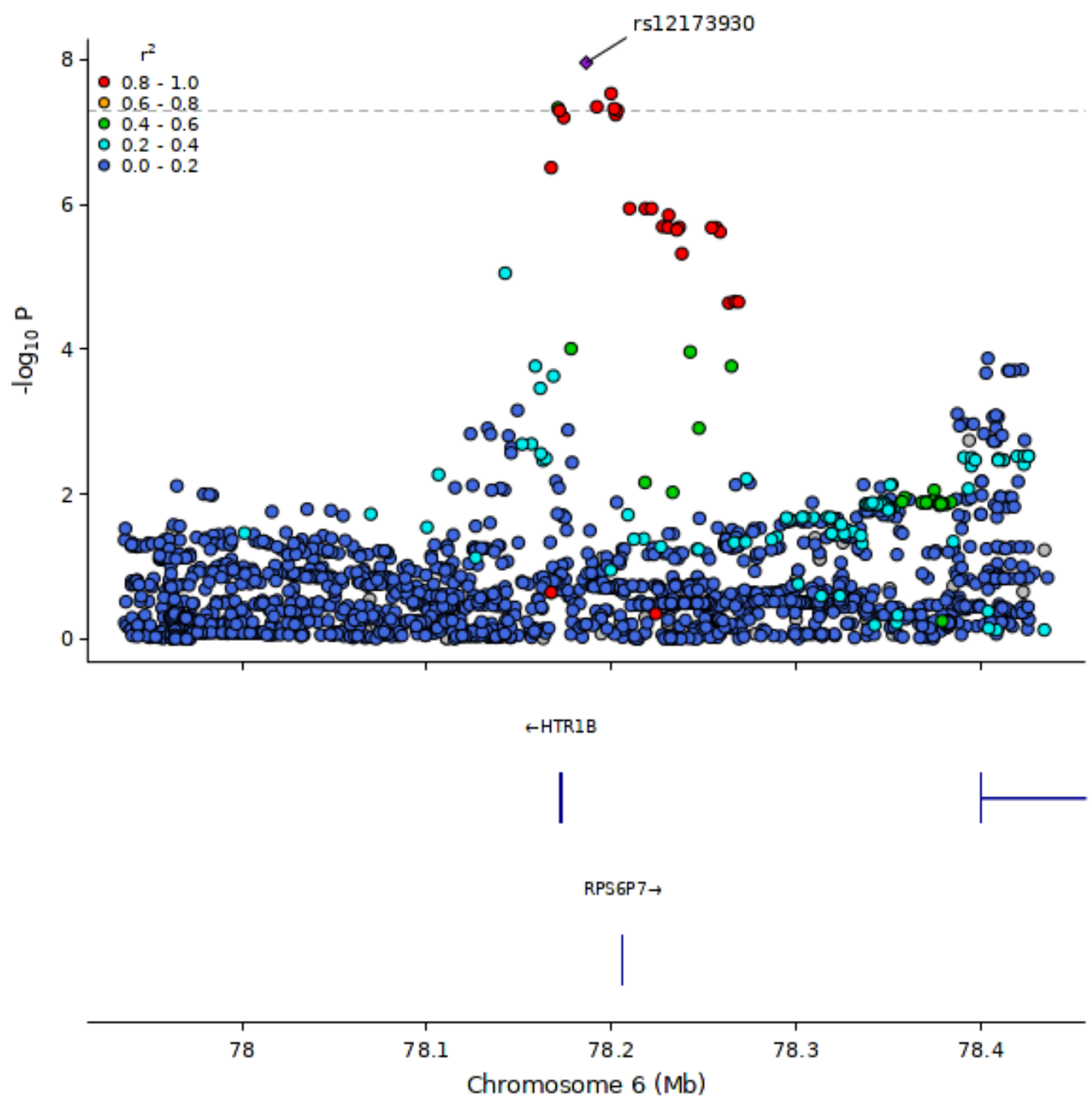

Supplementary Fig. 3 | Regional plots for the two lead independent SNPs associated with cross-age Shyness. Association p-values are given on the y-axis (-$\log_{10}$[p-value]), chromosomal position is given on the x-axis. Each lead SNP is indicated by a purple diamond, and other SNPs are colour-coded according to their LD with the lead SNP, using the 1000 Genomes[1] European reference panel. Gene positions within the locus are shown along the bottom of each plot.

4. Regional plots for the independent genome-wide significant loci associated with specific age temperament traits

rs3130985 (Chr6: 31085356)

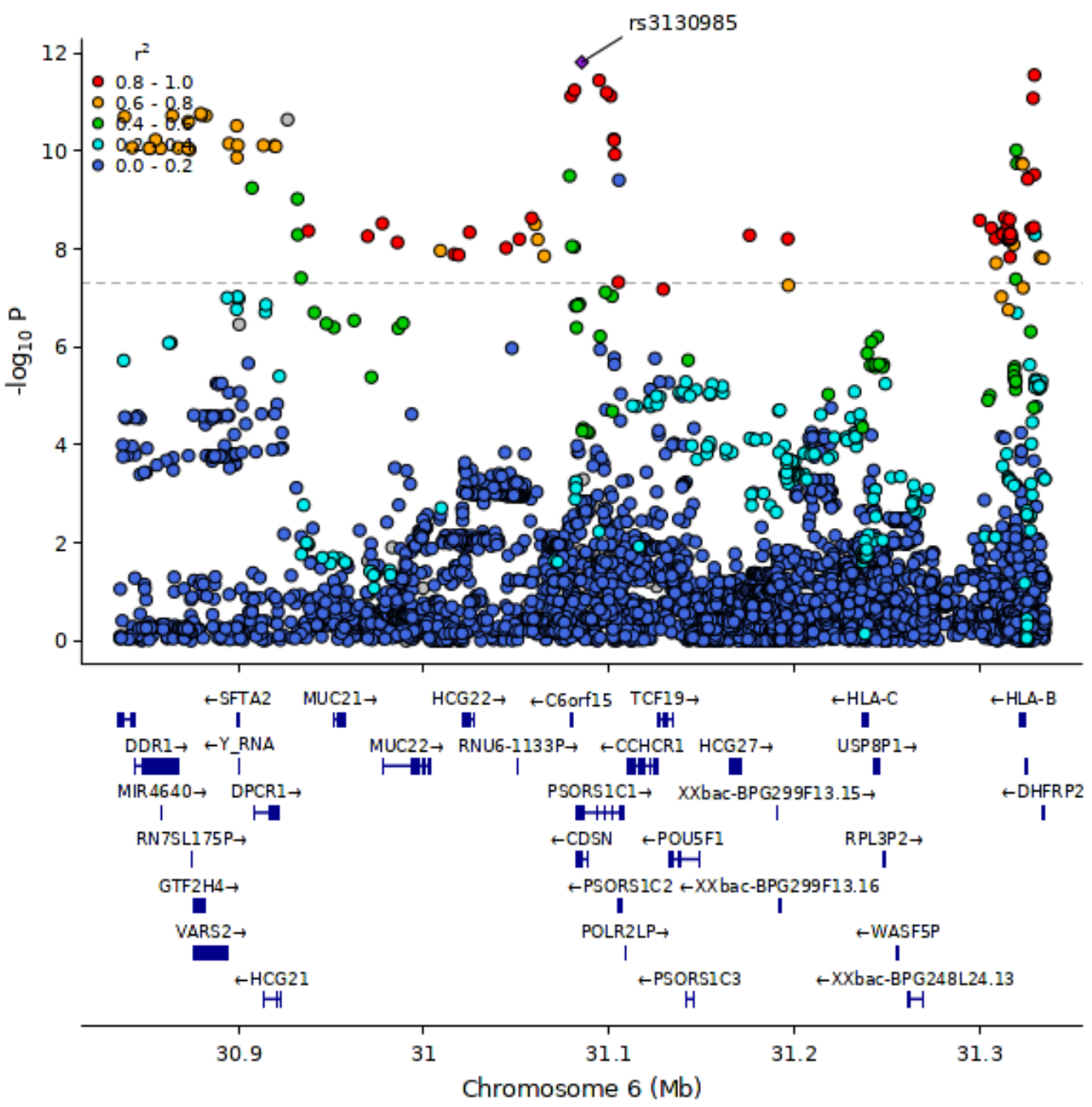

rs1054442 (Chr12: 49389320)

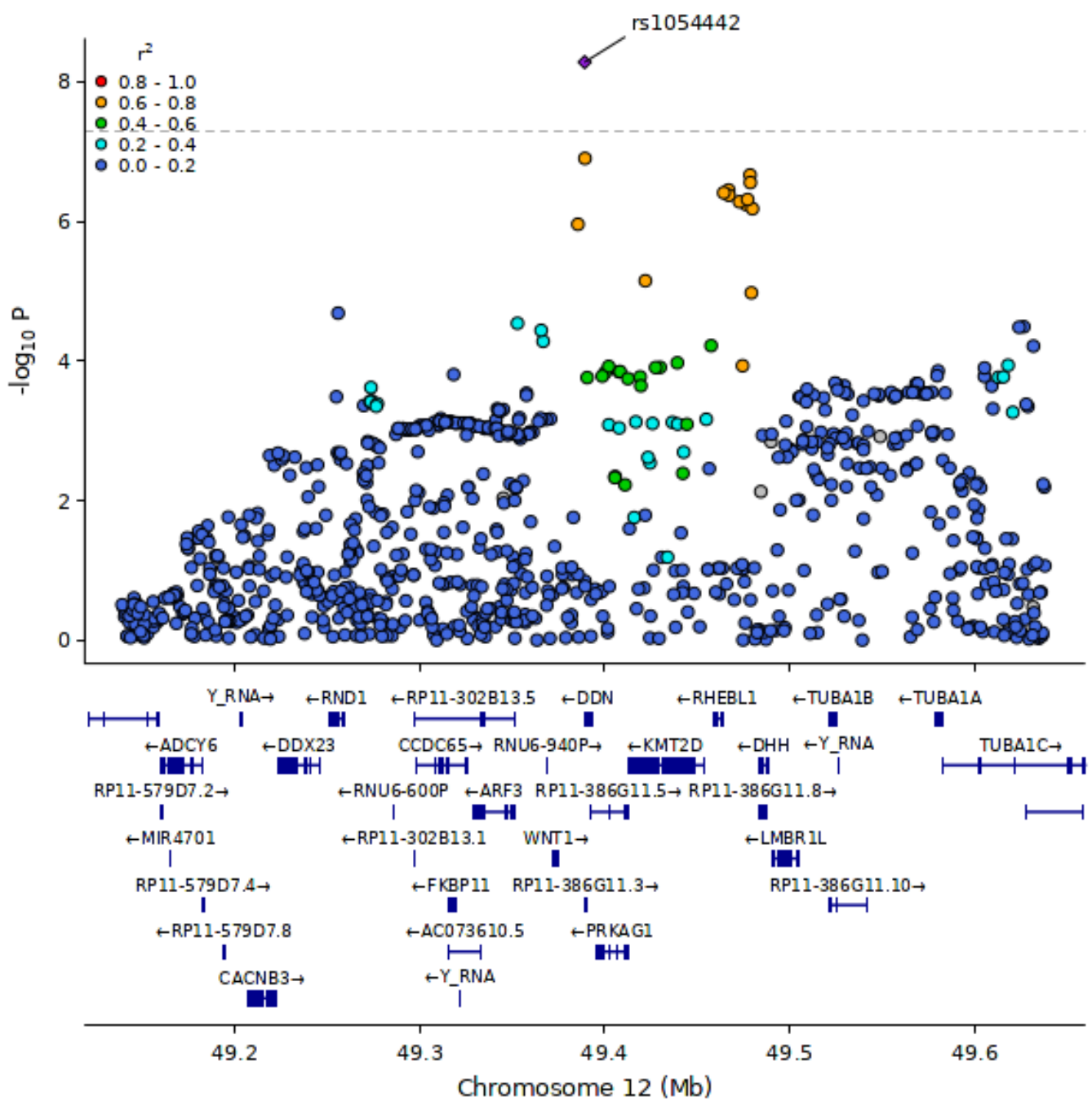

rs2416049 (Chr14: 47801869)

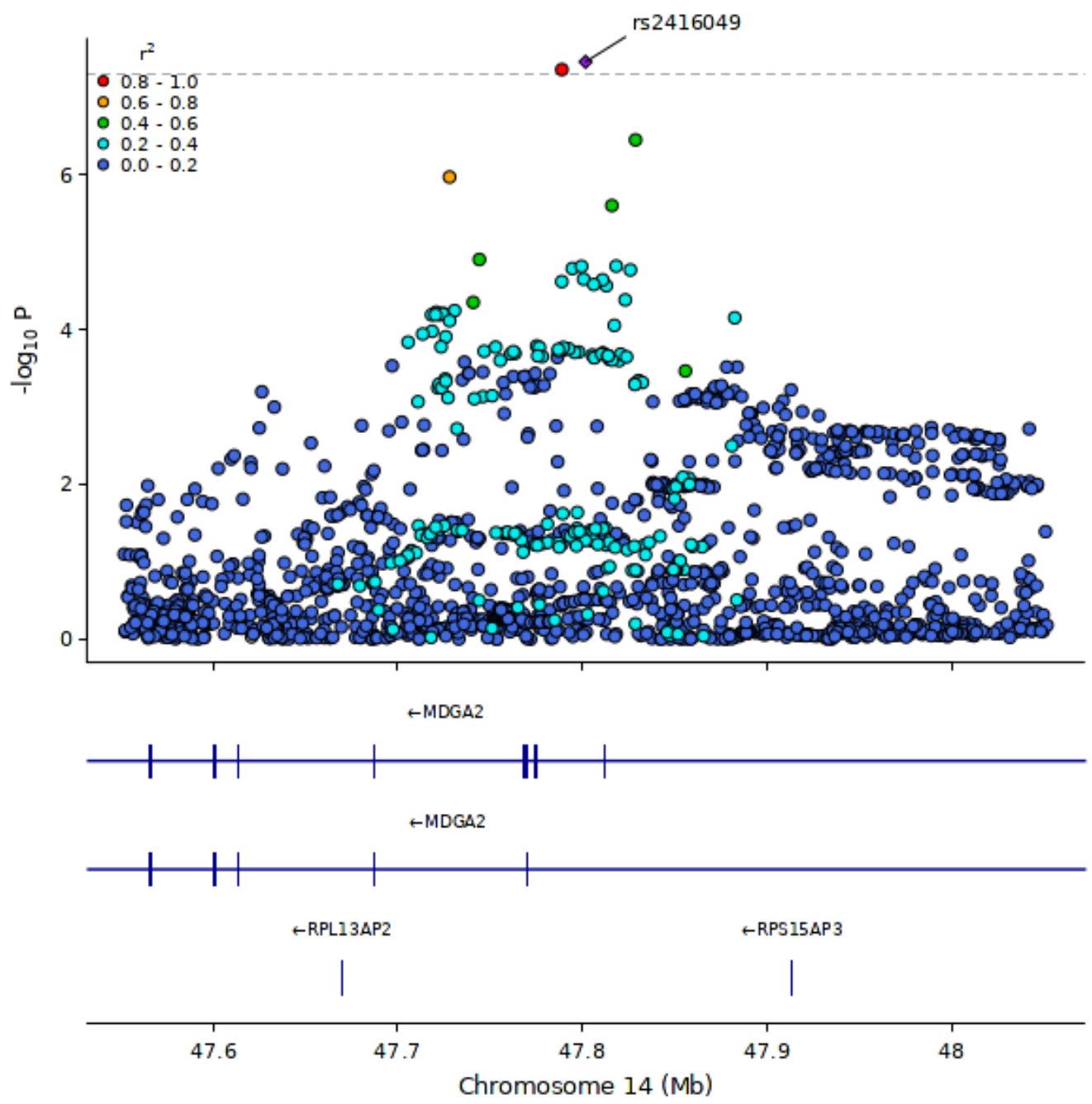

Supplementary Fig. 4 | Regional plots for the lead independent SNPs associated with second year Activity (upper left), third year Activity (upper right) and second year Shyness (lower panel). Association p-values are given on the y-axis ($-\log_{10}$[p-value]), chromosomal position is given on the x-axis. Each lead SNP is indicated by a purple diamond, and other SNPs are colour-coded according to their LD with the lead SNP, using the 1000 Genomes[1] European reference panel. Gene positions within the locus are shown along the bottom of each plot.

5. Manhattan and QQ plots for GWAS meta-analyses of cross-age infant temperament traits

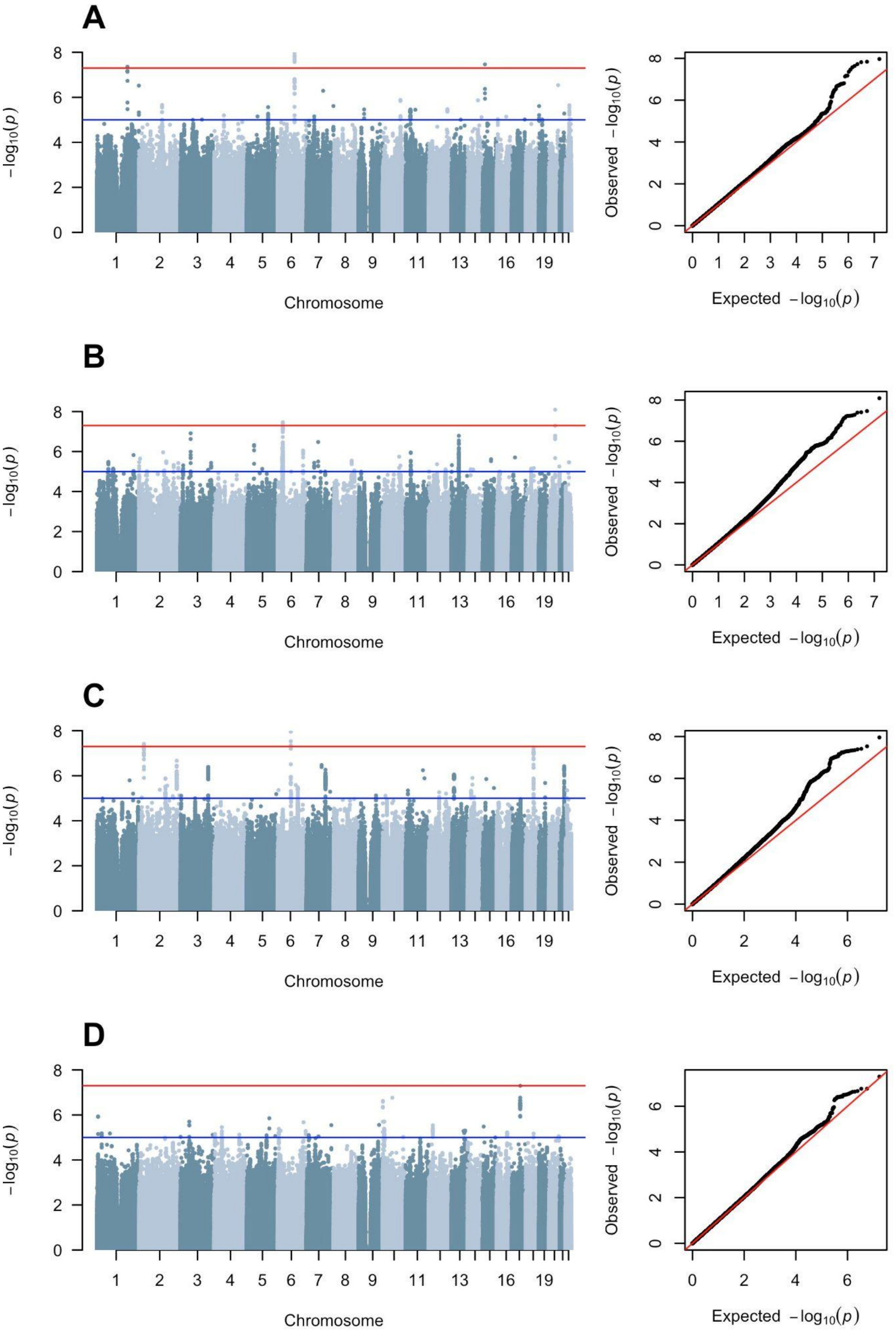

Supplementary Fig. 5 | Manhattan (left) and quantile-quantile (QQ; right) plots for each of the GWAS of cross-age temperament. These plots are for analyses of (A) Emotionality N=50,426, (B) Activity N=50,453, (C) Shyness N=45,656 and (D) Sociability N=43,963 , all cross-age. Manhattan plots show the -$\log_{10}$p-value (y-axis) for the association between each of the genetic variants included in the meta-analysis and the phenotype. P-values (two-tailed) are from the standard-error weighted fixed-effects meta-analysis in METAL[2] and are not adjusted for multiple comparisons,. The horizontal red line indicates genome-wide significant threshold ($p=5x10^{-8}$), the blue line indicates suggestive significance threshold ($p=1x10^{-5}$). Variants are ordered by chromosome and base pair location (x-axis). QQ plots show the same -$\log_{10}$p-values against the expected -$\log_{10}$p-values (red line).

6. Manhattan and QQ plots for GWAS meta-analyses of temperament traits in the second year

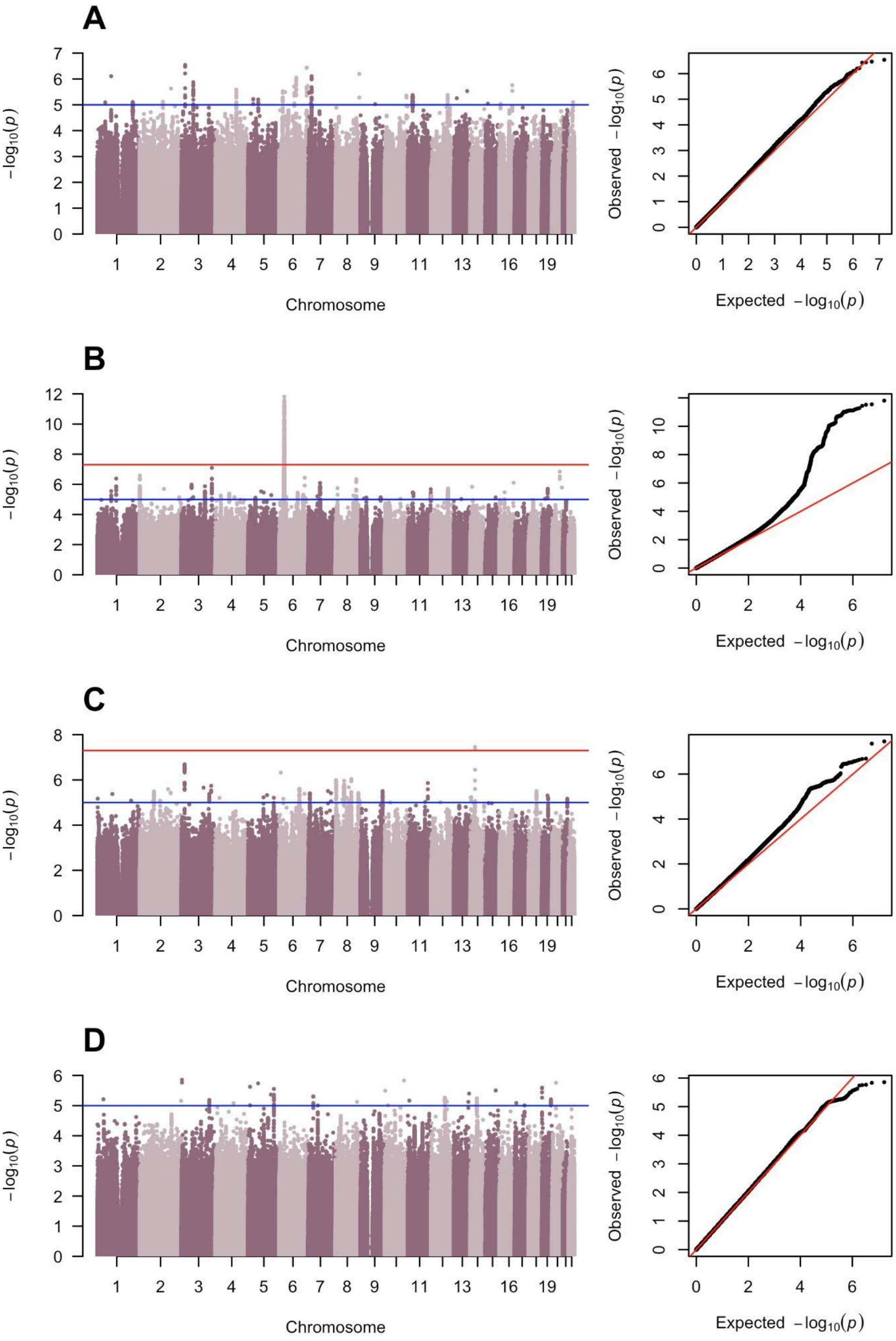

Supplementary Fig. 6 | Manhattan (left) and quantile-quantile (QQ; right) plots for each of the GWAS of temperament in the second year. These plots are for analyses of (A) Emotionality N=69,194, (B) Activity N=72,663, (C) Shyness N=63,663 and (D) Sociability N=62,230, in the second year. Manhattan plots show the $-\log_{10}$p-value (y-axis) for the association between each of the genetic variants included in the meta-analysis and the phenotype. P-values (two-tailed) are from the standard-error weighted fixed-effects meta-analysis in METAL[2] and are not adjusted for multiple comparisons. The horizontal red line indicates genome-wide significant threshold ($p=5\times10^{-8}$), the blue line indicates suggestive significance threshold ($p=1\times10^{-5}$). Variants are ordered by chromosome and base pair location (x-axis). QQ plots show the same $-\log_{10}$p-values against the expected $-\log_{10}$p-values (red line).

7. Manhattan and QQ plots for GWAS meta-analyses of temperament traits in the third year

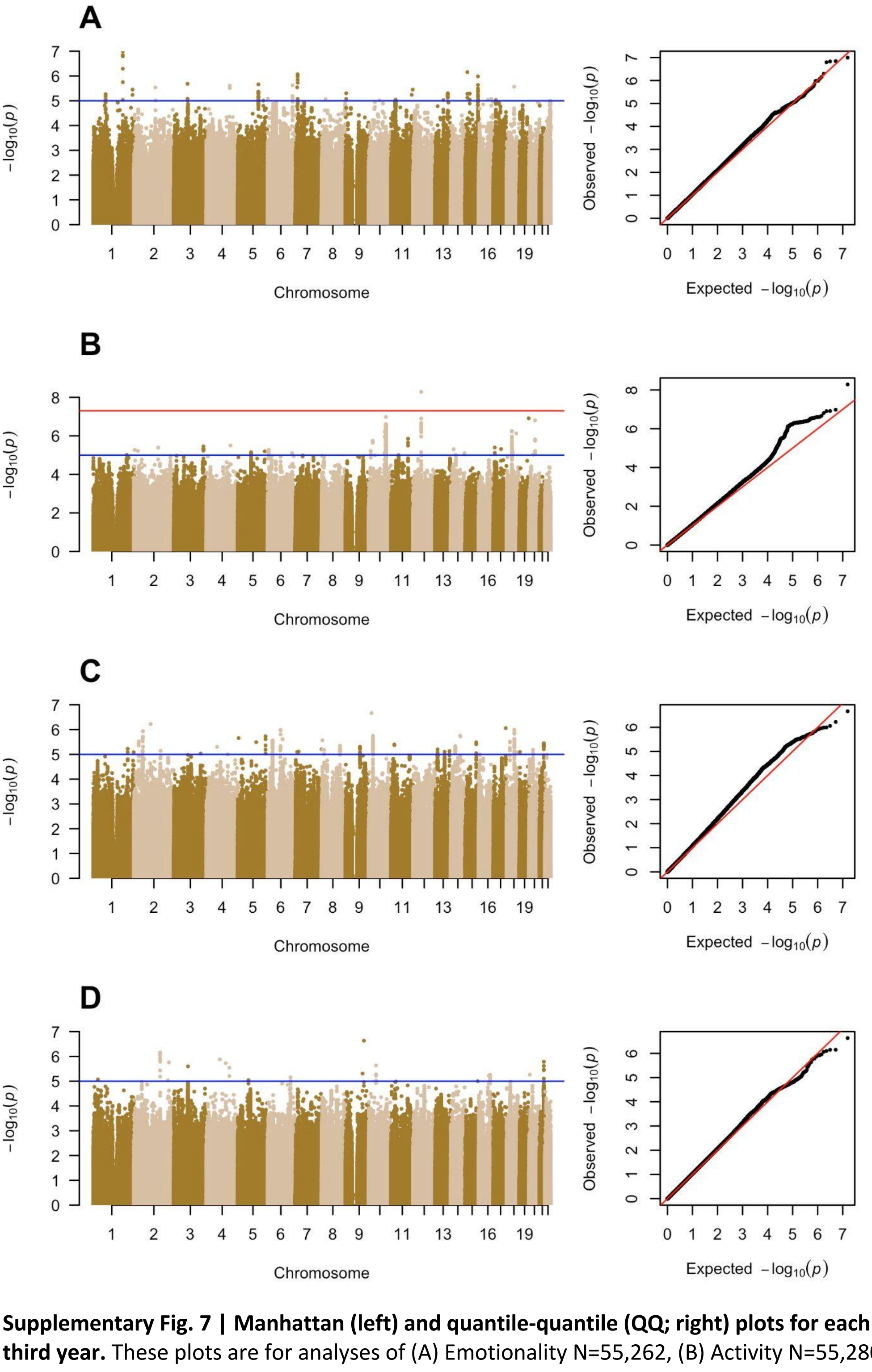

Supplementary Fig. 7 | Manhattan (left) and quantile-quantile (QQ; right) plots for each of the GWAS of temperament in the third year. These plots are for analyses of (A) Emotionality N=55,262, (B) Activity N=55,280, (C) Shyness N=55252 and (D) Sociability N=55,240, in the third year. Manhattan plots show the $-\log_{10}$p-value (y-axis) for the association between each of the genetic variants included in the meta-analysis and the phenotype. P-values (two-tailed) are from the standard-error weighted fixed-effects meta-analysis in METAL[2] and are not adjusted for multiple comparisons. The horizontal red line indicates genome-wide significant threshold ($p=5x10^{-8}$), the blue line indicates suggestive significance threshold ($p=1x10^{-5}$). Variants are ordered by chromosome and base pair location (x-axis). QQ plots show the same $-\log_{10}$p-values against the expected $-\log_{10}$p-values (red line).

8. Enrichment of up- and down-regulated DEGs for Activity in the second year and third year

For the FUMA-identified genes from Activity in the second year (N=163 mapped to the significant SNPs), 24 (Bonferroni-adjusted p-value for enrichment = 0.041) were in upregulated Differentially Expressed Genes (DEGs) set in whole blood tissue type, out of 54 specific tissue types analysed. There was also significant downregulation of the DEGs in Breast Mammary Tissue amongst the FUMA-identified genes for Activity in the third year (Supplementary Fig. S4)

All phenotypes (Emotionality, Activity, Shyness and Sociability) were analysed using FUMA, however results from the analysis of the enrichment of up- and down-regulated DEGS only yielded significant results for Activity in the second and third years. As such, figures from only these two are provided below.

A

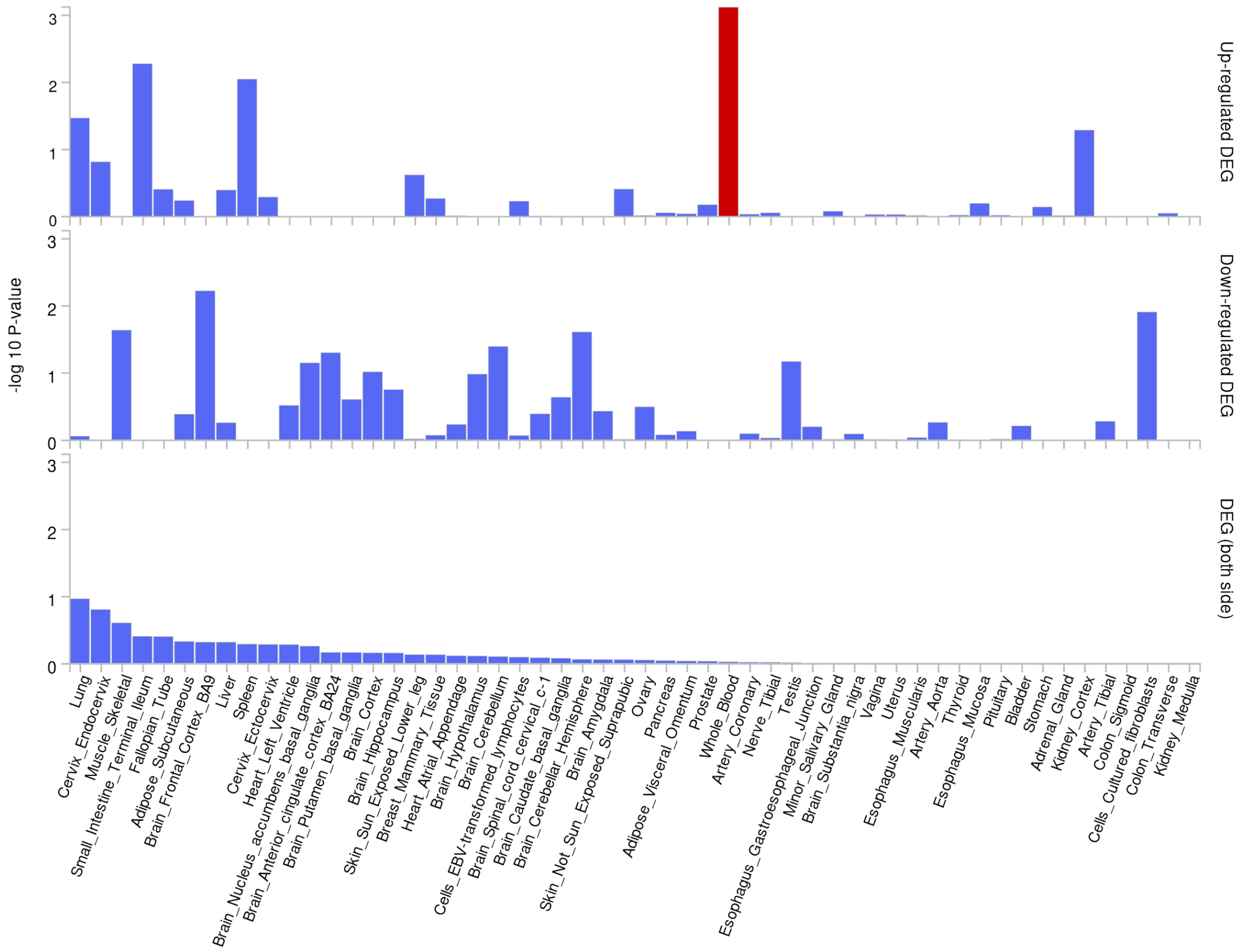

B

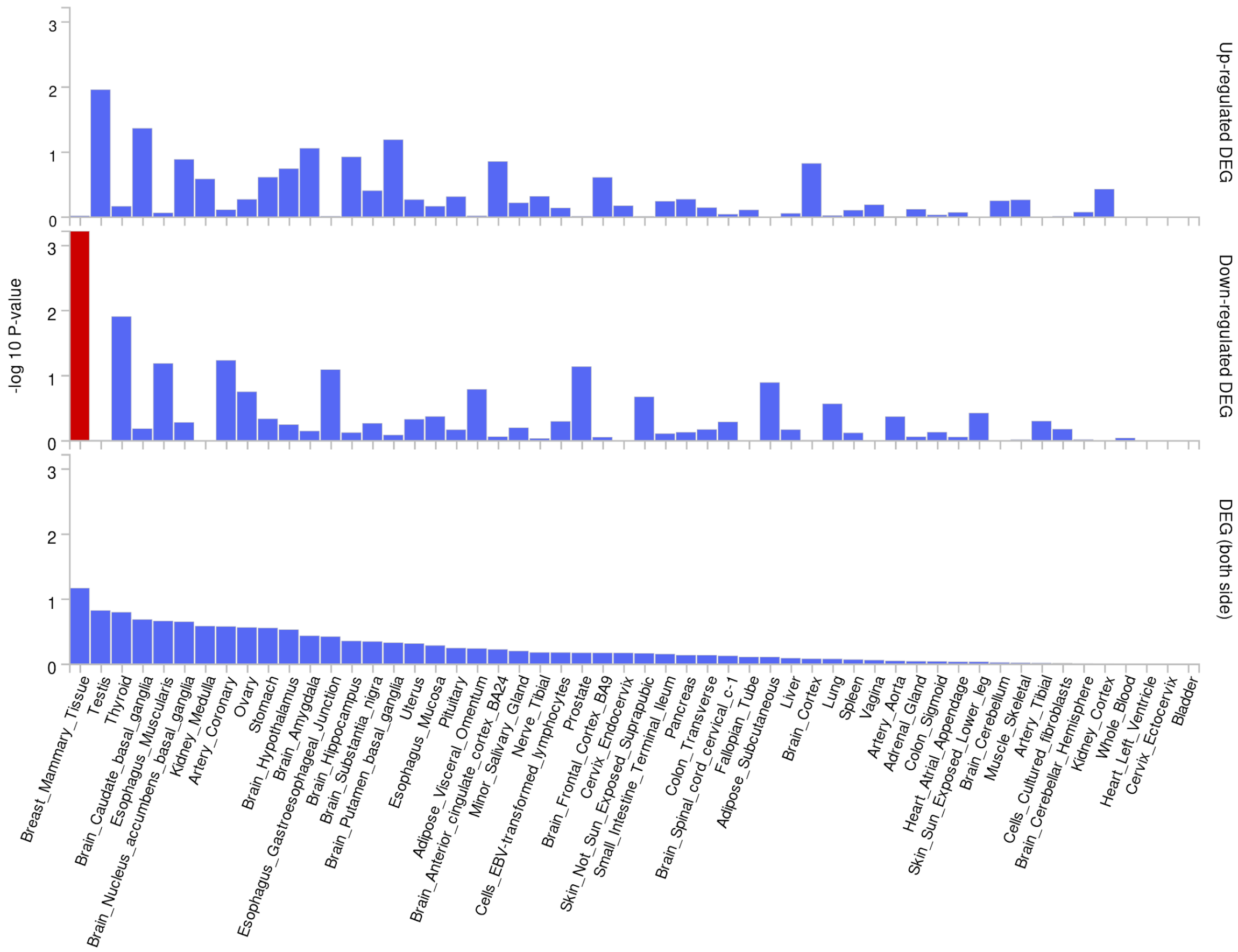

Supplementary Fig. 8 | Enrichment of up- and down-regulated Differentially Expressed Genes (DEGs). Plots are shown for Activity in the second year (A) and third year (B). This was conducted in FUMA[3] using 54 GTEx v8 tissues (listed along the x-axis) with the -$\log_{10}$p-value (one-sided) of the hypergeometric test performed within the GENE2FUNC analyses in FUMA along the y-axis. Red bars indicate that this test was significant at a Bonferroni-corrected p-value ≤ 0.05.

9. MAGMA tissue expression analysis for cross-age Shyness

There was significantly enriched expression of the genes associated with Shyness in hypothalamus and pituitary gland, as identified by MAGMA[4] GTEx[5] tissue expression analysis. There was no significant enrichment of the genes associated with other temperament traits in any specific tissues from MAGMA GTEx tissue expression analysis.

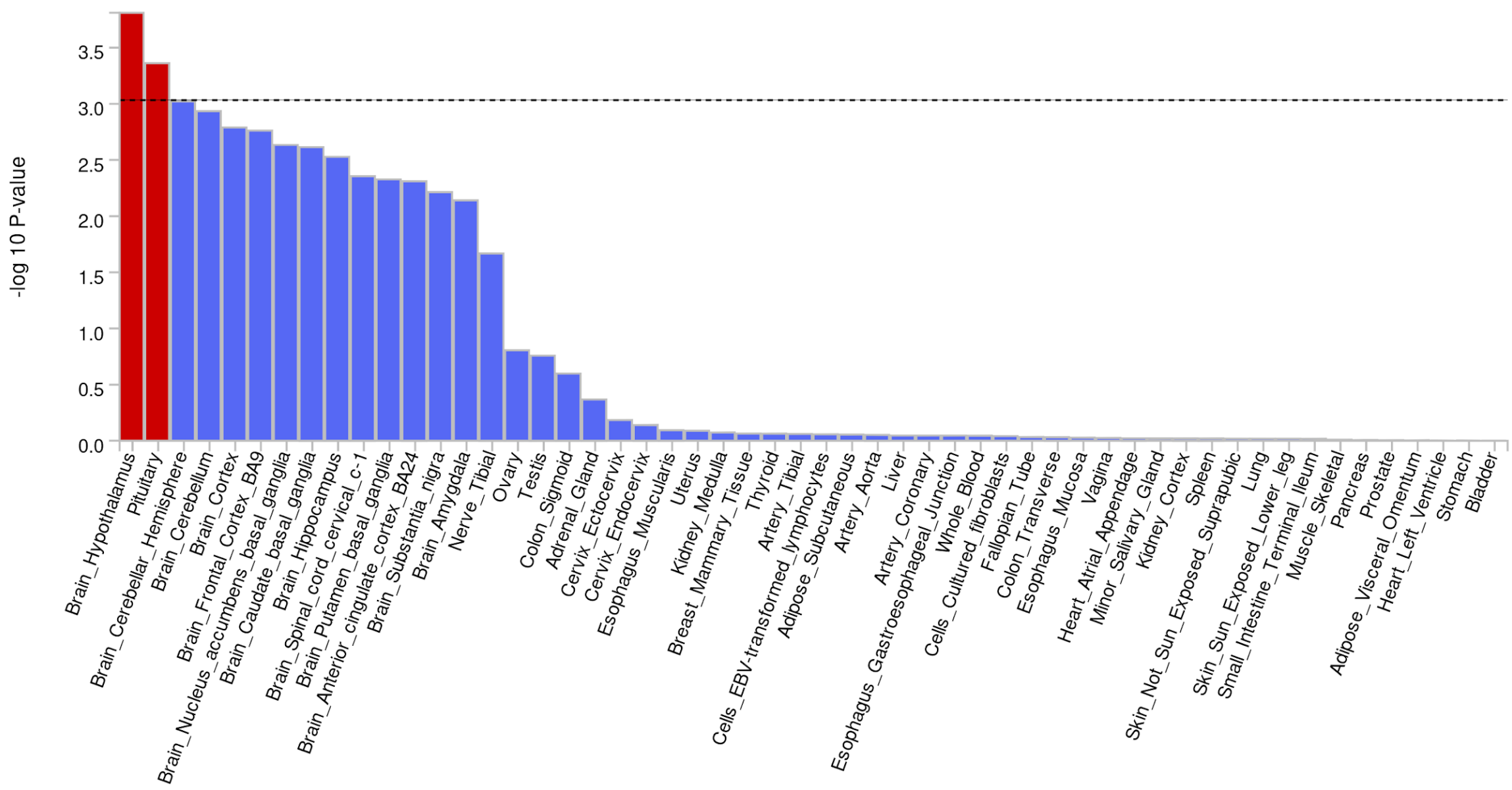

Supplementary Fig. 9 | MAGMA gene property analysis of cross-age shyness using GTEx v8 54 specific tissue types. The y-axis represents the one-sided -$\log_{10}$p-values of the regression model testing for a positive association between tissue specificity of the genes for the 54 GTEx v8 tissue types (x-axis) and the gene associations with cross-age Shyness. Bars are red when the p-value is less than the Bonferroni corrected threshold of $p=9\times10^{-4}$ (0.05/54 tissue types) or -$\log_{10}$p-value = 3.046.

10. eQTL plot for colocalisation of rs1054442 in GWAS of activity in the third year and *RHEBL1*

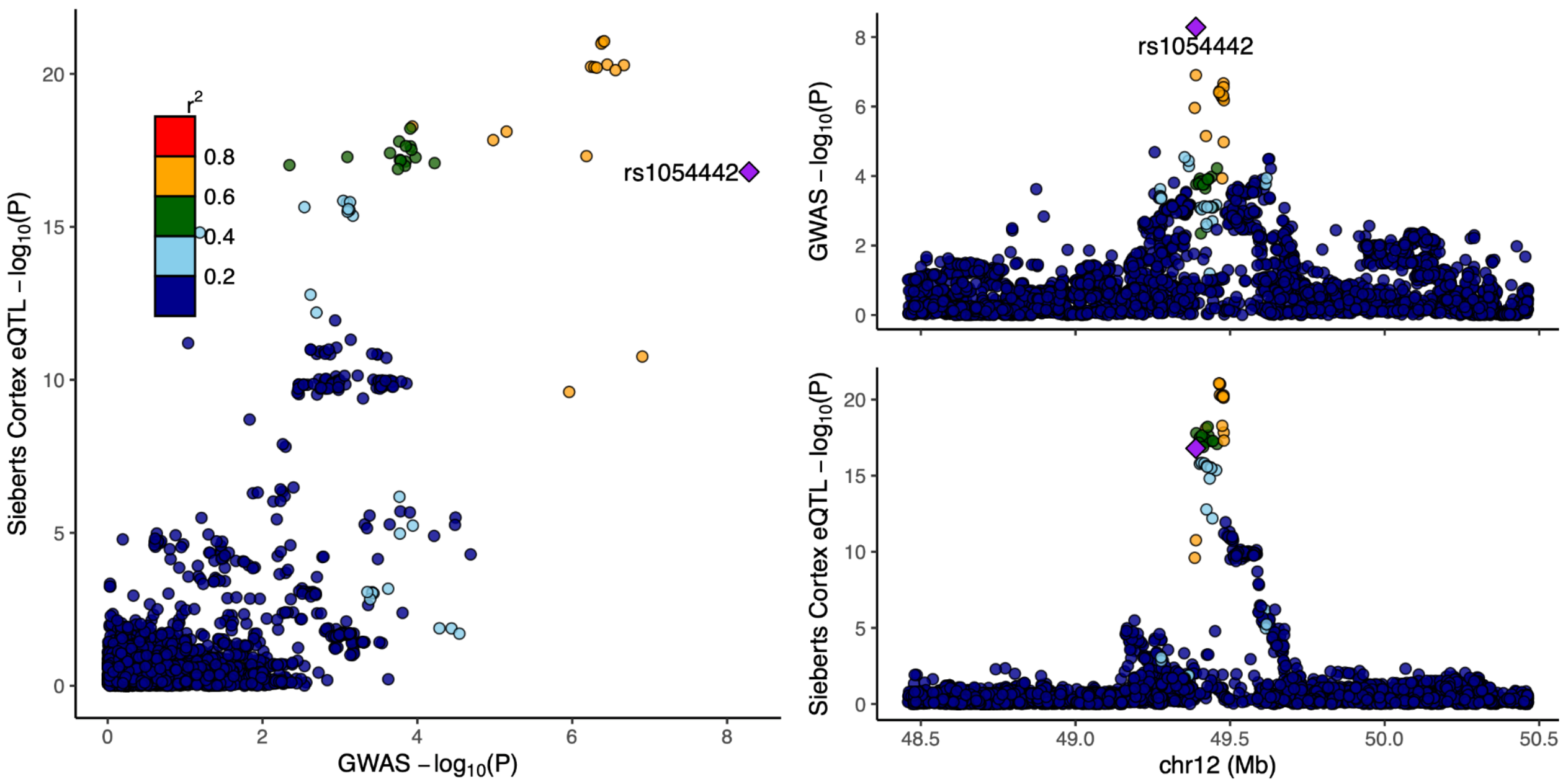

Supplementary Fig. 10 | eQTL association plot for rs1054442 in the GWAS of activity in the third year. The left plot shows an association between the -log10[p-value](x-axis) of the GWAS of activity in the third year and the -log10[p-values](y-axis) for the eQTL for *RHEBL1* on chromosome 12. Plots on the right show -log10[p-values] (y-axes) for the GWAS of activity in the third year (top) and the eQTL for *RHEBL1* (bottom) within a 2Mb window around the lead SNP, rs1054442 (purple diamond). P-values were not corrected for multiple comparisons. Points are coloured according to their linkage disequilibrium (LD) correlation with the lead SNP (rs1054442); LD was estimated in individuals with European Ancestry in the 1000 Genomes dataset[1].

11. Overlap between significant lead SNP for activity in the third year and lead SNPs for educational attainment and cognitive performance

A

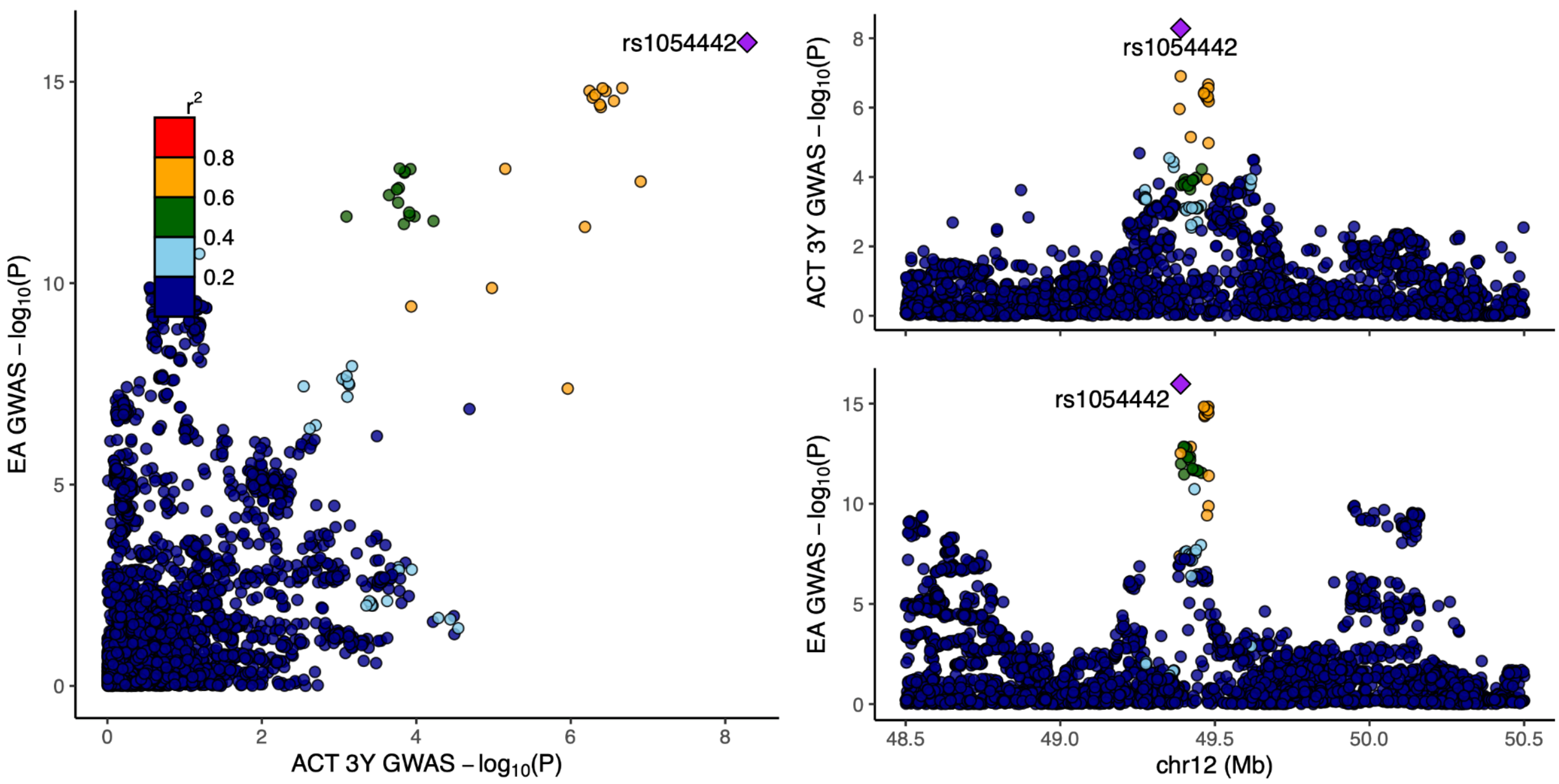

B

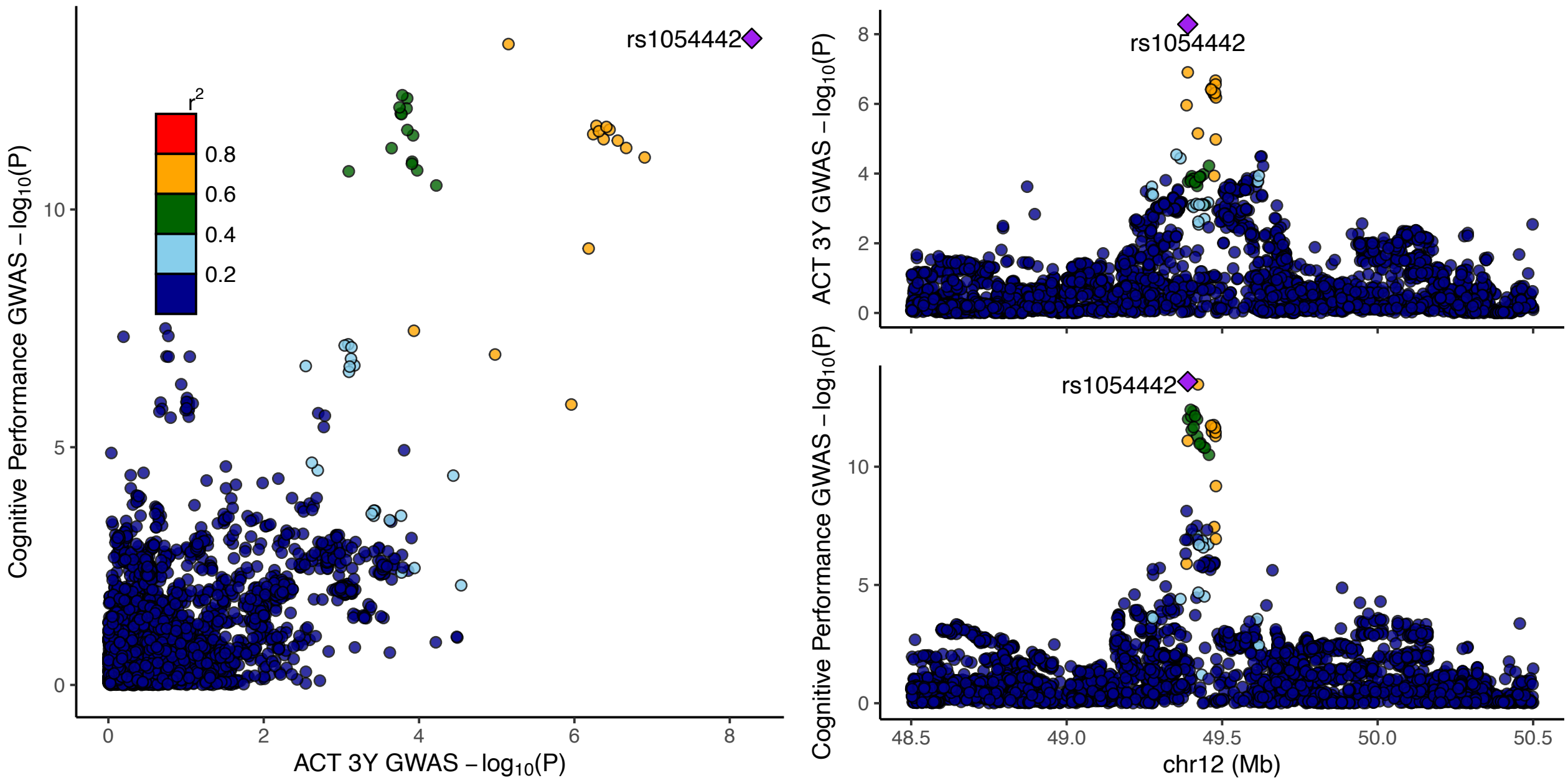

Supplementary Fig. 11 | Overlap between the lead SNP on chromosome 12 in the GWAS of activity in the third year (rs1054442) and the same lead SNP from GWAS of (A) educational attainment (EA; Lee et al., 2018) and (B) cognitive performance (Savage et al., 2018). For each of the panels (A and B), the left plot shows the -log10[p-values] of the GWAS of activity in the third year (x-axis) against the -log10[p-values] of the GWAS of (A) EA and (B) cognitive performance (y-axis). Plots on the right show the -log10[p-values] of each of these two GWAS within 2Mb locus about the lead SNP (rs1054442, purple diamond) identified in both GWAS. These are for activity in the third year (top) and (A) EA and (B) cognitive performance (bottom). Points are coloured according to their linkage disequilibrium (LD) correlation with the lead SNP; LD was estimated in individuals with European Ancestry in the 1000 Genomes dataset[1]. P-values were not corrected for multiple comparisons.

12. eQTL plot for colocalisation of rs10797666 in GWAS of cross-age emotionality and *MR1*

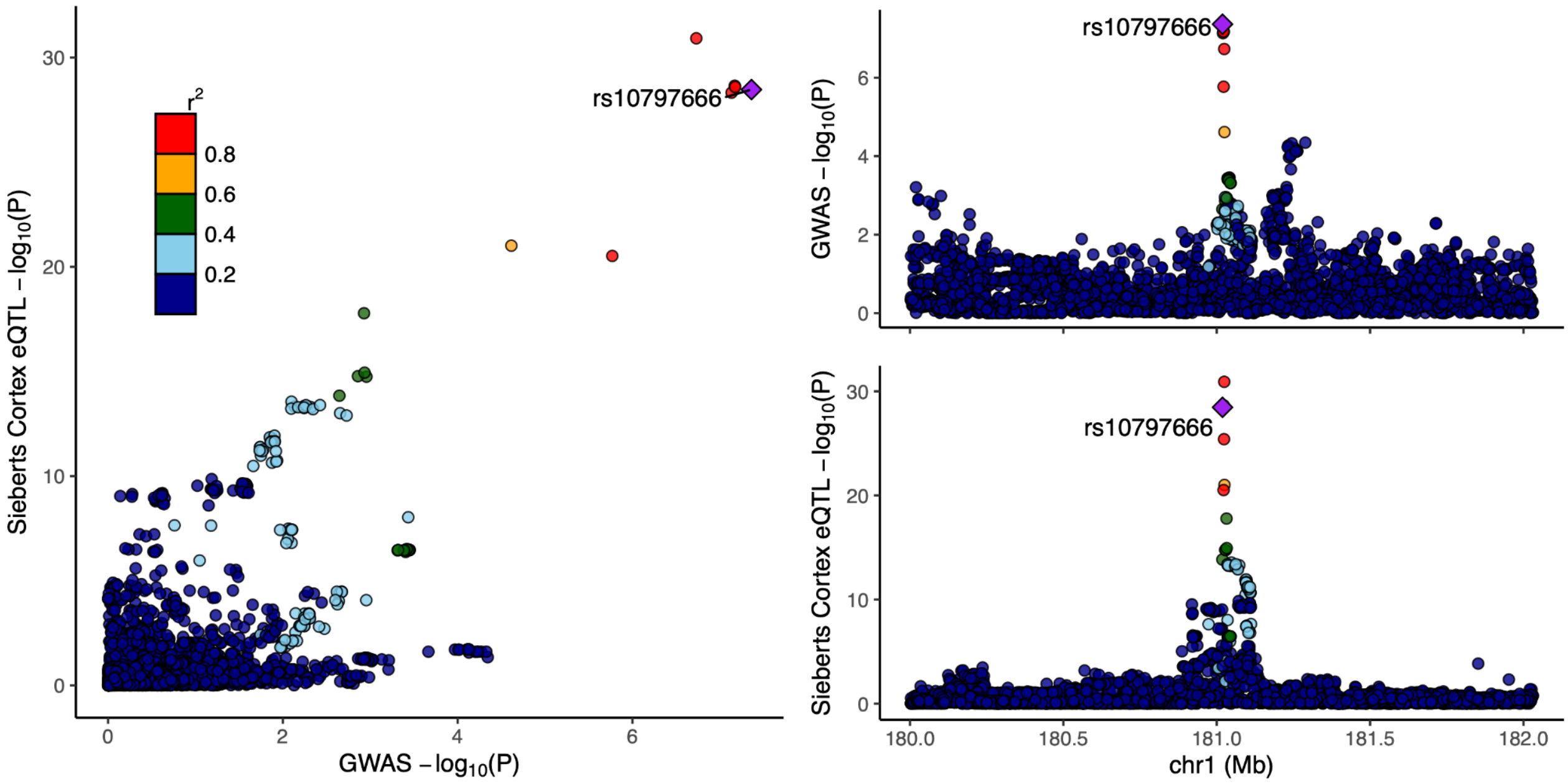

Supplementary Fig. 12 | eQTL association plot for rs10797666 in the GWAS of cross-age emotionality. The left plot shows an association between the -log10[p-value](x-axis) of the GWAS of cross-age emotionality and the -log10[p-values](y-axis) for the eQTL for *MR1* on chromosome 1. Plots on the right show -log10[p-values] (y-axes) for the GWAS of cross-age emotionality (top) and the eQTL for *MR1* (bottom) within a 2Mb window around the lead SNP, rs10797666 (purple diamond). P-values were not corrected for multiple comparisons. Points are coloured according to their linkage disequilibrium (LD) correlation with the lead SNP (rs10797666); LD was estimated in individuals with European Ancestry in the 1000 Genomes dataset[1].

13. Inter-temperament genetic correlations for the second year and third year in infancy

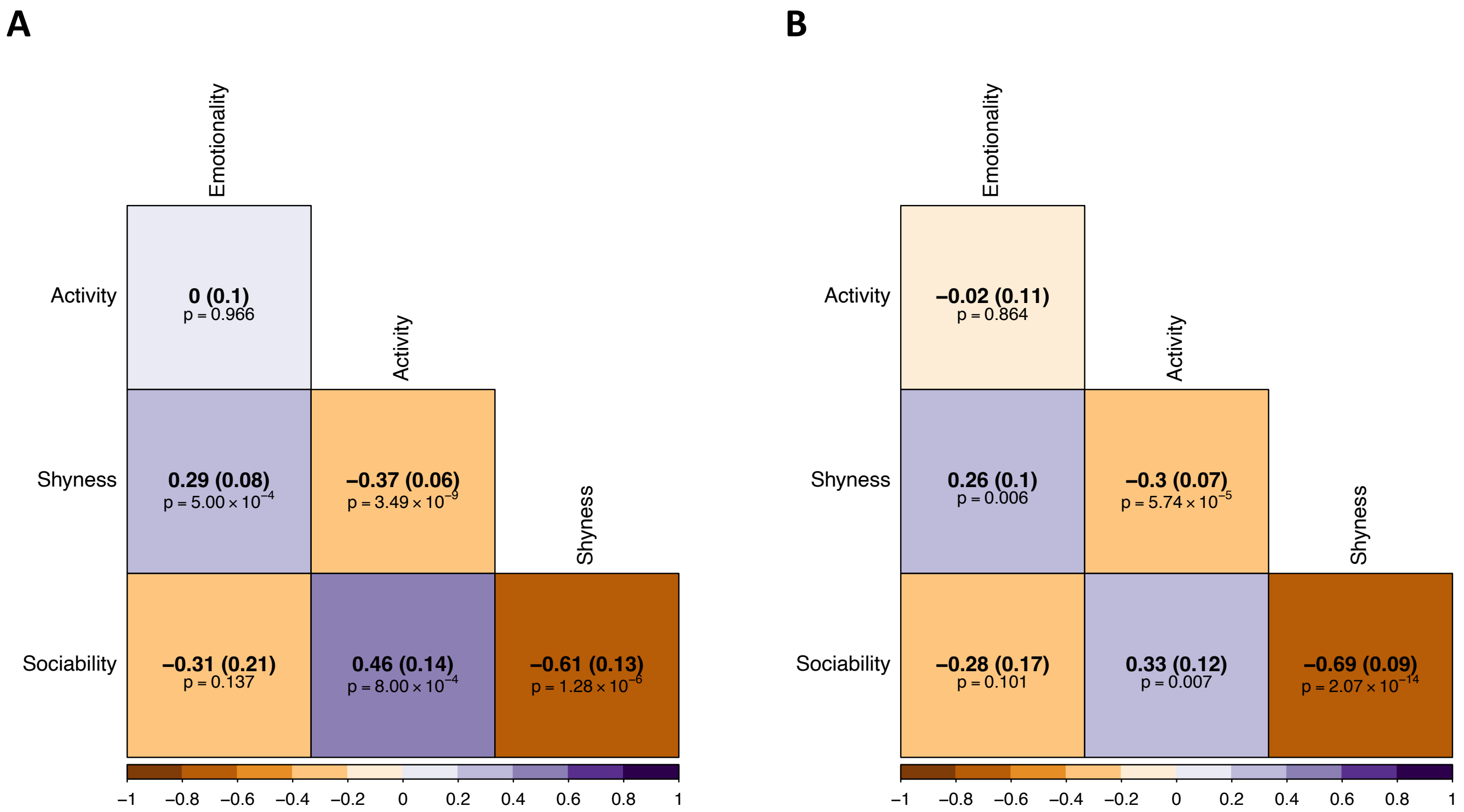

Supplementary Fig. 13 | Inter-temperament genetic correlations. LDSC[6] genetic correlations between each of the four second-year (A) and third year (B) temperament traits. Genetic correlation matrix shows the inter-temperament genetic correlations in bold with standard errors of the genetic correlation in parentheses and p-values (two-sided, not corrected for multiple comparisons) below. Each genetic correlation is colour-coded from dark orange (-1) to white (0) to dark purple (1) according to the magnitude of the genetic correlation. The GWAS sample sizes for each of the phenotypes included in these genetic correlation analyses were: Emotionality (second-year N = 69,194, third-year N = 55262); Activity (second-year N = 72,663, third-year N = 55280); Shyness (second-year N = 63,663, third-year N = 55,252); Sociability (second-year N = 62,230, third-year N = 55,240)

14. Polygenic score analysis for second-year temperament

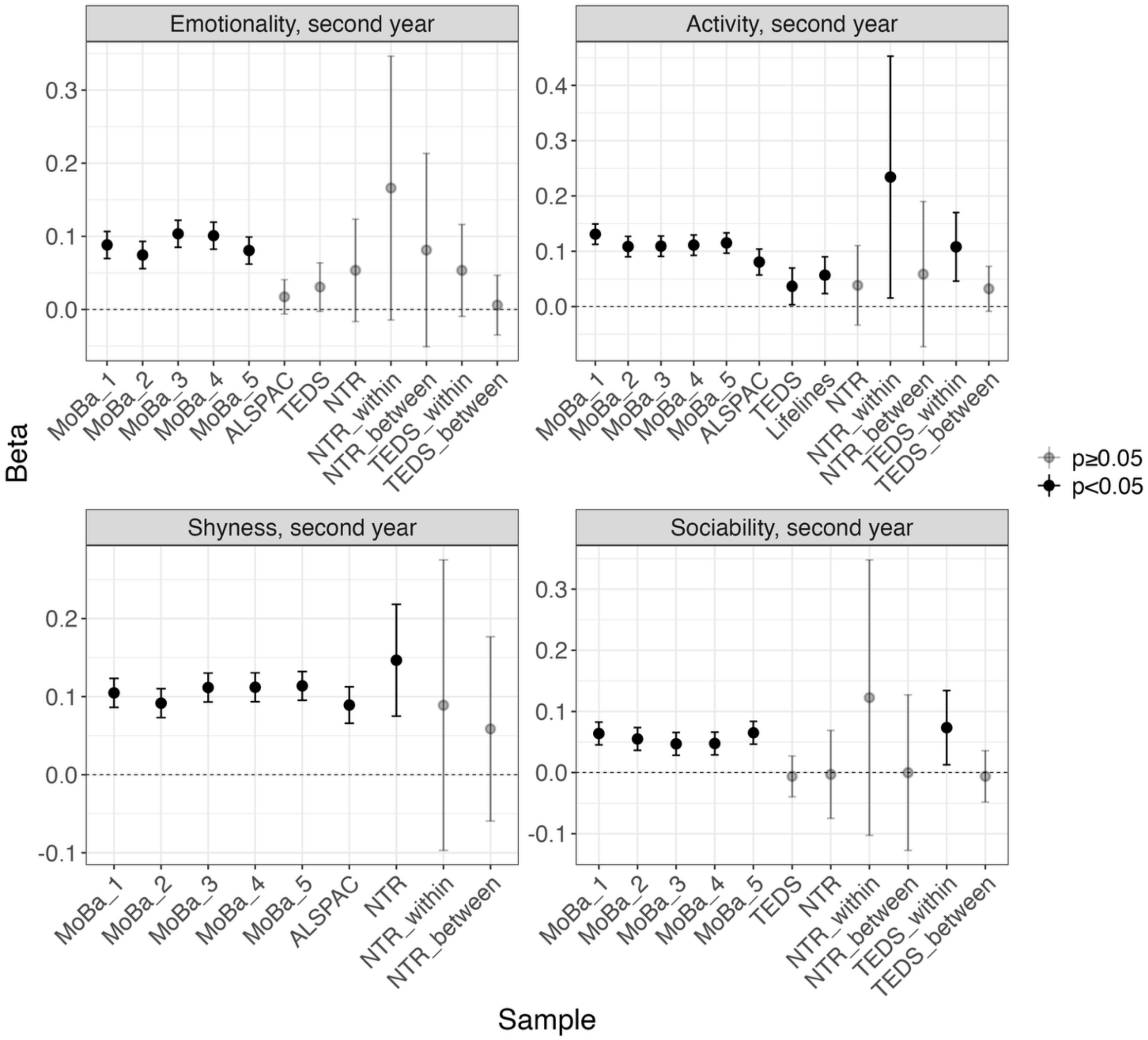

Supplementary Fig. 14 | Polygenic score (PGS) prediction estimates into target samples (second year). PGS standardised beta coefficients of the linear regression models testing for associations between the four second-year EAS temperament traits and their polygenic scores. The target samples were ALSPAC (Emotionality N=6,965, Activity N=6,966, Shyness N=6,955), TEDS (Emotionality N=3,473, Activity N=3,473, Sociability N=3,432), Lifelines (Activity N=3,435), NTR (Emotionality, Activity, Shyness and Sociability N=733) and MoBa_[1-5] (Emotionality sample 1 N=11,053 and samples 2-5 N=11,054; Activity samples 1-2 N=11,061 and samples 3-5 N=11,062; Shyness samples 1-5 N=11,055; Sociability samples 1-3 N=11,067 and samples 4-5 N=11,068). Target samples for within- and between-sib pair PGS analyses were NTR_[within/between] (Emotionality Npairs=193, Activity Npairs=193 , Shyness Npairs = 192, Sociability Npairs=193) and TEDS_[within/between] (Emotionality Npairs=1,743, Activity Npairs=1,738, Sociability Npairs=1,715). Standardised beta coefficients ± 95% CI (y-axis) are shown for each of the different analyses (x-axis). The points and error bars are dark grey when the p-value (two-tailed) for the beta-coefficient was $p<0.05$ and pale grey for $p\geq0.05$. P-values are uncorrected for multiple comparisons.

15. Polygenic score analysis for third-year temperament

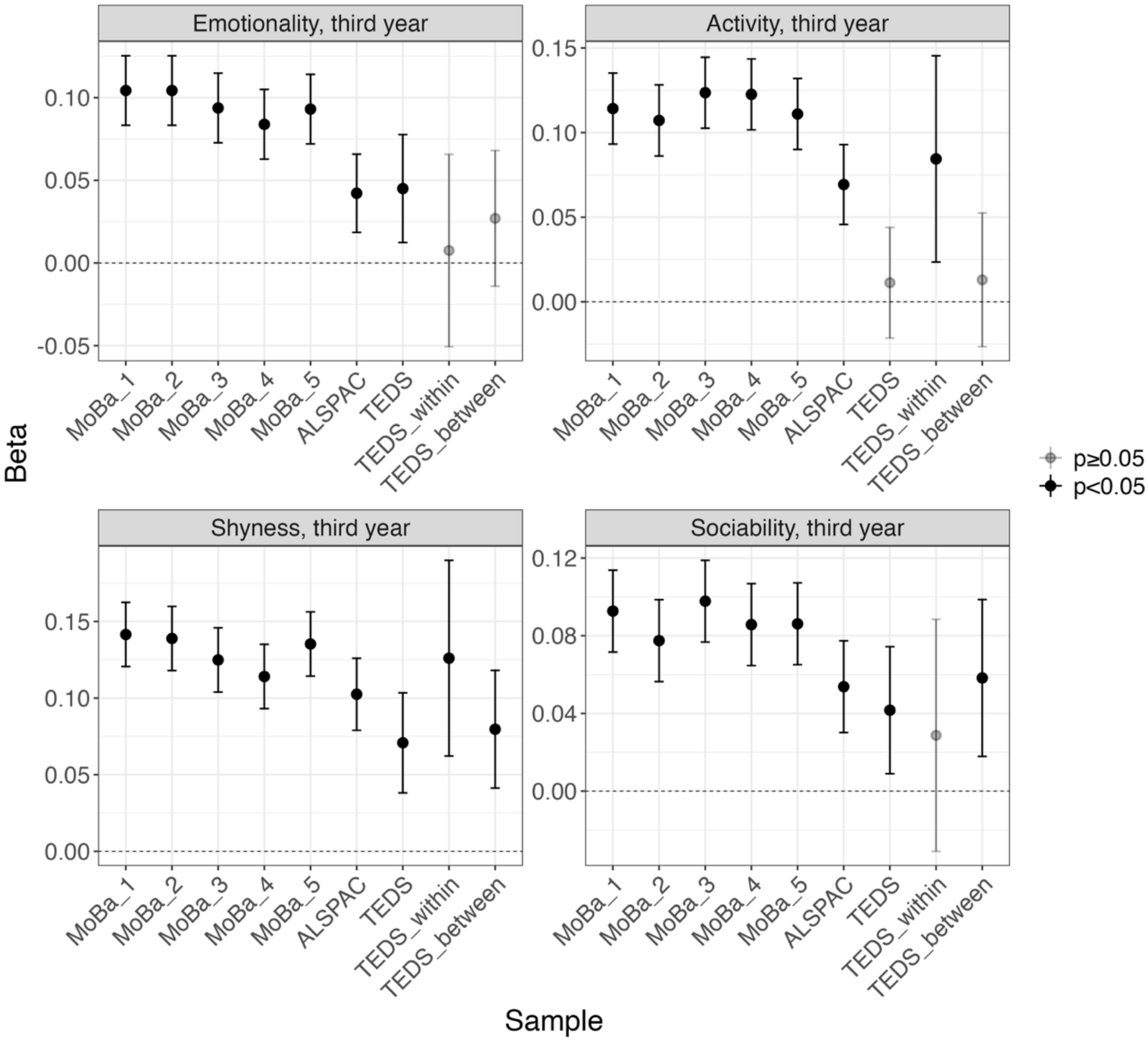

Supplementary Fig. 15 | Polygenic score (PGS) prediction estimates into target samples (third year). PGS standardised beta coefficients of the linear regression models testing for associations between the four third-year EAS temperament traits and their polygenic scores. The target samples were ALSPAC (Emotionality N=6,852, Activity N=6,855, Shyness N=6,853, Sociability N=6,850), TEDS (Emotionality N=3,583, Activity N=3,572, Shyness N=3,569, Sociability N=3,556) and MoBa_[1-5] (Emotionality sample 1 N=8,544 and samples 2-5 N=8,545; Activity sample 1 N=8,550 and samples 2-5 N=8,551; Shyness sample 1 N=8,546 and samples 2-5 N=8,547; Sociability samples 1-3 N=8,548 and samples 4-5 N=8,549).The target samples for within- and between-sib pair PGS analyses was TEDS_[within/between] (Emotionality Npairs=1,809, Activity Npairs=1,798 , Shyness Npairs = 1,796, Sociability Npairs=1,789). Standardised beta coefficients ± 95% CI (y-axis) are shown for each of the different analyses (x-axis). The points and error bars are dark grey when the p-value (two-tailed) for the beta-coefficient was $p<0.05$ and light grey for $p\geq0.05$. P-values are uncorrected for multiple comparisons.

16. Manhattan and QQ plots for multi-ancestry GWAS meta-analyses of temperament in the second year

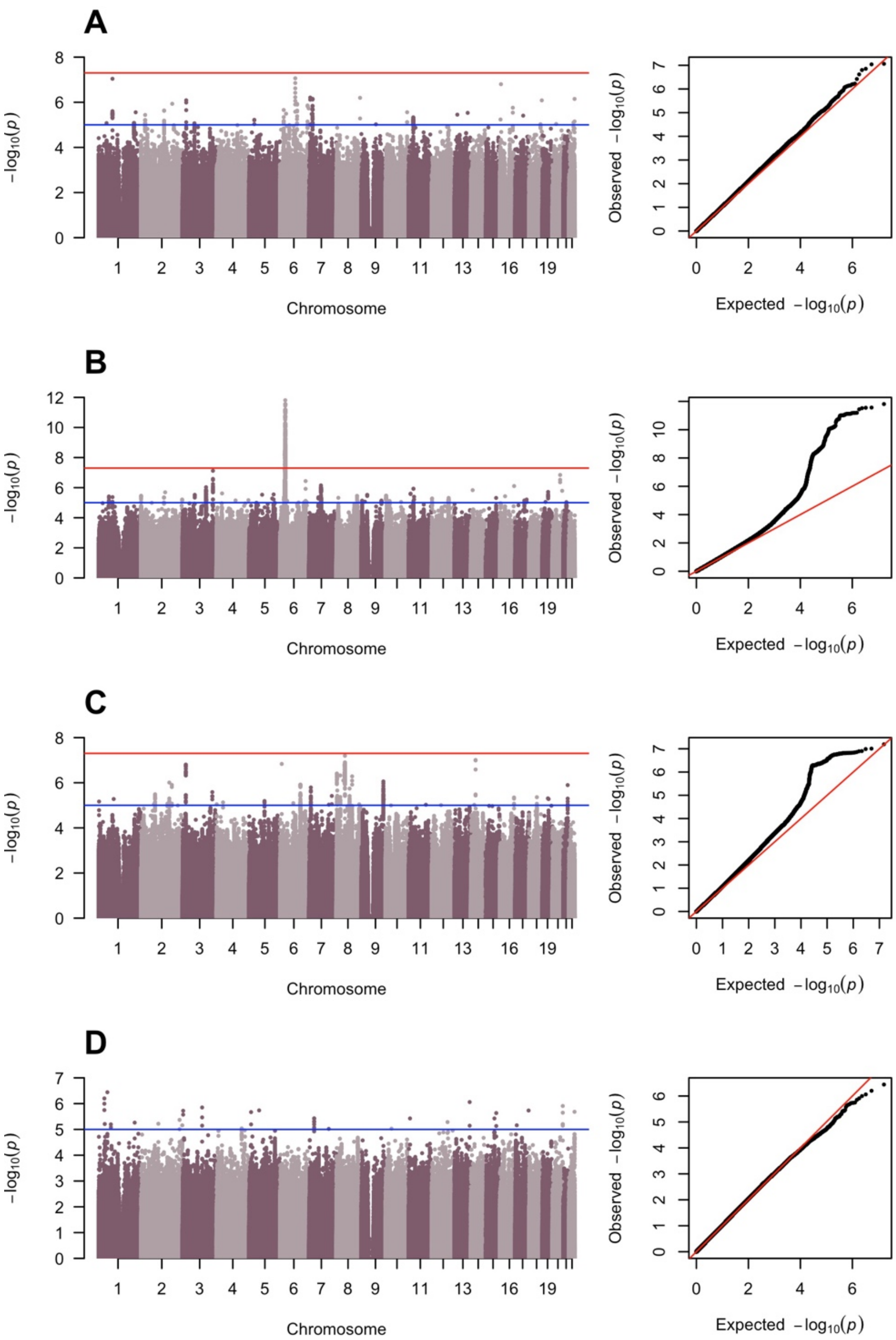

Supplementary Fig. 16 | Manhattan (left) and quantile-quantile (QQ; right) plots for multi-ancestry GWAS meta-analysis of temperament traits in the second year. These plots are for analyses of (A) Emotionality N=75,435, (B) Activity N=78,894, (C) Shyness N=69,877 and (D) Sociability N=68,478 in the second year. Manhattan plots show the -$\log_{10}$p-value (y-axis) for the association between each of the genetic variants included in the meta-analysis and the phenotype. P-values (two-tailed) are from the standard-error weighted fixed-effects meta-analysis in METAL[2] and are not adjusted for multiple comparisons. The horizontal red line indicates genome-wide significant threshold ($p=5x10^{-8}$), the blue line indicates suggestive significance threshold ($p=1x10^{-5}$). Variants are ordered by chromosome and base pair location (x-axis). QQ plots show the same -$\log_{10}$p-values against the expected -$\log_{10}$p-values (red line).

17. Manhattan and QQ plots for multi-ancestry GWAS meta-analyses of temperament in the third year

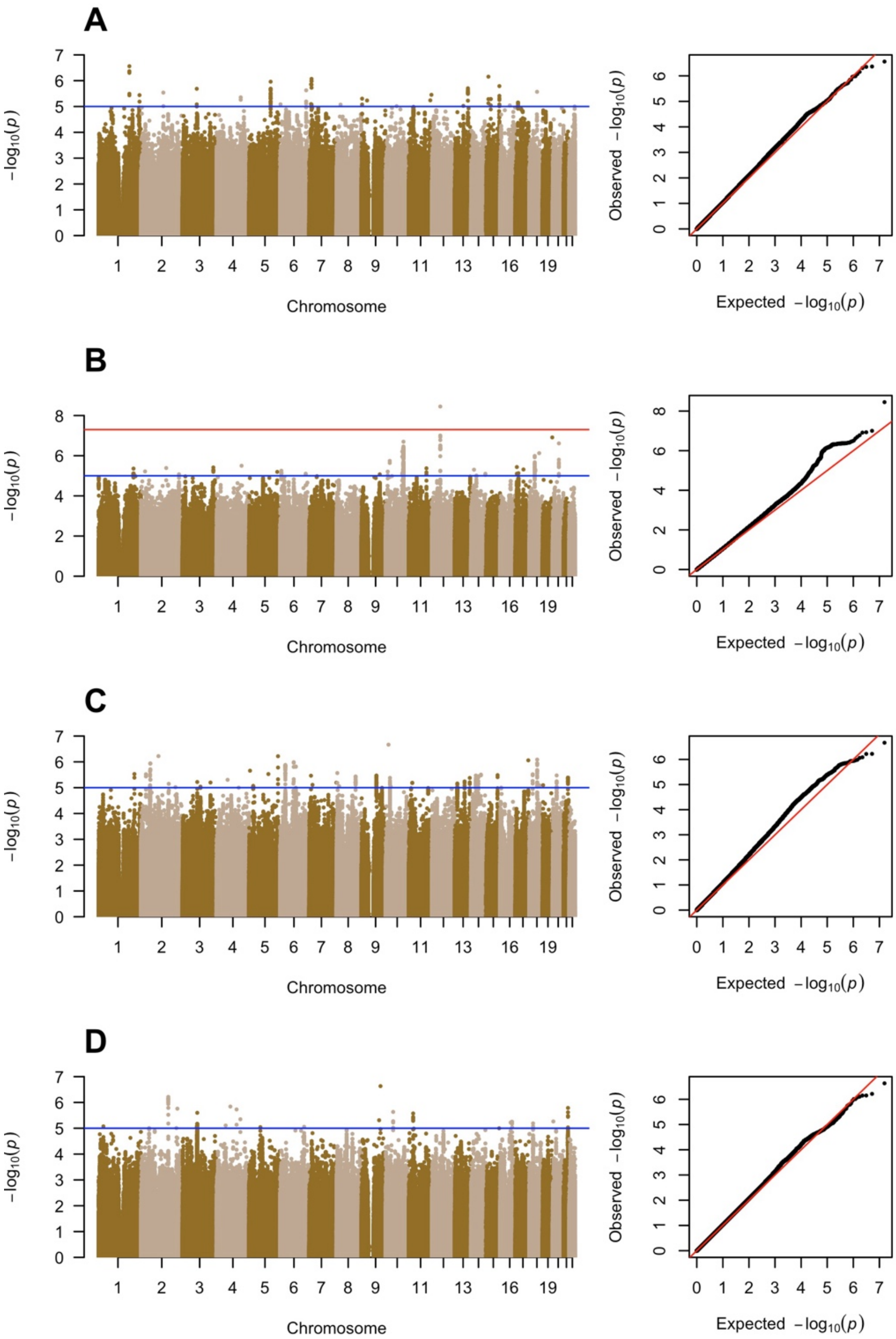

Supplementary Fig. 17 | Manhattan (left) and quantile-quantile (QQ; right) plots for multi-ancestry GWAS meta-analysis of temperament traits in the third year. These plots are for analyses of (A) Emotionality N=56,105, (B) Activity N=56,123, (C) Shyness N=56,095 and (D) Sociability 56,083 in the third year. Manhattan plots show the -$\log_{10}$p-value (y-axis) for the association between each of the genetic variants included in the meta-analysis and the phenotype. P-values (two-tailed) are from the standard-error weighted fixed-effects meta-analysis in METAL[2] and are not adjusted for multiple comparisons,. The horizontal red line indicates genome-wide significant threshold ($p=5x10^{-8}$), the blue line indicates suggestive significance threshold ($p=1x10^{-5}$). Variants are ordered by chromosome and base pair location (x-axis). QQ plots show the same -$\log_{10}$p-values against the expected -$\log_{10}$p-values (red line).

18. Multi-ancestry polygenic score analyses

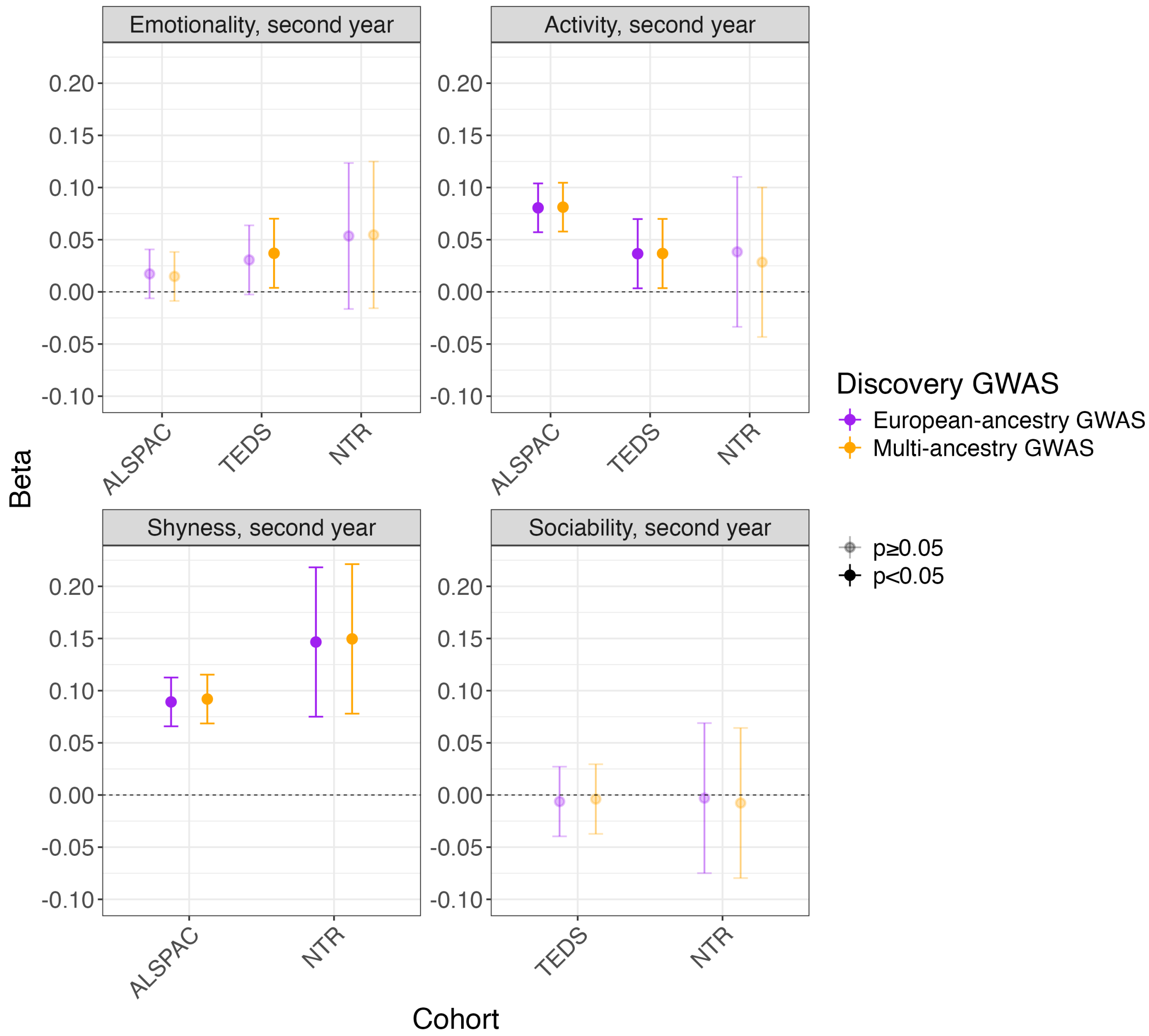

Supplementary Fig. 18 | Polygenic score (PGS) prediction estimates into target samples for temperament in the second year, using multi-ancestry meta-analysis for training data. PGS standardised beta coefficients of the linear regression models testing for associations between the four second-year EAS temperament traits and their polygenic scores, where the polygenic scores are calculated using either the European-ancestry GWAS meta-analysis or the multi-ancestry GWAS meta-analysis. Target samples were ALSPAC (Emotionality N=6,965, Activity N=6,966, Shyness N=6,955), TEDS (Emotionality N=3,473, Activity N=3,473, Sociability N=3,432) and NTR (Emotionality, Activity, Shyness and Sociability N=733). Standardised beta coefficients ± 95% CI (y-axis) are shown for each of the different analyses (x-axis). The points and error bars are solid when the p-value (two-tailed) for the beta-coefficient was $p<0.05$ and translucent for $p \geq 0.05$. The colour of the point indicates the GWAS training data that was used for calculating PGS weights, with purple indicating only European-ancestry samples, and orange indicating multi-ancestry. P-values are uncorrected for multiple comparisons.

19. Phenotypic distribution of the cross-age temperament phenotypes in MoBa

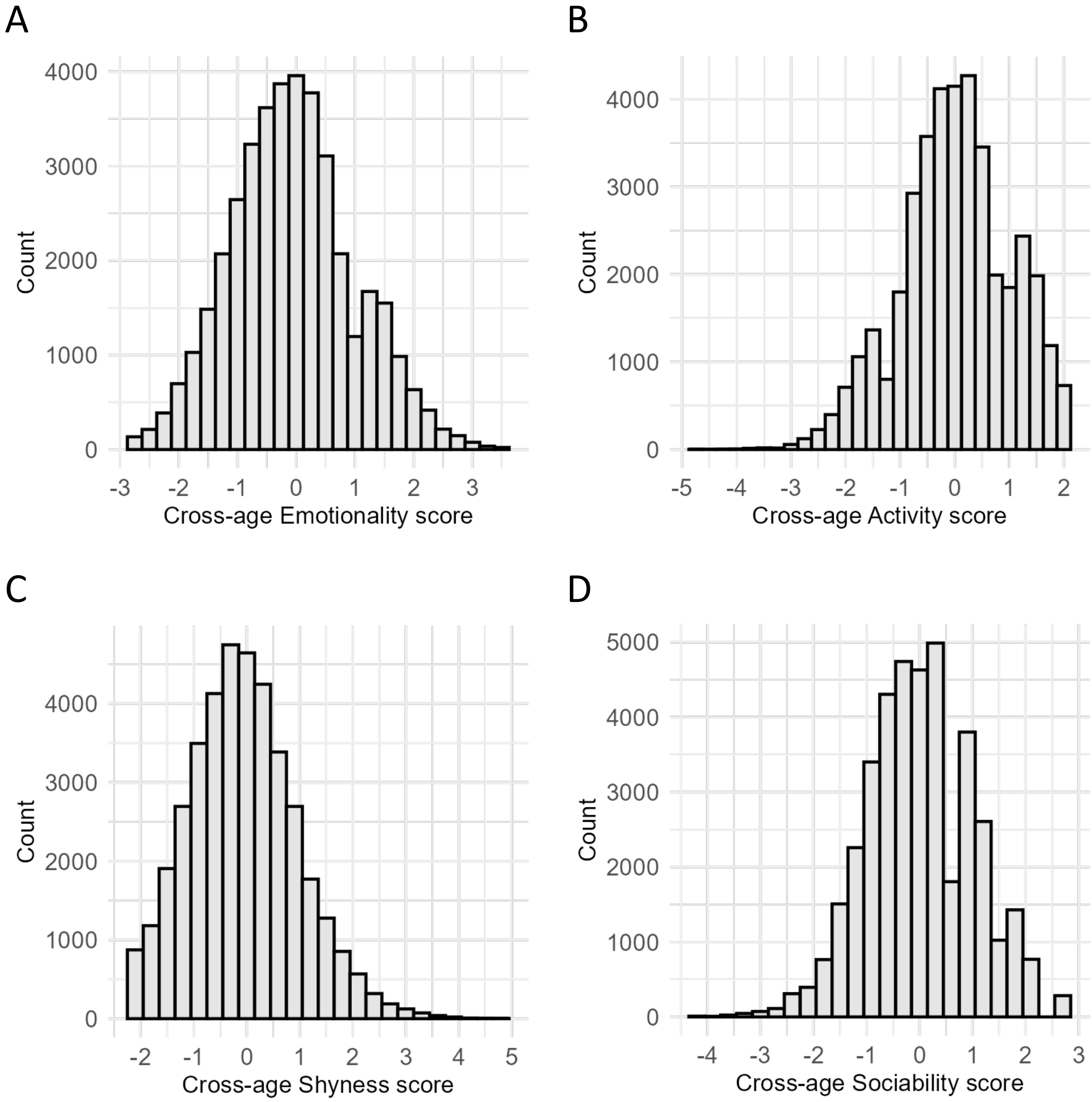

Supplementary Fig. 19 | Histograms for the cross-age temperament phenotypes in the MoBa cohort. The phenotype is calculated as a mean across the measurement taken in the second year and the third year. After taking the mean, the score was standardised. Panels show histograms for (A) Emotionality N=39,234, (B) Activity N=39,274, (C) Shyness N=39,238 and (D) Sociability N=39,290.

20. Phenotypic distribution of the cross-age temperament phenotypes in ALSPAC

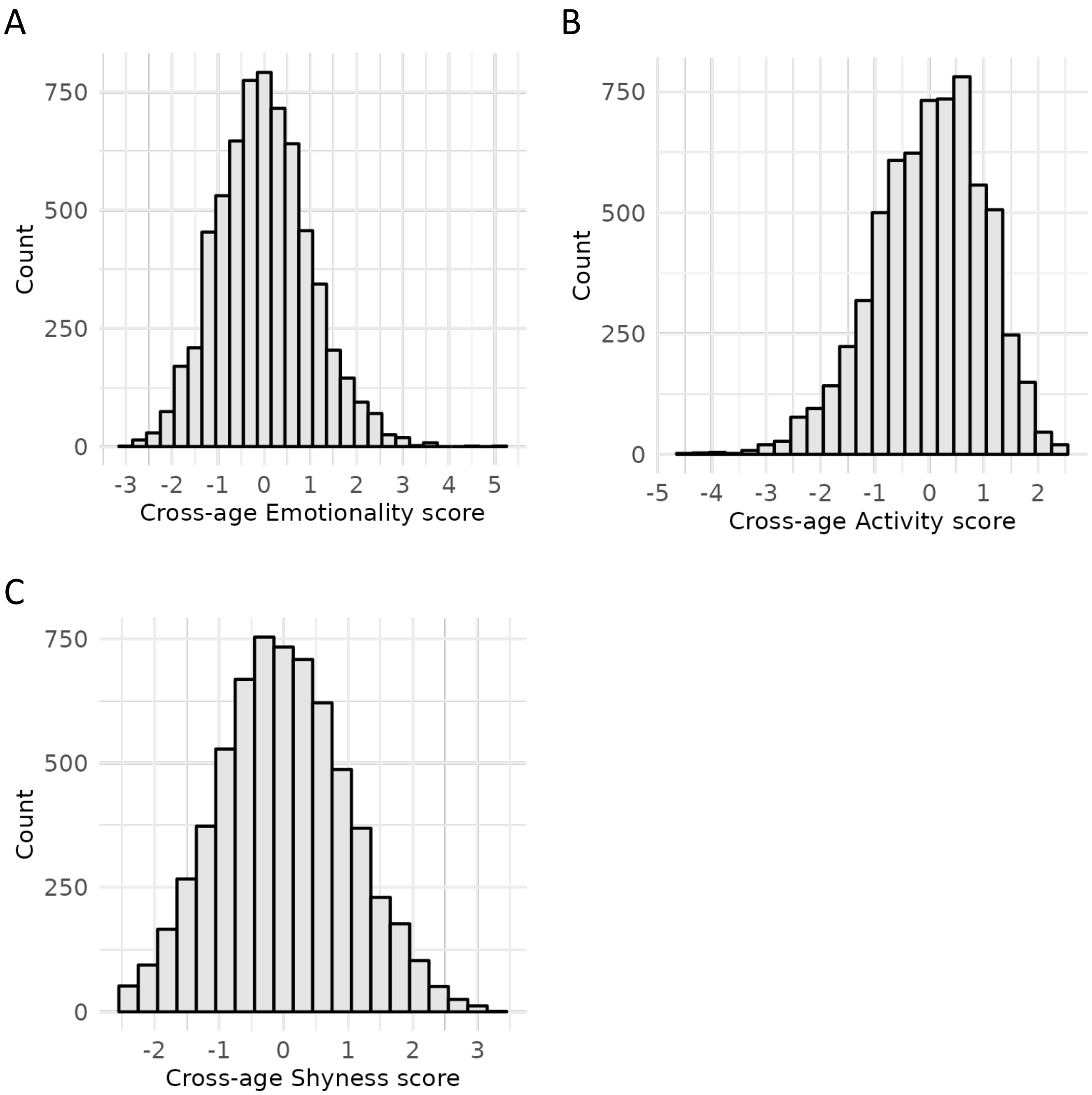

Supplementary Fig. 20 | Histograms for the cross-age temperament phenotypes in the ALSPAC cohort. The phenotype is calculated as a mean across the measurement taken in the second year and the third year. Scores were standardised before taking a mean because the phenotype questionnaires at the two times had different numbers of items and would therefore be unevenly weighted if not standardised. The mean score was then standardised again once calculated. Panels show histograms for (A) Emotionality N=6,423, (B) Activity N=6,425 and (C) Shyness N=6,418.

21. Phenotypic distribution of the cross-age temperament phenotypes in TEDS

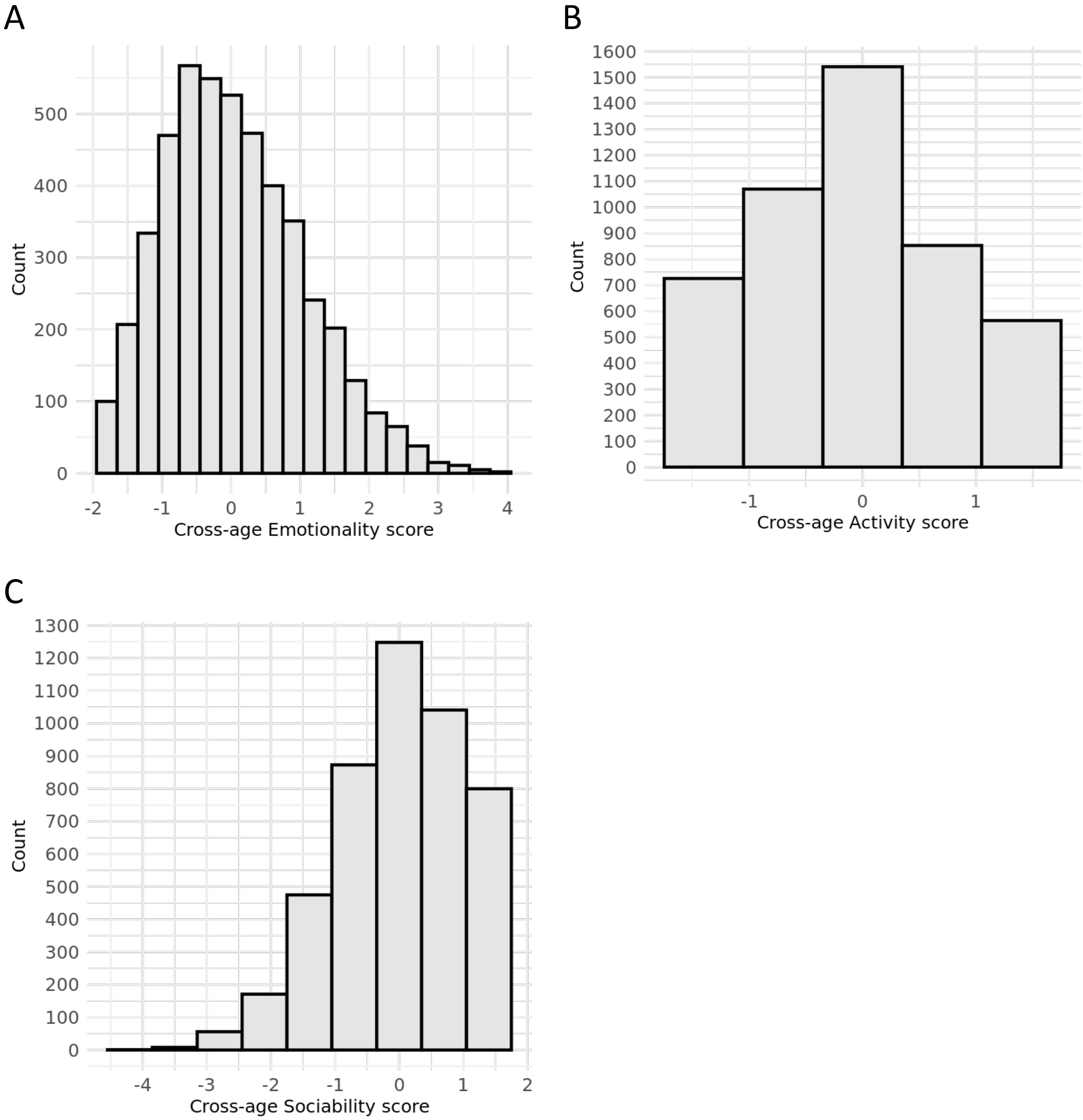

Supplementary Fig. 21 | Histograms for the cross-age temperament phenotypes in the TEDS cohort. The phenotype is calculated as a mean across the measurement taken in the second year and the third year. Scores are standardised. Panels show histograms for (A) Emotionality N=4,769, (B) Activity N=4,754 and (C) Sociability N=4,673.

Supplementary Notes

1. Heritability trend increase

Within each of the four temperament traits, h^2_{SNP} estimates were higher in the cross-age analyses than the specific-age analyses, though most 95% confidence intervals overlapped. We investigated possible reasons for this trend. The higher h^2_{SNP} point estimates in the cross-age analyses could be attributed to the added stability introduced into the phenotype calculation when taking a mean across two different ages. In addition, it has been shown that SNP heritability estimates tend to decrease as the number of samples in a GWAS meta-analysis increases[7], so this trend may be attributed to the cross-age GWAS meta-analyses including fewer cohorts than the specific-age GWAS meta-analyses. Therefore, in exploratory analyses we tested this by conducting the within-year analyses on fewer samples i.e., including only the samples that were included in the cross-age analyses.

Supplementary Fig. 22 shows the h^2_{SNP} estimates and 95% confidence intervals for each of the phenotype meta-analyses, at each age, and split according to how many samples were included. We found that if the specific-age analyses were restricted to only include the samples included in the corresponding cross-age analyses, that the h^2_{SNP} did not tend to increase to the magnitude of that in the cross-age analyses. Thus, results suggest the trend of the cross-age meta-analyses having higher h^2_{SNP} point estimates was not attributable to the reduction in the number of samples in the cross-age analyses. Therefore, the higher h^2_{SNP} point estimates in the cross-age analyses are likely due to the added stability introduced into the phenotype calculation when taking a mean across two different age, which leads to higher h^2_{SNP} point estimates.

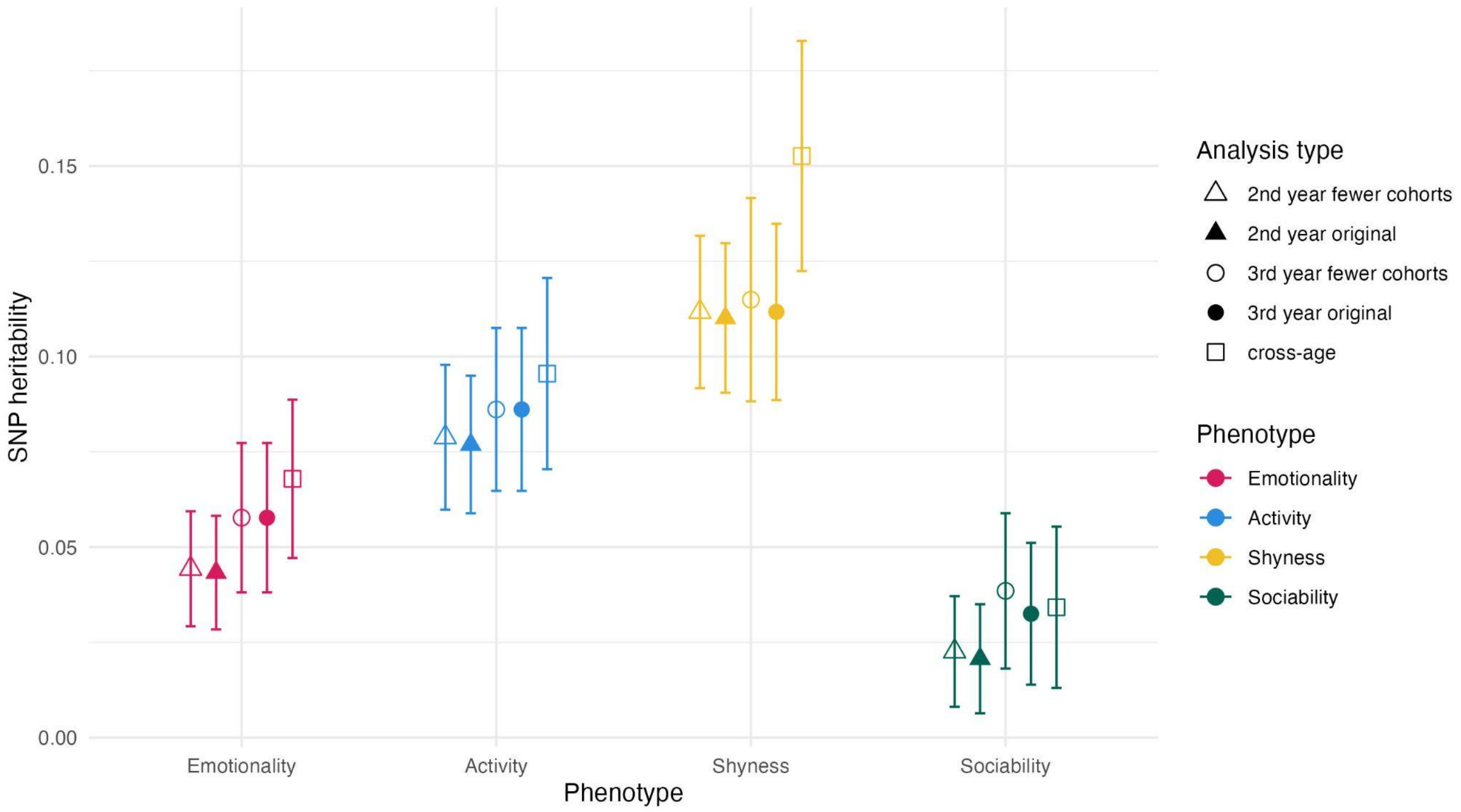

Supplementary Fig. 22 | Comparison of the h^2_{SNP} values for each of the phenotypes, split by age, and by how many cohorts were included in the analysis. LDSC[8] SNP-based heritability (h^2_{SNP}) estimates ± 95% CI for GWAS meta-analyses of infant temperament traits, Emotionality (pink), Activity (blue), Shyness (yellow) and Sociability (green), including different numbers of cohorts in the analysis. Triangles represent analyses of the phenotype in the second year, circles represent analyses of the phenotype in the third year, and squares represent analyses of the phenotype across the two years. Shapes that are not filled in are the same analyses but repeated with only the cohorts that were in the cross-age analysis. Sample sizes for the meta-analyses were: Emotionality 2nd year fewer cohorts N=67,761, 2nd year original N=69,194, 3rd year fewer cohorts N=55,262, 3rd year original N=55,262 and cross-age N=50,426; Activity 2nd year fewer cohorts N=67,795, 2nd year original N=72,663, 3rd year fewer cohorts N=55,280, 3rd year original N=55,280 and cross-age N=50,453; Shyness 2nd year fewer cohorts N=62,230, 2nd year original N=63,663, 3rd year fewer cohorts N=49,587, 3rd year original N=55,252 and cross-age N=45,656; Sociability 2nd year fewer cohorts N=60,797, 2nd year original N=62,230, 3rd year fewer cohorts N=48,390, 3rd year original N=55,240 and cross-age N=43,963.

2. Heterogeneity analyses

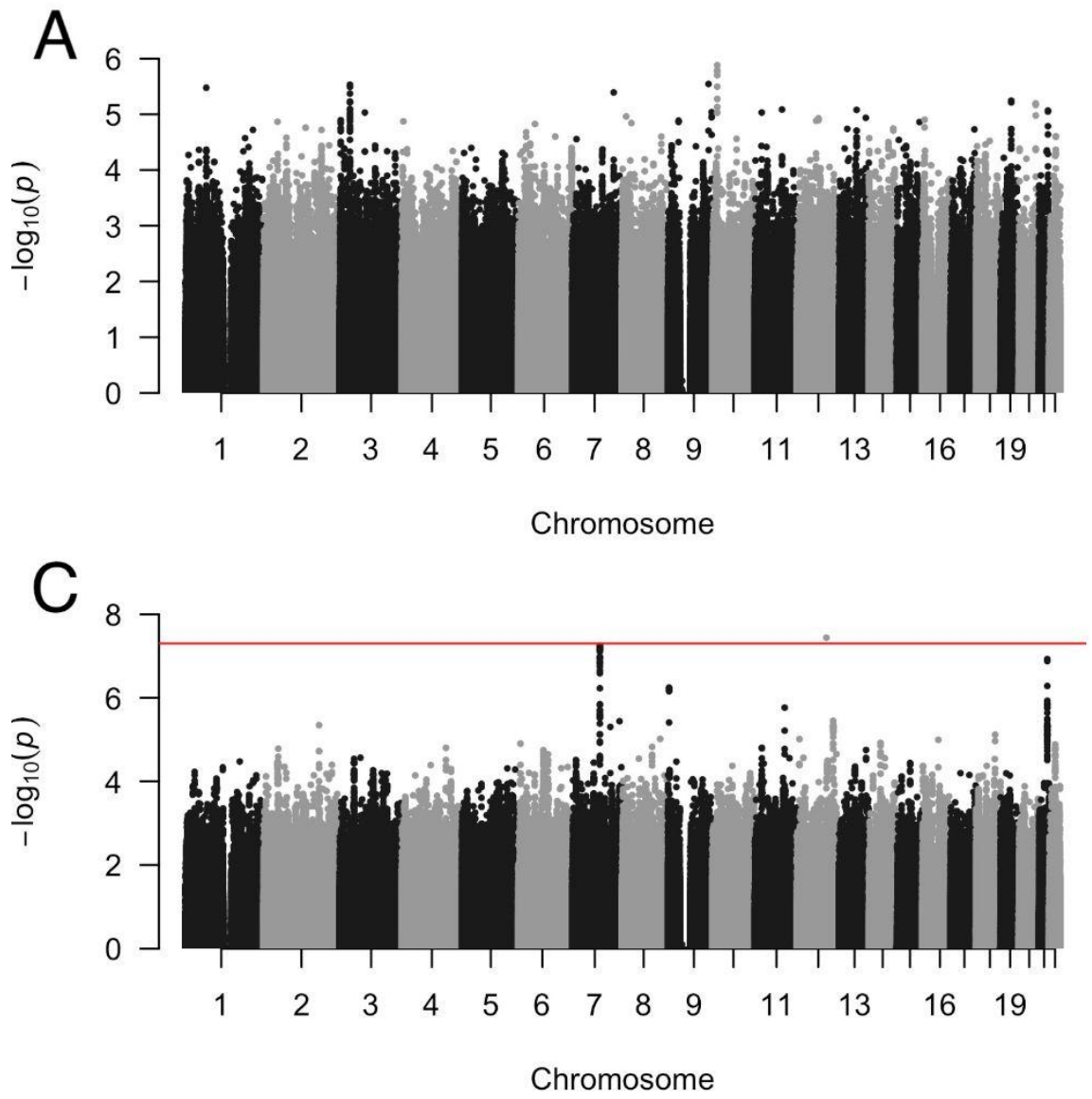

Supplementary Fig. 23 | Plots of heterogeneity test statistics for each of the four phenotypes in the cross-age GWAS analyses. Panels represent heterogeneity analysis plots for the GWAS meta-analyses of (A) Emotionality N=50,426, (B) Activity N=50,453, (C) Shyness N=45,656 and (D) Sociability N=43,963 (cross-age). The red line indicates genome-wide significance ($p=5x10^{-8}$), y-axis represents the -log10 of the p-value for the I^2 test of heterogeneity across cohorts included in the meta-analyses, calculated using METAL[2]. P-values are not corrected for multiple comparisons.

In the Shyness cross-age GWAS, there was one SNP that reached significance (rs71456572, chr12:93121658, GRCh37, $\chi^2(1) = 30.34$, $p = 3.63x10^{-8}$, $I^2 = 96.7\%$, 95% CI=[83.44% , 100%]; Supplementary Fig. 23C). The Shyness cross-age meta-analysis was contributed to by two samples: MoBa and ALSPAC. For this SNP, there was a significant genetic signal in the ALSPAC GWAS but not in the MoBa GWAS. This SNP did not reach genome-wide significance in the meta-analysis.

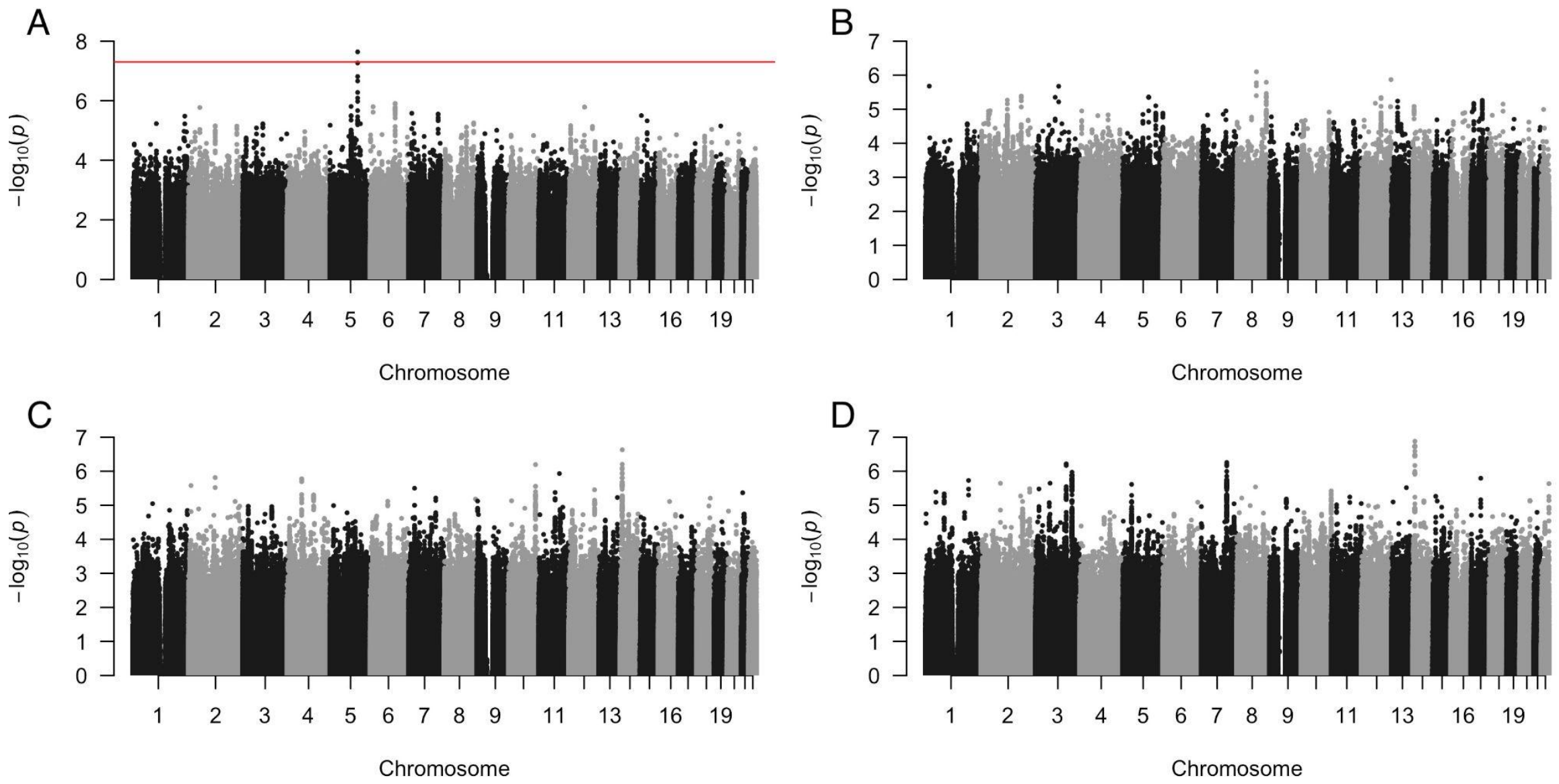

Supplementary Fig. 24 | Plots of heterogeneity test statistics for each of the four GWAS of temperament in the second year. Panels represent heterogeneity analysis plots for (A) Emotionality N=69,194, (B) Activity N=72,663 , (C) Shyness N=63,663 and (D) Sociability N=62,230 in the second year. The red line indicates genome-wide significance ($p=5x10^{-8}$), y-axis represents the -log10 of the p-value for the I^2 test of heterogeneity, across cohorts included in the meta-analyses, calculated using METAL[2]. P-values are not corrected for multiple comparisons.

Of the four phenotypes in the GWAS analysis of temperament in the second year, heterogeneity was found at one locus in the Emotionality analysis (rs33887, chr5: 123751831, GRCh37, $\chi^2(3) = 38.43$, $p = 3.30x10^{-8}$, $I^2 = 92.2\%$, 95% CI=[94.57%, 99.90%]; Supplementary Fig. 24A). This meta-analysis was contributed to by the MoBa, ALSPAC, TEDS and NTR samples. In the locus that showed heterogeneity, there was a genome-wide significant signal in the NTR sample but not the other samples. This locus did not reach significance in the meta-analysis.

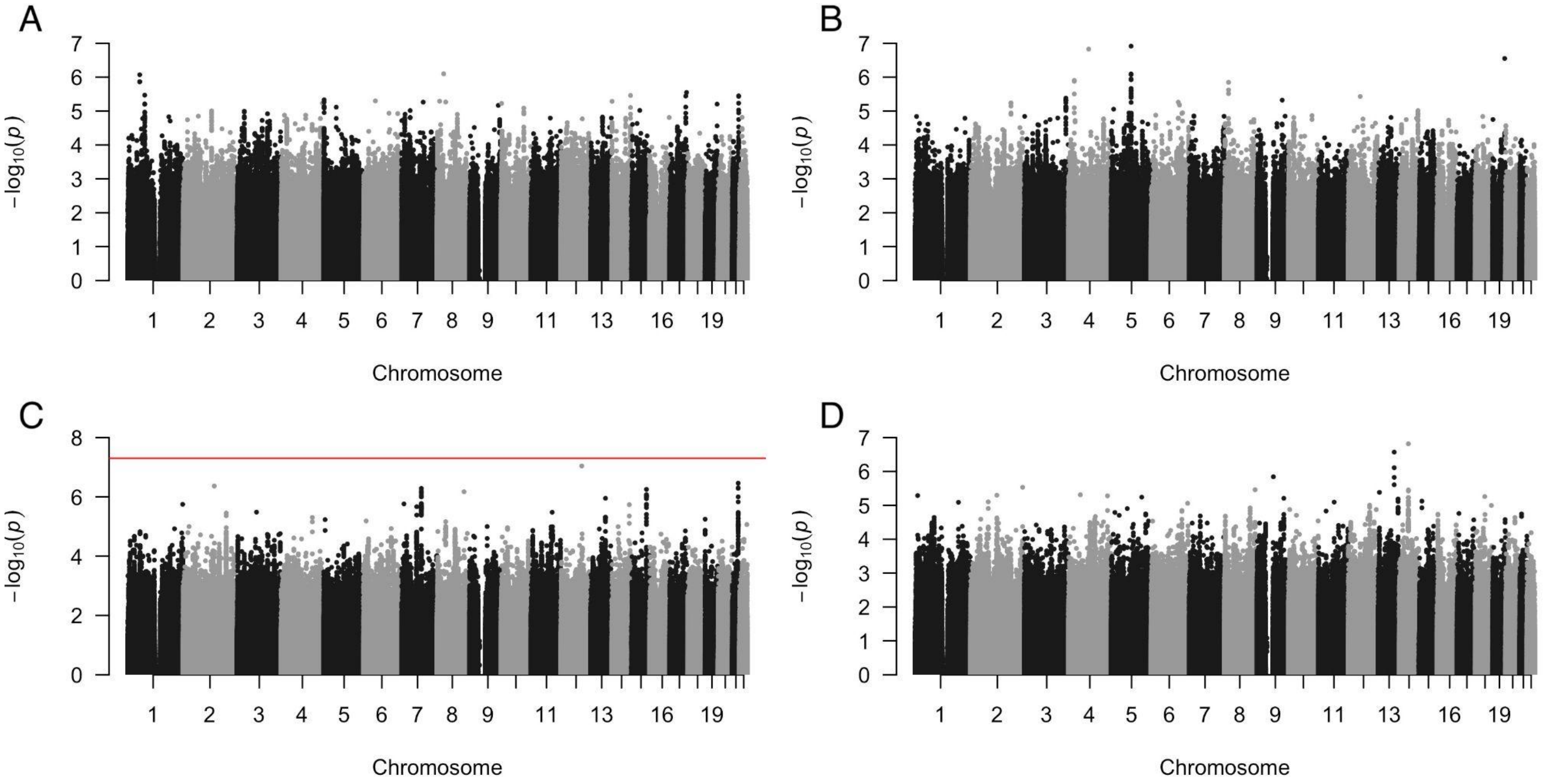

Supplementary Fig. 25 | Plots of heterogeneity test statistics for each of the four GWAS of temperament in the third year. Panels represent heterogeneity analysis plots for (A) Emotionality N=55,262, (B) Activity N=55,280, (C) Shyness N=55252 and (D) Sociability N=55,240 in the third year. The red line indicates genome-wide significance ($p=5x10^{-8}$), y-axis represents the -log10 of the p-value for the I^2 test of heterogeneity, across cohorts included in the meta-analyses, calculated using METAL[2]. P-values are not corrected for multiple comparisons.

In the meta-analyses of temperament in the third year, no genome-wide significant heterogeneity was found (Supplementary Fig. 25).

3. Univariate and bivariate causal mixture modelling

Of the four temperament GWASs, only Shyness met the criterion for applying MiXeR modelling (h^2_{SNP} x N > 6000)[9]. We applied univariate[9] and bivariate[10] Gaussian mixture modelling to the pairs of traits that included Shyness, for which the LDSC genetic correlation reached significance at a false discovery rate (FDR) of 0.05. The differential AIC values for the univariate models were all positive except for Shyness in the third year so this phenotype was not included in the bivariate MiXeR models.

Using univariate MiXeR modelling , we calculated the polygenicity of cross-age Shyness to be 8572.22 SNPs (SD=1547.58 SNPs) meaning that this many SNPs contribute to 90% of the LDSC estimated SNP heritability. Univariate MiXeR analysis estimated the polygenicity of Shyness in the second year to be 6500.79 SNPs (SD=933.49 SNPs).

The AIC values comparing the MiXeR model to the 'minimal model' were positive for the bivariate models between cross-age Shyness and ADHD, BMI and AOW, and between Shyness in the second year and ADHD and extraversion; other models were not supported by the differential AIC values. Full details of the bivariate MiXeR model statistics are in Supplementary Table 16.

From the bivariate MiXeR models, we considered the polygenic overlap as the portion of the variants contributing to the polygenicity of Shyness that are shared with the other trait being analysed (Supplementary Fig. 26). Of the three bivariate models that included cross-age Shyness, the largest amount of polygenic overlap was found between Shyness and AOW, where there was a 94.78% polygenic overlap. Of these variants, 55.99% were concordant in effect direction. Between Shyness and ADHD, there was 86.49% polygenic overlap, of which 40.53% variants were of concordant effect direction. Lastly, of the three models including cross-age Shyness, there was the least amount of polygenic overlap (38.8%) between Shyness and BMI, of which 35.8% was in concordant effect direction. Of the two bivariate MiXeR models including Shyness in the second year, there was a similar amount of polygenic overlap with both ADHD (96.54%) and Extraversion (93.46%). For Shyness in the second year and ADHD, the polygenic overlap had 41.77% concordance of SNP effects. The polygenic overlap between Shyness in the second year and Extraversion had 36.09% concordance of SNP effects.

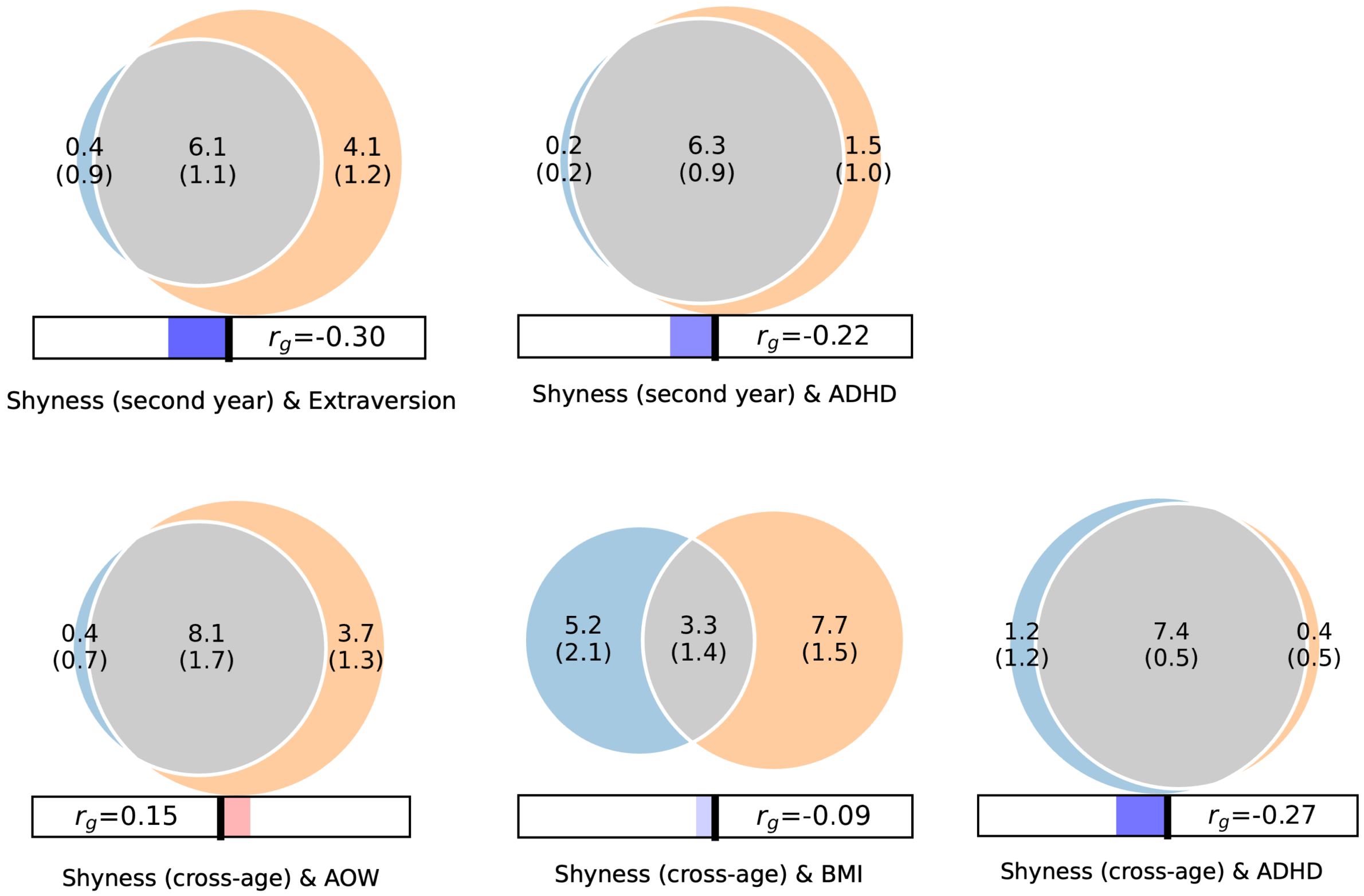

Supplementary Fig. 26 | Bivariate MiXeR[10] plots for infant second-year and cross-age Shyness. Venn diagrams present the polygenic overlap between second-year Shyness and Extraversion and attention deficit, hyperactivity disorder (ADHD), and cross-age Shyness and age at onset of walking (AOW), body mass index (BMI) and ADHD. Blue sections represent variants unique to Shyness (second-year or cross-age, indicated beneath the Venn diagram), orange sections represent the variants unique to the other phenotype (also indicated beneath the Venn diagram) and grey sections represent the degree of polygenic overlap between the two phenotypes. Numbers of unique and overlapping variants are given in thousands in each section, with the standard error in parentheses. Below each Venn diagram is a bar showing the LDSC genetic correlation (r_g), with 0 in the centre represented by a black line. The red or blue bar is proportional in size to the genetic correlation and extends to the left in blue if this is negative and to the right in red if it is positive. These LDSC genetic correlations are calculated by the MiXeR software after the data preparation according to the guidelines from MiXeR developers and as such might bear slight differences from the genetic correlations calculated using LDSC presented in this paper. Sample sizes for the GWAS were: second year Shyness N=63,663, cross-age Shyness N=45,656, Extraversion N=298,772, ADHD Ncases=38,691, Ncontrols=186,843, AOW N=70,560, BMI N=795,640.

4. Preregistration deviations

This study was preregistered (https://osf.io/2yznd). Where the analyses deviated from this pre-registration or where unregistered steps were added, these have been outlined in Supplementary Table 24. This pre-registration outlined analysis plans for a whole project rather than a specific publication and, as such, include a phenotype that was not analysed in the present GWAS study, namely "Fussiness" under age 1.

Supplementary Table 24. Preregistration Deviations. Details of deviations from the original preregistered analysis plans, using previously published table template[11].

<table>
<tr><th colspan="6">Deviations</th></tr>
<tr><th>#</th><th colspan="2">Details</th><th>Original Wording</th><th>Deviation Description</th><th>Reader Impact</th></tr>
<tr><td rowspan="3">1</td><td>Type</td><td>Analysis</td><td rowspan="3">Meta-analysis of GWAS effects will be performed using METAL, including only variants with N>10,000</td><td rowspan="3">"A filter was applied such that variants were required to have, at the least, an N totalling either the summed N of the cohorts not including MoBa, or 10,000, whichever was lower."
It was originally assumed that the smaller cohorts would have a combined sample of 10,000 but some did not have this after QC, so this lower filter was applied in these cases</td><td rowspan="3">This affected the GWAS analyses of Shyness in the second year, and Shyness and Sociability in the cross-age analyses. These will include SNPs for which the combined sample size is N<10,000, for which there may be less power to detect significant effects than other SNPs with N>10,000.</td></tr>
<tr><td>Reason</td><td>New Knowledge</td></tr>
<tr><td>Timing</td><td>After data access</td></tr>
<tr><td rowspan="3">2</td><td>Type</td><td>Data preparation</td><td rowspan="3">The following filters will be applied to the data, as per the SOP (https://osf.io/rcex4). Individuals are excluded from the GWAS if they meet the following conditions:
• excess autosomal heterozygosity (|Fhet|<0.2 or < 3SD),
• mismatch between self-reported and genetic sex determined as excessive X-chromosome homozygosity in individuals classified as males,
• XXY genotype and other aneuploidies, duplicate sample,</td><td rowspan="3">Certain filter thresholds were altered in specific cohorts due to specificities of the dataset:
Individual filters:
• excess autosomal heterozygosity (|Fhet|<0.2 or < 3SD) – NTR filter was |Fhet|<0.1, TMM BirThree no heterozygosity filter was applied
• mismatch between self-reported and genetic sex determined as excessive X-chromosome homozygosity in individuals classified as males – Applied as pre-registered in all cohorts with available X-chromosome data</td><td rowspan="3">These changes concerned the NTR, TMM BirThree, TEDS and DCHS quality control and therefore the SNPs included in these individual cohort GWASs. These differences were necessary to allow for comparable analytic strategies, since the genotyped cohort data differ in structural ways. In all cohorts, pre- and post-imputation processing was performed by expert teams of analysts prior to data access for the present study; the filters used in these processing steps differed between cohorts and therefore the post-imputation QC that was performed on the datasets after data access for this</td></tr>
<tr><td>Reason</td><td>New knowledge</td></tr>
<tr><td>Timing</td><td>After data access</td></tr>
</table>

			• the sample does not cluster with the defined populations of the ancestry reference panel passed on PCA.<br>• their individual genotyping rate is < 95%,<br>• the key phenotype and all relevant covariates are not available for the individual,<br><br>Autosomal SNPs will be excluded from the GWAS if they do not pass the following quality control filters:<br>• Imputation score <0.4,<br>• minor allele frequency (MAF) <0.5%,<br>• failure of Hardy-Weinberg Equilibrium (HWE) exact test at p<1 x 10-6,<br>• call-rate <98%.	• the sample does not cluster with the defined populations of the ancestry reference panel passed on PCA.<br>• their individual genotyping rate is < 95% - NTR filter was 90%; DCHS filter was 98%<br><br>SNP filters:<br>• Imputation score <0.4 – MoBa and TEDS filter was INFO<0.8 and DCHS filter was INFO<0.3<br>• minor allele frequency (MAF) <0.5% - TMM and DCHS filter was MAF<1%<br>• call-rate <98% - TMM filter was 99% and DCHS filter was 95%	project differed, to suit the data. All cohort teams were requested to provide a readme document with key information on their data and pre-processing choices that allowed for the judgement of whether necessary quality checks were performed.<br><br>All summary statistics underwent further QC to further remove differences between cohorts: low (<0.8) imputation score SNPs were removed using GWASinspector[12] and an effect allele frequency (EAF<1%) filter was applied to remove SNPs with low allele frequency during the meta-analysis in METAL[2]. Therefore, these differences would have had only minor, if any, impacts on the final meta-analytic results.
3	Type	Analysis	Stratified LDSC heritability analyses to estimate the heritability partitioned by functional annotation and cell type	These analyses were not performed.	This will impact the reader, as stratified LDSC results are not presented in the manuscript
	Reason	Plan not possible			
	Timing	After data access			

Unregistered Steps

#	Details		Original Wording	Unregistered Step Description	Reader Impact
1	Type	Variables	None - not addressed in preregistration	We preregistered to analyse infant temperament during two age ranges: between 13-27 and 28-40 months respectively.<br>Additional analyses included taking a mean of the temperament measures from these two ranges	We have added this method for calculating the phenotype as a mean with the aim of capturing stability in the phenotype and of reducing measurement error. Prior literature suggests that the power for heritability estimates and gene
	Timing	After data access			

				and conducting GWAS analyses of these “cross-age” measures.	discovery are improved by calculating the phenotype as a mean in this way.
2	Type<br>Timing	Analysis<br>After data access	“Genome-wide genetic correlation will be estimated for i) physical health indicators (e.g., birth weight, adult BMI), ii) neurodevelopmental conditions and cognitive traits (e.g., autism, ADHD, academic achievement, and intelligence) and iii) psychiatric disorders and cortical phenotypes.“	Genetic correlations with personality traits added after pre-registration when a large, well-powered GWAS of the big-5 personality traits became available (Gupta et al., 2024).<br>Genetic correlations with birth weight and cortical phenotypes were not included because of the limited power of the discovery GWASs for this trait.	This addition has little impact on the reader’s interpretation of the results. These genetic correlations were interpreted using the same inference criteria as the other genetic correlations. The omission of low-powered GWAS from genetic correlation analyses will mean that readers cannot make conclusions about the relationship between infant temperament and this phenotype, but limited power in discovery GWAS summary statistics tends to impact the ability to obtain a precise result and therefore the interpretability of these analyses would have been limited had they been included.
3	Type<br>Timing	Inferential criteria<br>After results known	We preregistered to follow up all bivariate genetic correlations that were significant (95% CI does not cross 0) and that fulfilled the criterion of h^2_{SNP} x N > 6,000, with MiXeR bivariate analyses.	We applied a Benjamini-Hochberg procedure to the bivariate genetic correlation results to control for a false discovery rate at 0.05. Genetic correlations were only further investigated using MiXeR if they fulfilled this additional criterion of significance. This criterion was not pre-registered but was applied to limit false positives being investigated further using MiXeR.	Of the pairs of phenotypes that fulfilled the preregistered criterion (h^2_{SNP} x N > 6,000), none did not also fulfill the additional criterion, so there is no impact to the analyses that were conducted, and the results that the reader sees.

5. Phenotype measures in each cohort

All phenotypes were calculated as prorated sum scores of subscale items, with a requirement of 50% of the items to be completed, else the phenotype for that individual was coded as missing.

Norwegian Mother, Father and Child Cohort Study (MoBa)

The short version of the EAS temperament survey[13] was included in the MoBa data collection sweeps at 18 and 36 months, completed by mothers. Instrument details, including questionnaire items, can be found in the MoBa website https://www.fhi.no/en/ch/studies/moba/. The short version contains three questions from each of the four EAS subscales, each rated on a five-point Likert scale (1=Very typical, 2=Quite typical, 3=Neither/nor, 4=Not so typical, 5=Not at all typical). A Norwegian translation of the questionnaire was used in the MoBa data collection surveys; the four-factor structure of the EAS temperament model has been confirmed in the Norwegian population[14]. Wording changes from the published items that were made in the MoBa data collection survey were as follows:
Emotionality: “Child gets upset easily” (EAS published item) → “Child gets upset or sad easily” (MoBa)
Sociability: “Child finds people more stimulating than anything else” (EAS published item) → “Child finds people more fun than anything else” (MoBa)

Netherlands Twin Register

The NTR study used the EAS scale[13] in the second year questionnaire, which contained five items per subscale rated on a five-point Likert scale. A Dutch translation of the questionnaire was used in the NTR data collection surveys. In NTR, the phenotypes were calculated as prorated (50% completion required) means of the subscale items, which become equivalent to a prorated sum score (used in other cohorts) after standardisation of the values.

ALSPAC

ALSPAC used the EAS temperament survey[13] in the third year questionnaire, with five items per subscale, rated on a five-point Likert scale (1=Not at all like him/her, 2=Not much like him/her, 3=Somewhat like him/her, 4=Quite like him/her, 5=Exactly like him/her).

For the second year questionnaire, the ALSPAC study had scales of Emotionality and Activity subscales from the Carey Toddler Temperament Scale (CTTS)[15], named ‘Mood’ and ‘Activity’, in the original publication. For Emotionality, the following items were used from the ALSPAC questionnaire: ke784, ke779, ke707, ke718, ke723, ke762, ke776, ke768, ke757, ke741, ke729, ke703 (the latter five were reverse coded). For Activity, the following items were used ke701, ke722, ke734, ke744, ke747, ke780, ke710, ke716, ke766 (the latter three were reversed). A Shyness subscale was created from seven individual items that matched the EAS shyness scales in NTR, MoBa and ALSPAC. These items were originally from the ‘Approach’ (6 items, e.g. “The child is shy (turns away or clings to mother) on meeting another child for the first time”) and ‘Adaptability’ (1 item, “The child is still wary of strangers after 15 minutes”) subscales. The items used for Shyness were items ke724, ke736, ke742, ke752, ke769 (all reverse coded), ke777 and ke778 (the latter being from the adaptability subscale). All items from the CTTS were rated on a 5-point Likert scale (1=Almost never, 2=Rarely, 3=Sometimes, 4=Often, 5=Almost always).

TEDS

TEDS did not include published EAS scales, however it included items that matched the EAS items, and these were used to create Emotionality, Activity, Shyness (third year only) and Sociability subscales. The items came from the TEDS behaviour problems questionnaires (the 'Your child at home' section, see https://datadictionary.teds.ac.uk/home.htm). These TEDS items stemmed from the Rutter Children's Behaviour Questionnaire[16], the Strengths and Difficulties Questionnaire (SDQ) (e.g. "Gets on well with other children" for Sociability), Behar Preschool Behaviour Questionnaire[17] (e.g. "Restless, runs about or jumps up and down. Doesn't keep still" for Activity and "Gets on well with other children" for Sociability), the Anxiety Related Behaviours Questionnaire (ARBQ)[18] (e.g. "Tends to by shy or timid" for Shyness), and the EAS temperament survey[13] (e.g. "Takes a long time to warm to strangers" for Shyness). These were all scored on a three-point Likert scale. The following items were used to measure the four temperament traits in TEDS (dataset variable names given which correspond across the two data collection sweeps: Emotionality = cbeh101/2, cbeh121/2, cbeh141/2, cbeh201/2, cbeh331/2; Activity = cbeh021/2; Shyness = canx071/2, canx021/2; Sociability = cbeh111/2 (reversed), cbeh311/2. All TEDS items were scored on a three-point Likert scale (1=Not true, 2=Sometimes true, 3=Certainly true).

Lifelines

For the Activity scale from the Lifelines data, responses to the question "Was your child physically more active, equally active or less active than other children of its own age when it was between 6 months and 3 years old?" were used, with answers on a 5-point ordinal scale, which was reverse-coded (1 = much more active; 2 = a bit more active; 3 = just as active; 4 = a bit less active; 5 = much less active). The Activity data were retrospective report on behaviour between 6 months and 3 years and therefore span the two ages in which Activity is investigated in our study. The midpoint of this age range falls within the second year, so these data have been used in the analyses for the second year.

TMM BirThree and DCHS

The TMM and DCHS cohorts both used the Child Behaviour Checklist (CBCL)[19] to measure infant temperament. Questions were rated on a 3-point Likert scale (0=Not True [as far as you know], 1=Somewhat or sometimes true, 2=Very true or often true). Three items were included for each temperament trait (except for Sociability, which used two). These items were selected for their high similarity to the wording of the EAS questionnaire used in MoBa, for example, "Cries a lot" for Emotionality, "Can't sit still, restless or hyperactive" for Activity, "Too shy or timid" for Shyness, and "Doesn't get along with other children" (reversed) for Sociability. The CBCL item numbers for the items used for each of the four temperament traits were as follows: Emotionality = 13, 33, 85; Activity = 6, 36, 62 (latter reverse coded); Shyness = 37, 73, 98; Sociability = 25, 70 (both reverse coded).

6. Agreement between scales across samples

We conducted a study to test the comparability of the Emotionality, Activity, Shyness and Sociability subscales used in the different cohorts described above (excluding the CBCL used in the diverse ancestry cohorts). This study was approved by the Departmental ethics committee for the School of Psychological Sciences at Birkbeck, University of London (reference 2122016) on 15th March 2022.

We collected new data from 202 parents using the parent-report questionnaires used in the individual studies, and estimated the correlations between the Emotionality, Activity, Shyness and Sociability subscales used in each cohort.

We hypothesised that there would be agreement between the subscales used in the different cohorts within each of the four temperament traits and within each age. For all the different subscales used for each age, all possible bivariate correlations were calculated, though our focus was on within-trait correlations.

Methods

Data collection platform

Data were collected through Gorilla Experiment Builder[20]. This platform administered the information sheet, consent form, and data collection.

Participants

Participants were identified and recruited through the university BabyLab database of volunteer participants, as well as through school parent mailing lists and social media. Participants were offered a £10 online shopping voucher for their time participating.

Quality control of online data

We identified data that were produced by bots (automatic computer programmes) and careless responding post-hoc using two strategies. First, we removed data for which data entry had happened in blocks (i.e. multiple entries in 1 minute)[21]. Second, to identify limited-effort responding[22], we used upper and lower time limit filters for the time taken to complete the questionnaire. For the lower limit, we used the criterion suggested by Buchanan & Scofield[23], which was 15.86 minutes for second year questionnaires, 9.62 minutes for third year questionnaires based on the length of the questionnaires that were used. We used an upper limit of 120 minutes.

Analyses

Analyses were conducted using R[24]. Phenotype scores were calculated as prorated sum-scores of the items within the scale, with a requirement for 50% completion, and scaled to have a mean of 0 and standard deviation of 1. Pairwise Spearman correlations were calculated between all the scales. We used the Benjamini-Hochberg procedure to control the false discovery rate at 0.05. This was applied to all pairwise correlations within each age (both intra- and inter-trait).

Results

The sample sizes were N=118 for the second-year questionnaire data and N=84 for the third-year questionnaire data, after filtering for suspected bots and filtering based on time taken to complete the questionnaire. Intra-trait subscale correlations in the second and third year measures are shown in Supplementary Tables 25 and 26 respectively. In support of our hypothesis, all correlations within each trait and age group were positive and significant at $p<0.0005$, with exception of the intra-scale correlations for Sociability in the second year between the MoBa/TEDS and NTR/TEDS scales which were positive but was not significant at $p<0.001$. The intra-trait subscale correlations ranged from $\rho=0.27$-0.80 (median=0.54) for the second-year questionnaires, and $\rho=0.52$-0.90 (median=0.74) for the third-year questionnaires. In conclusion, there was good agreement between the subscales used for each trait across cohorts.

7. Mean differences in reported phenotype according to rater in Lifelines and ALSPAC

In all cohorts, temperament was reported by mothers. However, in two cohorts, a minor proportion of the infants were rated by another caregiver. Here, we assess if there were significant differences in reported temperament contingent on type of rater.

Lifelines

The phenotype in the Lifelines cohort (Activity in the second year) could be reported by a parent (biological, adoptive, foster or step) or 'other'. For this phenotype, the data were only reported by biological mothers or fathers or 'other'; none of the other categories of rater were endorsed. A Kruskal-Wallis test was used to test for a difference in the mean of the phenotype by rater. We found no statistically significant difference between the Activity phenotype reported by each rater type $\chi^2(2) = 0.10$, $p = 0.95$. There was also no statistically significant difference in the phenotype as reported by the father vs. mother, using a Mann-Whitney U test, W = 496292, $z = -3.03\text{x}10^{-5}$, $p = 0.79$.

ALSPAC

ALSPAC parent-report questionnaires could be completed by one or more of the mother, father or "other". This meant there were seven possible rater 'categories' that could have completed the questionnaire, depending on the combination of these three raters:
1=mother only
2=father only
3=other only
4=mother and father
5=mother and other
6=father and other
7=mother, father and other.

For each of the phenotypes calculated in ALSPAC, we used either a one-way ANOVA or a Kruskal-Wallis test (if the ANOVA assumptions were violated); all tests indicated that there was no significant difference in the rater group means.

For the phenotypes in the second year and Emotionality in the third year, a one-way ANOVA test was used to test for the effect of the rater category on the reported phenotype. These were all non-significant: for the second year phenotype Emotionality $F(5,6872)=2.017$, $p=0.073$, Activity $F(5,6872)=1.427$, $p=0.211$, Shyness $F(5,6862)=0.339$, $p=0.89$ and Emotionality in the third year $F(6,6762)=1.179$, $p=0.314$. For Activity, Shyness and Sociability in the third year, the homogeneity of variances assumption did not hold, so a Kruskal-Wallis test was used to test for a difference in the mean by rater category. These tests were all non-significant: Activity $\chi^2(6) = 12.335$, $p = 0.5491$, Shyness $\chi^2(6) = 3.7469$, $p = 0.7109$ and Sociability $\chi^2(6) = 4.9665$, $p = 0.5481$.

In sum, we found no significant differences in the phenotype according to the caregiver that completed the questionnaire, in either ALSPAC or Lifelines. As such, we included all data (regardless of rater) in the GWAS analyses.

8. Covariates used in each sample GWAS

The table below shows the covariates used in the GWAS in each cohort. The MoBa, TEDS and Lifelines samples were genotyped in multiple batches so for these samples, genotyping batch was included as a covariate. Similarly, in NTR, the genotyping array, for which three were used, was included as a covariate. The TEDS sample also used multiple genotyping arrays, but this variable was collinear with the genotyping batch so was not included in the analysis.

Sample	Covariates
MoBa	Year of birth, sex, genotyping batch, 10PCs
ALSPAC	Age, sex, 10PCs
TEDS	Age, sex, genotyping batch, 10PCs
Lifelines	Age, sex, genotyping batch, 10PCs
NTR	Age, sex, genotyping array, 10PCs
TMM	Age, sex, 10PCs
DCHS	Age, sex, genotyping array, genotyping site, 10PCs

"Age" refers to age at data collection; 10 PCs refers to the first 10 principal components

References

1 Auton, A. *et al.* A global reference for human genetic variation. *Nature* **526**, 68-74 (2015). https://doi.org/10.1038/nature15393

2 Willer, C. J., Li, Y. & Abecasis, G. R. METAL: fast and efficient meta-analysis of genomewide association scans. *Bioinformatics* **26**, 2190-2191 (2010). https://doi.org/10.1093/bioinformatics/btq340

3 Watanabe, K., Taskesen, E., van Bochoven, A. & Posthuma, D. Functional mapping and annotation of genetic associations with FUMA. *Nat. Commun.* **8**, 1826 (2017). https://doi.org/10.1038/s41467-017-01261-5

4 de Leeuw, C. A., Mooij, J. M., Heskes, T. & Posthuma, D. MAGMA: generalized gene-set analysis of GWAS data. *PLoS Comput Biol* **11**, e1004219 (2015). https://doi.org/10.1371/journal.pcbi.1004219

5 GTEx Consortium. The GTEx Consortium atlas of genetic regulatory effects across human tissues. *Science* **369**, 1318-1330 (2020). https://doi.org/10.1126/science.aaz1776

6 Bulik-Sullivan, B. *et al.* An atlas of genetic correlations across human diseases and traits. *Nat Genet* **47**, 1236-1241 (2015). https://doi.org/10.1038/ng.3406

7 Wang, X. *et al.* Polygenic risk prediction: why and when out-of-sample prediction R(2) can exceed SNP-based heritability. *Am J Hum Genet* (2023). https://doi.org/10.1016/j.ajhg.2023.06.006

8 Bulik-Sullivan, B. *et al.* LD Score regression distinguishes confounding from polygenicity in genome-wide association studies. *Nat Genet* **47**, 291-295 (2015). https://doi.org/10.1038/ng.3211

9 Hindley, G. *et al.* Charting the Landscape of Genetic Overlap Between Mental Disorders and Related Traits Beyond Genetic Correlation. *Am J Psychiatry* **179**, 833-843 (2022). https://doi.org/10.1176/appi.ajp.21101051

10 Frei, O. *et al.* Bivariate causal mixture model quantifies polygenic overlap between complex traits beyond genetic correlation. *Nature Communications* **10** (2019). https://doi.org/10.1038/s41467-019-10310-0

11 Willroth, E. C. & Atherton, O. E. Best Laid Plans: A Guide to Reporting Preregistration Deviations. *Advances in Methods and Practices in Psychological Science* **7** (2024). https://doi.org/10.1177/25152459231213802
12 Ani, A., van der Most, P. J., Snieder, H., Vaez, A. & Nolte, I. M. GWASinspector: comprehensive quality control of genome-wide association study results. *Bioinformatics* **37**, 129-130 (2021). https://doi.org/10.1093/bioinformatics/btaa1084
13 Buss, A. H. & Plomin, R. *Temperament: early developing personality traits*. Vol. 3 (Lawrence Erlbaum Associates, 1984).
14 Mathiesen, K. S. & Tambs, K. The EAS Temperament Questionnaire-Factor Structure, Age Trends, Reliability, and Stability in a Norwegian Sample. *Journal of Child Psychology and Psychiatry* **40**, 431-439 (1999). https://doi.org/10.1111/1469-7610.00460
15 Fullard, W., McDevitt, S. C. & Carey, W. B. Assessing Temperament in One- to Three-Year-Old Children. *Journal of Paediatric Psychology* **9** (1984).
16 Rutter, M. A children's behaviour questionnaire for completion by teachers: preliminary findings. *J Child Psychol Psychiatry* **8**, 1-11 (1967). https://doi.org/10.1111/j.1469-7610.1967.tb02175.x
17 Behar, L. The Preschool Behavior Questionnaire. *J Abnorm Child Psychol* **5**, 265-275 (1977). https://doi.org/10.1007/BF00913697
18 Eley, T. C. *et al.* A twin study of anxiety-related behaviours in pre-school children. *J Child Psychol Psychiatry* **44**, 945-960 (2003). https://doi.org/10.1111/1469-7610.00179
19 Achenbach, T. M. & Rescorla, L. A. *Manual for ASEBA School-Age Forms & Profiles*. (University of Vermont, Research Center for Children, Youth, & Families, 2001).
20 Anwyl-Irvine, A. L., Massonnie, J., Flitton, A., Kirkham, N. & Evershed, J. K. Gorilla in our midst: An online behavioral experiment builder. *Behav Res Methods* **52**, 388-407 (2020). https://doi.org/10.3758/s13428-019-01237-x
21 Irish, K. & Saba, J. Bots are the new fraud: A post-hoc exploration of statistical methods to identify bot-generated responses in a corrupt data set. *Personality and Individual Differences* **213** (2023).
22 Meade, A. W. & Craig, S. B. Identifying careless responses in survey data. *Psychol Methods* **17**, 437-455 (2012). https://doi.org/10.1037/a0028085
23 Buchanan, E. M. & Scofield, J. E. Methods to detect low quality data and its implication for psychological research. *Behav Res Methods* **50**, 2586-2596 (2018). https://doi.org/10.3758/s13428-018-1035-6
24 R Core Team. R: A language and environment for statistical computing. *R Foundation for Statistical Computing, Vienna, Austria* (2013). https://doi.org/http://www.R-project.org/
