# Supplementary material for: Genome-wide association studies of infant and toddler temperament in European and multi-ancestry populations
Source: Nat Hum Behav. 2026 Jul 1;10(9):1821–40. doi: 10.1038/s41562-026-02486-5 (PMC13590410; doi:10.1038/s41562-026-02486-5)
Supplement: Supplementary file 2 [file 41562_2026_2486_MOESM2_ESM.pdf]

nature portfolio

Corresponding author(s): Angelica Ronald

Last updated by author(s): Dec 17, 2025

Reporting Summary

Nature Portfolio wishes to improve the reproducibility of the work that we publish. This form provides structure for consistency and transparency in reporting. For further information on Nature Portfolio policies, see our Editorial Policies and the Editorial Policy Checklist.

Statistics

For all statistical analyses, confirm that the following items are present in the figure legend, table legend, main text, or Methods section.

n/a	Confirmed	
☐	☒	The exact sample size (*n*) for each experimental group/condition, given as a discrete number and unit of measurement
☐	☒	A statement on whether measurements were taken from distinct samples or whether the same sample was measured repeatedly
☐	☒	The statistical test(s) used AND whether they are one- or two-sided<br>*Only common tests should be described solely by name; describe more complex techniques in the Methods section.*
☐	☒	A description of all covariates tested
☐	☒	A description of any assumptions or corrections, such as tests of normality and adjustment for multiple comparisons
☐	☒	A full description of the statistical parameters including central tendency (e.g. means) or other basic estimates (e.g. regression coefficient) AND variation (e.g. standard deviation) or associated estimates of uncertainty (e.g. confidence intervals)
☐	☒	For null hypothesis testing, the test statistic (e.g. *F*, *t*, *r*) with confidence intervals, effect sizes, degrees of freedom and *P* value noted<br>*Give P values as exact values whenever suitable.*
☐	☒	For Bayesian analysis, information on the choice of priors and Markov chain Monte Carlo settings
☒	☐	For hierarchical and complex designs, identification of the appropriate level for tests and full reporting of outcomes
☐	☒	Estimates of effect sizes (e.g. Cohen's *d*, Pearson's *r*), indicating how they were calculated

Our web collection on statistics for biologists contains articles on many of the points above.

Software and code

Policy information about availability of computer code

Data collection	No software was used for data collection for the purpose of this study.
Data analysis	MoBa phenotype data analysis was performed using the R package phenotools: https://github.com/psychgen/phenotools<br>Genotype data analysis was performed in PLINK 1.9 https://www.cog-genomics.org/plink/ , PLINK 2 https://www.cog-genomics.org/plink/2.0/, GCTA https://yanglab.westlake.edu.cn/software/gcta/#Overview, R. Summary statistics quality control was performed using the R package GWASinspector http://gwasinspector.com/ . The meta-analysis was performed in METAL https://genome.sph.umich.edu/wiki/METAL. Post-GWAS fine mapping and functional annotation was performed in FUMA (version 1.5.2) and MAGMA (version 1.08) https://fuma.ctglab.nl/ . Colocalization analyses were conducted using R libraries locuscomparer 1.0.0 (https://github.com/boxiangliu/locuscomparer), coloc 5.2.2 (https://CRAN.R-project.org/package=coloc). SNP heritability, genetic correlation and partitioned heritability were estimated using LD score regression https:/ /github.com/bulik/ldsc . The MiXeR program is available here: https:/ /github.com/precimed/mixer. The polygenic scores were calculated using PRS-cs https:/ /github.com/getian107 /PRScs . The scripts for the within and between-family polygenic score analyses are available here: https:/ /github.com/PerlineDemange/GeneticNurtureNonCog/.

For manuscripts utilizing custom algorithms or software that are central to the research but not yet described in published literature, software must be made available to editors and reviewers. We strongly encourage code deposition in a community repository (e.g. GitHub). See the Nature Portfolio guidelines for submitting code & software for further information.

Data

Policy information about availability of data

All manuscripts must include a data availability statement. This statement should provide the following information, where applicable:

- Accession codes, unique identifiers, or web links for publicly available datasets
- A description of any restrictions on data availability
- For clinical datasets or third party data, please ensure that the statement adheres to our policy

The summary statistics of the genome-wide association study of infant temperament traits are available on figshare (https://doi.org/10.6084/m9.figshare.30041575).

Data from the Norwegian Mother, Father and Child Cohort (MoBa) Study and the Medical Birth Registry of Norway used in this study are managed by the National Health Register Holders in Norway (Norwegian Institute of Public Health) and can be made available to researchers, with approval from the Regional Committees for Medical and Health Research Ethics (REC), compliance with the EU General Data Protection Regulation (GDPR) and approval from the data owners. The consent given by the participants is not open to storage of data on an individual level in repositories or journals. Researchers who want access to datasets for replication should apply through https:// helsedata.no/. Access to datasets requires approval from The Regional Committee for Medical and Health Research Ethics in Norway and an agreement with MoBa.
Data from the Netherlands Twin Register (NTR) are available upon request by researchers. Information is available at https://ntr-data-request.psy.vu.nl
Lifelines data may be obtained from a third party and are not publicly available. Researchers can apply to use the Lifelines data used in this study. More information about how to request Lifelines data and the conditions of use can be found on their website at https://www.lifelines-biobank.com/researchers/working-with-us.
The informed consent obtained from ALSPAC (Avon Longitudinal Study of Parents and Children) participants does not allow the data to be made available through any third party maintained public repository. Supporting data are available from ALSPAC on request under the approved proposal number, B3360. Full instructions for applying for data access can be found here: http://www.bristol.ac.uk/alspac/researchers/access/. The ALSPAC study website contains details of all available data (http://www.bristol.ac.uk/alspac/researchers/our-data/).
The TEDS data are accessible to researchers following the procedure outlined in their data access policy (https://www.teds.ac.uk/researchers/teds-data-access-policy/)
The data that support the findings of this study are available from Tohoku Medical Megabank project but restrictions apply to the availability of these data, which were used under license for the current study, and so are not publicly available. Data are however available upon reasonable request and with permission of the Tohoku Medical Megabank project (dist@megabank.tohoku.ac.jp).
The Drakenstein Child Health Study is committed to the principle of data sharing. De-identified data will be made available to requesting researchers as appropriate. Requests for collaborations to undertake data analysis are welcome. More information can be found on their website (http://www.paediatrics.uct.ac.za/scah/dclhs).
eQTL results for the ROSMAP, Mayo TCX, Mayo CER and cortical meta-analysis from Sieberts et al., (2020) are available through the AMP-AD Knowledge Portal: https://www.synapse.org/#!Synapse:syn17015233.

Research involving human participants, their data, or biological material

Policy information about studies with human participants or human data. See also policy information about sex, gender (identity/presentation), and sexual orientation and race, ethnicity and racism.

Reporting on sex and gender	Biological sex was used as a co-variate and for sex-stratified analyses in this study. Sex was determined based on sex chromosome information and self or parent-report.
Reporting on race, ethnicity, or other socially relevant groupings	Genetic ancestry was estimated based on principal component analysis on the cohorts' genotype data following standard procedures (e.g., Marees et al., 2018 doi: 10.1002/mpr.1608, Corefield et al., 2022 doi: 10.1101/2022.06.23.496289).
Population characteristics	Participants were N=56,083-78,894 genotyped children from European, South African and Japanese genetic ancestry from seven cohorts: Norwegian Mother, Father and Child Cohort Study (MoBa, N=39,234-55,337), Netherlands Twin Register (NTR, N=1,433), Lifelines multi-generational prospective population-based birth cohort study (N=3,435), Avon Longitudinal Study of Parents and Children (ALSPAC, N=6,418-6,966), Twins Early Development Study (TEDS, N=4,673-5,686), Tohoku Medical Megabank Birth and Three-Generation Cohort Study (TMM BirThree, N=5,618-5,653), Drakenstein Child Health Study (DCHS, N=595-842). Phenotype data were parent-report questionnaires collected when the children were 18 and 36 months for MoBa, at age 2 for NTR children, retrospectively (age 4-18) for Lifelines, at 24 and 38 months for ALSPAC, at ages 2 and 3 for TEDS, at 24 months for TMM BirThree, and at 24 and 42 months for DCHS.<br>Participants were also included from an independent sample of parents, who provided data for the phenotypic study in this project, which looked at concordance between the phenotypic measures used in each of the cohorts above (N=202).
Recruitment	For MoBa, participants were recruited through hospitals, firstly in Bergen starting in 1999, and then expanding to 50 of 52 Norway's hospitals with maternity units.<br>NTR is a population-based cohort of over 200,000 people from across the Netherlands. It consists of twin-families, i.e. twins, their parents, spouses and siblings aged between O and 99 years at recruitment. NTR started around 1987 with new-born twins and adolescent and adult twins.<br>The Lifelines study was established in 2006 and asked all the general medical practitioners operating in the provinces of Friesland, Groningen and Drenthe to invite their patients aged between 25 and 50 to take part in the study unless the patient met one of five exclusion criteria (as determined by the practitioner): a) having a severe psychiatric or physical illness (such that the individual was not fully capable to make rational decisions), b) life expectancy of less than 5 years, c) being unable to complete a Dutch language questionnaire, d) a lack of ability in the Dutch language, or e) not being able to visit their medical practitioner (http://wiki-lifelines.web.rug.nl/doku.php?id=cohort).<br>For ALSPAC, pregnant people were invited to participate in the study if they has an expected delivery date between 1st April

1991 and 31st December 1992. The children from these pregnancies formed the G0 generation for this study.
TEDS participants were twins born in England and Wales between 1994–1996.
TMM BirThree was established in 2012 after the Great East Japan Earthquake (GEJE), and participants were recruited from the Miyagi prefecture where this earthquake took place. Participants were recruited between 2013-2017, if their expected delivery date was after 1st February 2014.
DCHS participants were recruited between March 2012 and March 2015; pregnant women were recruited during routine antenatal care appointments at one of two clinics in Drakenstein, South Africa: Mbekweni or TC Newman clinic. Eligibility criteria were that mothers were 18 years or older, planned to attend their antenatal care appointments at one of the two recruitment clinics and were intending to remain in the area for at least one year.
Participants for the phenotypic study were recruited through a university BabyLab database, on social media, and through school parent mailing lists.

Ethics oversight

This study and the related secondary data analysis were approved by the Departmental Ethics Committee of the Psychological Science Department of Birkbeck, University of London on 27th October 2020 (reference number 2021007).
MoBa and the related data collection was authorised by a licence from the Norwegian Data Protection Agency and an approval from the The Regional Committees for Medical and Health Research Ethics (REK). MoBa is regulated by the Norwegian Health Registry Act. The current study was approved by REK (2016/1702).
Informed consent for NTR was obtained from parents or guardians. The study was approved by the Central Ethics Committee on Research Involving Human Subjects of the VU University Medical Centre, Amsterdam, an Institutional Review Board certified by the U.S. Office of Human Research Protections (IRB number IRB00002991 under Federal-wide Assurance-FWA00017598; IRB/institute codes, NTR 03-180). For Lifelines, participants in Lifelines gave written consent prior to physical examination. The study is conducted according to the principles of the Declaration of Helsinki and in accordance with the UMCG research code and is approved by the medical ethical committee of UMCG (document number METC UMCG METc 2007/152). ALSPAC received ethical approval from the ALSPAC Ethics and Law Committee and the Local Research Ethics Committees. TEDS was granted ethical approval by the Kings College London Ethics Committee (HR/DP-20/21-22060).
Consent was obtained from participants for every wave of data collection. TMM BirThree Cohort Study was reviewed and approved by the Ethics Committee of ToMMo (2013-1-103-1). DCHS was approved by the Ethics Committee of the Faculty of Health Sciences, University of Cape Town (401/2009).
The phenotypic study was approved by the Departmental ethics committee for the School of Psychological Sciences at Birkbeck, University of London (reference 2122016) on 15th March 2022.

Note that full information on the approval of the study protocol must also be provided in the manuscript.

Field-specific reporting

Please select the one below that is the best fit for your research. If you are not sure, read the appropriate sections before making your selection.

☒ Life sciences ☐ Behavioural & social sciences ☐ Ecological, evolutionary & environmental sciences

For a reference copy of the document with all sections, see nature.com/documents/nr-reporting-summary-flat.pdf

Life sciences study design

All studies must disclose on these points even when the disclosure is negative.

Sample size

Cohorts were invited to take part in the GWAS meta-analysis if they had available phenotype data (infant temperament) and genotype for more than 1,000 individuals. The minimum sample size for participanting in the GWAS meta-analysis was defined a priori and pre-registered on OSF (https://osf.io/2yznd), except for diverse ancestry samples for which this minimum sample size was not needed. This minimum sample size was chosen to maximise the power of the analyses (Visscher et al., 2017). The total sample size for this GWAS meta-analysis was N=56,083-78,894 infants.

Data exclusions

In all the individual cohorts, samples were excluded from the GWAS if they had missing phenotype data, if they presented excess autosomal heterozygosity (except in TMM BirThree where this filter was not applied), mismatch between self-reported and genetic sex (where X-chromosome data were available), XXY genotype and other aneuploidies, individual genotyping rate <90% (<95% for NTR and <98% for DCHS). Duplicate samples were removed. Individuals whose genetically determined ancestry did not overlay with the relevant ancestry cluster based on a reference panel were also excluded.

Replication

There was no replication of the results from this study.

Randomization

There was no randomization involved in this study. This was not a randomized control trial, but an observational design.

Blinding

There was no blinding involved in this study with respect to group allocation, as this was an observational study.

Reporting for specific materials, systems and methods

We require information from authors about some types of materials, experimental systems and methods used in many studies. Here, indicate whether each material, system or method listed is relevant to your study. If you are not sure if a list item applies to your research, read the appropriate section before selecting a response.

Materials & experimental systems

n/a	Involved in the study
☒	☐ Antibodies
☒	☐ Eukaryotic cell lines
☒	☐ Palaeontology and archaeology
☒	☐ Animals and other organisms
☒	☐ Clinical data
☒	☐ Dual use research of concern
☒	☐ Plants

Methods

n/a	Involved in the study
☒	☐ ChIP-seq
☒	☐ Flow cytometry
☒	☐ MRI-based neuroimaging

Plants

Seed stocks	N/A
Novel plant genotypes	N/A
Authentication	N/A
